# Dynamic Evolution of the LPS-Detoxifying Enzyme Intestinal Alkaline Phosphatase in Zebrafish and Other Vertebrates

**DOI:** 10.3389/fimmu.2012.00314

**Published:** 2012-10-12

**Authors:** Ye Yang, Anica M. Wandler, John H. Postlethwait, Karen Guillemin

**Affiliations:** ^1^Institute of Molecular Biology, University of OregonEugene, OR, USA; ^2^Institute of Neuroscience, University of OregonEugene, OR, USA

**Keywords:** zebrafish, intestinal alkaline phosphatase, vertebrate, evolution, microbiota

## Abstract

Alkaline phosphatases (Alps) are well-studied enzymes that remove phosphates from a variety of substrates. Alps function in diverse biological processes, including modulating host-bacterial interactions by dephosphorylating the Gram-negative bacterial cell wall component lipopolysaccharide (LPS). In animals, Alps are encoded by multiple genes characterized by either ubiquitous expression (named *Alpl*s for their liver expression, but a key to proper bone mineralization), or their tissue-specific expression, for example in the intestine (*Alpi*). We previously characterized a zebrafish *alpi* gene (renamed here *alpi.1*) that is regulated by Myd88-dependent innate immune signaling and that is required to prevent a host’s excessive inflammatory reactions to its resident microbiota. Here we report the characterization of two new *alp* genes in zebrafish, *alpi.2* and *alp3*. To understand their origins, we investigated the phylogenetic history of *Alp* genes in animals. We find that vertebrate *Alp* genes are organized in three clades with one of these clades missing from the mammals. We present evidence that these three clades originated during the two vertebrate genome duplications. We show that *alpl* is ubiquitously expressed in zebrafish, as it is in mammals, whereas the other three *alps* are specific to the intestine. Our phylogenetic analysis reveals that in contrast to *Alpl*, which has been stably maintained as a single gene throughout the vertebrates, the *Alpi*s have been lost and duplicated multiple times independently in vertebrate lineages, likely reflecting the rapid and dynamic evolution of vertebrate gut morphologies, driven by changes in bacterial associations and diet.

## Introduction

Alkaline phosphatases (Alps) are a superfamily of metalloenzymes that catalyze the hydrolytic removal of phosphate from a variety of molecules (Millán, 2006). Alps have been extensively studied biochemically, but the full spectrum of their biological functions is not known. In animals, Alps are encoded by multiple genes that can be classified by their expression patterns into two general groups, the tissue-non-specific *Alp*s (known as *Alpl*, for their liver expression, also known as *TNAP* for tissue non-specific alkaline phosphatase), and the tissue-specific *Alp*s [placental *Alp* (*Alpp*), intestinal *Alp* (*Alpi*), etc.]. The best studied biological function of Alps is the role of mammalian Alpl in osteogenesis by promoting bone mineralization, as demonstrated by the hypophosphatasia that results from *ALPL* deficiency in humans and mice (Whyte, 2010). More recently, the *Alpi*s have been implicated in mediating host-bacterial interactions through their ability to dephosphorylate lipid A of the Gram-negative bacterial cell wall component lipopolysaccharide (LPS; Lalles, 2010).

In mammals, Alpi is expressed by intestinal epithelial cells (IECs) and is enriched in vesicles that are actively released from IEC microvillar tips into the intestinal lumen (McConnell et al., 2009; Shifrin et al., 2012). Thus Alpi is located at the interface between the intestinal tissue, the ingesta and the vast microbiota, which suggests its involvement in a variety of biological processes. Recent studies have discovered that Alpi regulates metabolism by controlling the uptake of nutrients such as lipids (Narisawa et al., 2003; Lynes et al., 2011) and calcium (Brun et al., 2012), affects gut physiology by maintaining protective surface microclimate pH in the duodenum (Akiba et al., 2007; Mizumori et al., 2009), and impacts innate immunity by modulating bacterial LPS-induced inflammation (Poelstra et al., 1997b; Bates et al., 2007; Campbell et al., 2010).

Lipopolysaccharide, also commonly referred to as endotoxin, is a component of Gram-negative bacterial cell walls and is abundantly present in the intestinal lumen of animals. LPS is a classic microbial associated molecular pattern (MAMP) and potent inducer of innate immune signaling in both vertebrates and invertebrates (Beutler and Rietschel, 2003). In mammals, LPS binds specifically to a complex consisting of Toll-like receptor 4 (TLR4) and MD-2 through two phosphate groups of its lipid A moiety (Kim et al., 2007), and induces innate immune responses by activating two distinct pathways, namely NF-kB (through MyD88-dependent and independent pathways) and IRF-3 (through TRIF/TRAM). Although the specifics of LPS binding do not seem to be conserved between mammals and teleosts (Sullivan et al., 2009), this MAMP elicits similar pro-inflammatory responses through a Myd88-dependent mechanism in zebrafish (Bates et al., 2007) as in mammals.

Alps have been shown to remove the lipid A phosphates of LPS at physiological pH levels (Poelstra et al., 1997a,b), thereby reducing its affinity for TLR4 and, correspondingly, its endotoxic properties. Our studies in zebrafish larvae (Bates et al., 2006, 2007) demonstrated the functional significance of Alpi’s LPS dephosphorylating activity in the intestine, and showed that this gene plays an integral role in modulating innate immune responses in the gut through a Myd88-dependent negative feedback loop. We found that LPS incubation as well as Gram-negative bacterium inoculation upregulated zebrafish Alpi, a process that required MyD88. We showed that Alpi functioned in the detoxification of LPS because treatment with the Alpi-specific inhibitor l-phenylalanine or *alpi.1*-specific morpholino rendered zebrafish more sensitive in an LPS killing assay, whereas fish were resistant to LPS pretreated with calf Alpi. Furthermore, zebrafish with reduced Alpi activity exhibited elevated levels of pro-inflammatory cytokines and intestinal neutrophil influx, both Myd88-dependent processes. However, when Alpi deficient zebrafish were derived germ-free, removing microbiota-associated LPS, no excess neutrophil influx was observed. Collectively these results show that zebrafish intestinal colonization by Gram-negative bacteria upregulates the host enzyme Alpi, which functions to reduce host inflammatory responses to resident microbiota.

The anti-inflammatory function of Alpi is supported by many other observations from mammalian systems. Cell culture studies showed that in IECs (i.e., HT-29, T84, and IEC-6) overexpressing Alpi, LPS-activated NF-κB nuclear translocation was significantly inhibited (Goldberg et al., 2008). At the whole animal level, administration of bovine Alpi proved to reduce local/systemic inflammation and improve tissue morphology in the mouse polymicrobial sepsis model induced by cecal ligation and puncture (Van Veen et al., 2005), in the rat liver ischemia–reperfusion model (Van Veen et al., 2006), in the murine chronic colitis model induced by dextran sulfate sodium (DSS; Tuin et al., 2009; Campbell et al., 2010; Ramasamy et al., 2011) or TNBS (Martinez-Moya et al., 2012), and in the neonatal necrotizing enterocolitis rat model (Rentea et al., 2012). In clinical trials in humans, exogenous Alpi exerted protective anti-inflammatory effects on patients after cardiopulmonary surgery (Kats et al., 2009), patients with moderate to severe ulcerative colitis (Lukas et al., 2010), and patients with severe sepsis or septic shock (Heemskerk et al., 2009; Pickkers et al., 2012). Collectively, these findings confirm the importance of Alpi as an innate immune regulator, locally and systemically. LPS-detoxification by Alpi is also confirmed in cells (Goldberg et al., 2008) and animals (Beumer et al., 2003). The anti-inflammatory role of Alps is not restricted to the intestinal type, since Alps from other sources (e.g., placental Alp) protected mice against *Escherichia coli*-induced sepsis (Verweij et al., 2004).

Alp’s role in lipid A dephosphorylation and modulation of LPS recognition appears to be an ancient function for this family of enzymes, as demonstrated by recent work in the Hawaiian bobtail squid *Euprymna scolopes* (Rader et al., 2012). The squid acquires its Gram-negative bacterial symbiont *Vibrio fischeri* from the environment at the juvenile stage and thereafter enters a lifelong partnership with the luminous marine microbe. Rader et al. characterized two *E. scolopes* Alps (EsAlps), which are closely related to other mollusk Alps. EsAlp is highly active at the lumina of crypt spaces where the bacteria reside. Interestingly, EsAlp activity remained at low levels before and during the lipid A-induced tissue regression at the initial animal-bacteria contact. This enzyme regulation at the early stage proved important for the formation of the symbiotic relationship as demonstrated by two observations: (i) that inhibition of Alp by levamisole compromised the normal colonization of the symbiont and (ii) that *V. fischeri* lipid A pretreated by Alp failed to cause early stage apoptosis that is necessary for persistent colonization of *V. fischeri*. After colonization, however, the squid continuously adjusts EsAlp activity in accordance with the diel rhythm of bacterial population density, i.e., high at dusk and low at dawn, a pattern the authors suggest is strategically governed to render the animal insensitive to lipid A signaling by Alp dephosphorylation of LPS, and therefore protect the animal from excessive inflammation and tissue damage. Taken together, the data from *E. scolopes* presented an elegant example of the conserved role of Alps in tuning host immune recognition of LPS.

All animals live in close associations with microbial communities. Most frequently, the vast majority of these microbes reside in the digestive tract, where they assist the host in the breakdown of ingested food. Gut microbiota are highly species-specific, based on host phylogeny, diet, and digestive tract morphology (Ley et al., 2008a,b). Further microbial community specialization occurs along the length of the gut. Considering the conserved role of Alps in host-bacteria interactions, we imagine that intestinal Alps have been under continuous selective pressure to accommodate changing host-microbe interactions. For example, evolution of host digestive tract physiologies, driven by dietary changes, could spur *Alpi* gene duplication and diversification to buffer host inflammatory responses during the acquisition and compartmentalization of novel bacterial communities that facilitate food digestion.

In this report, we characterized two new *alp* genes in zebrafish and investigated their evolutionary history through the lens of the animal *Alp* gene phylogenies. We find that unlike the *Alpl* clade, the other *Alp* genes, which are frequently intestinally expressed, have been dynamically lost and duplicated throughout animal lineages, consistent with dynamically changing host-microbe interactions. These results suggest that *Alp* gene evolution has played an important role in shaping innate immune response to the intestinal microbiota.

## Results

### The four zebrafish *alp* genes are distributed among three vertebrate *Alp* gene clades

**Figure 1:**
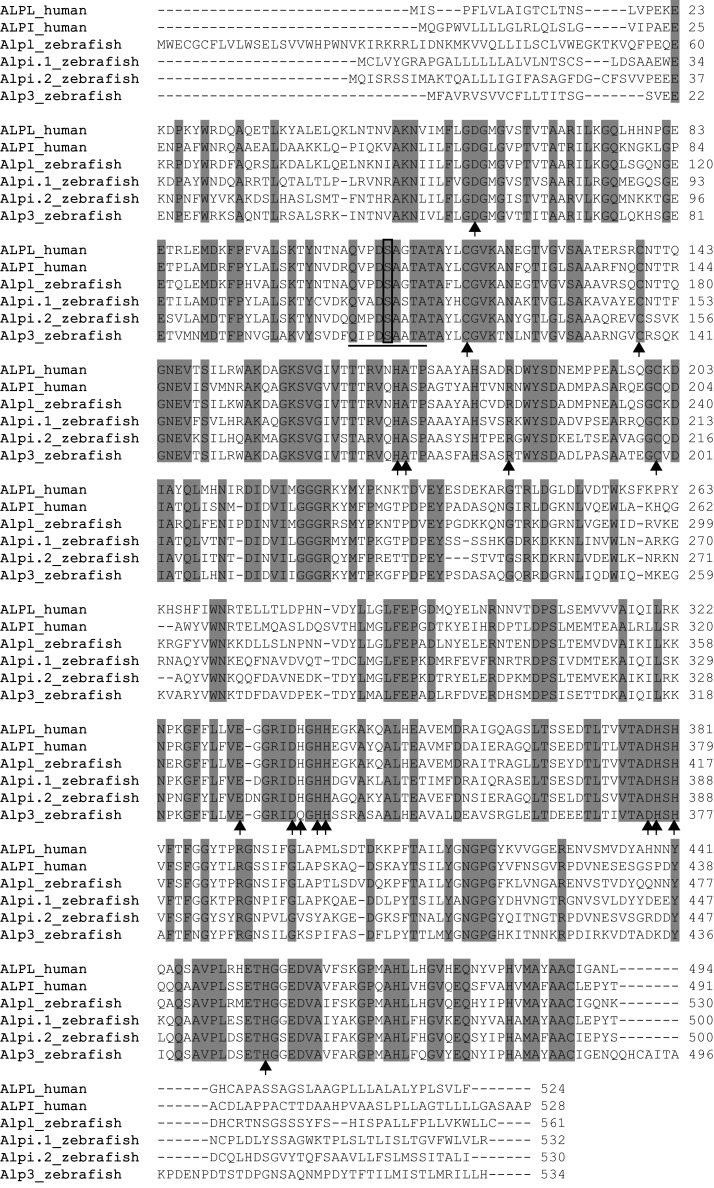
**ClustalW Sequence alignment of human and zebrafish Alps**. Identical amino acids are shaded gray, putative metal-binding sites are indicated by arrows, active sites are underlined, and the conserved serine required for enzyme activity is boxed. The proteins used in the alignment are human ALPL (ENSP00000363973) and ALPI (ENSP00000295463); zebrafish Alpl (ENSDARP00000117214), Alpi.1 (ENSDARP00000016216), Alpi.2 (ENSDARP00000070354), and Alp3 (ENSDARP00000019098).

We had previously characterized two zebrafish *alp* genes, which we called *alp* (accession number NM_201007.1) on chromosome 11 and *alpi* (accession number NM_001014353.1) on chromosome 22, and had shown that the former gene is ubiquitously expressed and the latter is intestinal-specific (Bates et al., 2007). BLAST searches against the subsequent refinement of the zebrafish genome sequence revealed two additional *alp*-related genes: *zgc:110409* (accession number NM_001025188.1) on chromosome 22, and the most recently described *alp*-related gene (accession number XM_003201677.1) on unassembled Scaffold Zv9 NA903 that Ensembl annotated as “*alpl*.” All four genes likely encode enzymatically active Alps based on their conserved Alp motifs. Protein sequence alignment against human ALPL and ALPI showed that active site residues and proposed metal-binding residues are invariant in the proteins encoded by the two human genes and four zebrafish genes (Figure 1).

**Figure 2:**
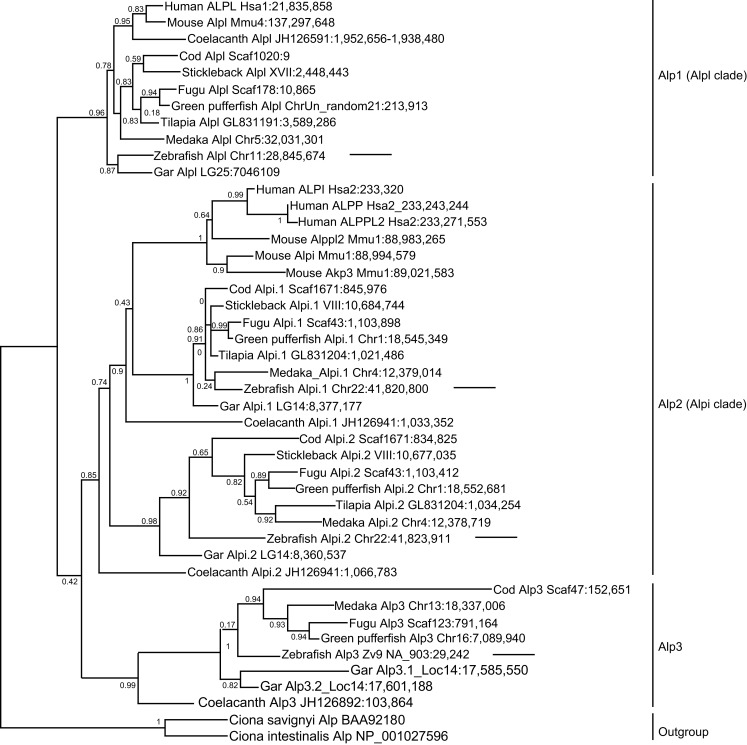
**Diversification of vertebrate Alp protein sequences**. Sequences are indicated on this maximum likelihood tree with the species name, gene name, and genomic location. For example, the taxon “Human ALPL Hsa1:21,835,858” indicates the start of the human *ALPL* gene encoding this protein on human chromosome 1 at nucleotide position 21,835,858 according to Ensembl Release 68 (July 2012) and Pre-Ensembl Release 66 (April 2012). The tree has three major clades [Alp1 (Alpl clade), Alp2 (Alpi clade), and Alp3]. Arrows point to the four zebrafish Alp genes.

To better understand the identity and origin of the four zebrafish *alp-*related genes, we investigated their evolutionary history. Phylogenetic analysis of vertebrate Alp protein sequences rooted on non-vertebrate chordate sequences revealed three distinct clades of *Alp* genes (Figure 2). The first of these clades, which we call Alp1, contains the human tissue non-specific gene *ALPL* and gene sequences from both lineages of bony vertebrates, the Sarcopterygii (lobe fin fish, including the basally diverging coelacanth and lungfish as well as tetrapods) and the Actinopterygii (ray fin fish, including the basally diverging gar as well as teleosts). The second clade, Alp2, contains the human intestinal-specific gene *ALPI*, the two human placental Alps, ALPP and ALPP2, as well as representative sequences from both lobefins (e.g., mammals) and ray fins (e.g., teleosts). The third clade, Alp3, contains genes exclusive to the fishes, including ray fins and the basally diverging lobe fin, the coelacanth.

Examination of the vertebrate *Alp* gene phylogeny suggests the following hypothesis for the origin of this gene family. A single *Alp* gene in a pre-vertebrate chordate duplicated to form four copies after the first and second vertebrate genome duplications (VGD1 and VGD2); we can call these genes *Alp1*, *Alp2*, *Alp3*, all of which persisted in some lineages, and *Alp4*, which was subsequently lost before the divergence of rayfins and lobefins. Rayfins and lobefins both retained *Alp1*, which became *Alpl*, and preserved *Alp2*, which experienced several lineage-specific tandem duplication events to become *Alpi-like* genes. *Alp3* persisted in rayfins and a basally diverging lobefin, the coelacanth, but was lost from crown lobefins, the tetrapods.

Analysis of the four zebrafish *alp* genes within this phylogeny revealed that (i) the chromosome 11 “*alp*” is a genuine ortholog of the human tissue non-specific gene *ALPL*; (ii) the chromosome 22 “*alpi*” and the neighboring “*zgc: 110409*” are tandem duplicates derived from the ancestral *alpi* gene and represent coorthologs of human *ALPI*; and (iii) the new Scaffold Zv9 NA903 *alp* annotated in Ensembl as “*alpl*” belongs to the Alp3 clade maintained in teleosts but lost in tetrapods. Based on these findings, we developed a new nomenclature for the four genes, i.e., the current “*alp*” is renamed *alpl*, “*alpi*” is *alpi.1*, “*zgc:110409*” becomes *alpi.2*, and “*alpl*” is *alp3*. We cloned and sequenced the complete coding DNA sequences of the four zebrafish *alp* genes and submitted to GenBank: *alpl* (accession number JX847415), *alpi.1* (accession number JX847416), *alpi.2* (accession number JX847417), and *alp3* (accession number JX847418).

### The vertebrate *Alpl* and *Alpi* genes arose during the vertebrate genome duplications

**Figure 3:**
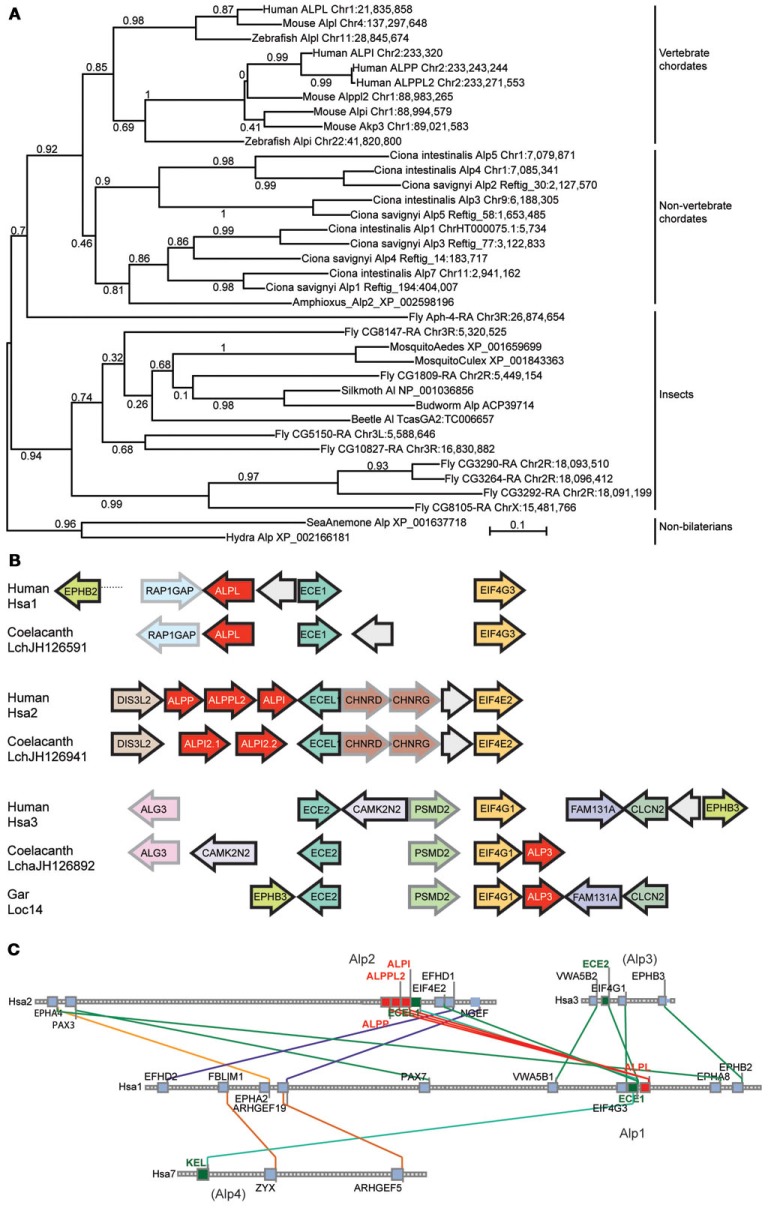
**Invertebrate Alp protein sequences and conserved syntenies**. **(A)** *Alp* genes amplified independently in vertebrate chordate and non-vertebrate chordate lineages, and in deuterostomes (including vertebrates) and protostomes (including insects). **(B)** Conserved syntenies among paralogons including human, coelacanth, and gar *Alp* genes. *Alp* genes in red, and paralogs of other gene families, such as *Ece1*, *Ecel1*, and *Ece2* genes and *Eif4* genes shown in unifying colors. Arrows represent direction of transcription. **(C)** Conserved syntenies in four paralogons in the human genome, only two of which have *ALP* genes. The figure shows four human chromosomes, Hsa1, Hsa2, Hsa3, and Hsa7, with lines between chromosomes connecting paralogs. Each square represents a gene; small gray squares from which no lines extend are genes without paralogs in the regions shown.

At least two alternative models can explain the origin of multiple Alp paralogs in vertebrates. Under one hypothesis, the *Alpi* and *Alpl* genes arose from gene duplication in pre-vertebrate ancestors and were inherited by the vertebrates, but under an alternative hypothesis, vertebrate *Alp* paralogs arose as ohnologs (paralogs derived from genome duplication) in the two rounds of whole genome duplication VGD1 and VGD2 that occurred at the base of the vertebrate radiation (Dehal and Boore, 2005). These two hypotheses and more complicated alternatives can be winnowed down by examining phylogenetic trees that include both invertebrate and vertebrate taxa. Figure 3A shows that, while non-vertebrate chordates (sea squirts *Ciona intestinalis* and *Ciona savignyi* and amphioxus) have multiple *Alp* genes, these diverge basal to the vertebrates, as would be expected if the vertebrate *Alp* genes arose after the divergence of vertebrates from non-vertebrate chordates. Likewise, except for one fly (*Drosophila melanogaster*) sequence, *Alp* genes of insects diverge basal to the chordates, while the non-bilaterian Cnidarian sequences (the sea anemone *Nematostella vectensis* and the hydra *Hydra magnipapillata*) root the tree. The preponderance of evidence leads to the conclusion that the *Alp* paralogs arose in the vertebrate genome duplication events.

Analysis of conserved syntenies comparing the human genome to the genomes of coelacanth and gar supports this model for *Alp* gene history. Located near *ALP3* in the coelacanth genome are the genes *EIF4G1* and *ECE2*, which are paralogs of genes located near the human *ALPL* and *ALPI* genes, namely *EIF4G3* – *ECE1* and *EIF4E2* – *ECEL1*, respectively (Figure 3B). At least five genes immediately adjacent to coelacanth *ALP3* are adjacent in the human genome, although inversions have altered gene order. These three paralogons containing *ALP* – *ECE* – *EIF4* genes likely resulted from two rounds of duplication, most parsimoniously explained as happening in the first and second vertebrate genome duplication events VGD1 and VGD2 (Dehal and Boore, 2005). The Synteny Database (Catchen et al., 2009) identifies four chromosome segments containing *ECE* paralogs, two with *ALP* gene neighbors and two without, including regions on Hsa3 and Hsa7 (Figure 3C). These data are consistent with the hypothesis that an ancestral chordate chromosome segment contained *ALP*, *ECE*, and *EIF4* genes became four paralogons after VGD2, followed in the human lineage by the loss of two of the *ALP* paralogs from the chromosome segments that eventually became the relevant part of Hsa3 and Hsa7 and the diversification of the eventual human chromosome 1 (Hsa1) and Hsa2 genes as the *ALPI-like* (*Alp2*) and *ALPL-like* (*Alp1*) genes of today’s vertebrates.

### Three of the zebrafish *alp* genes show enriched expression in the intestine

**Figure 4:**
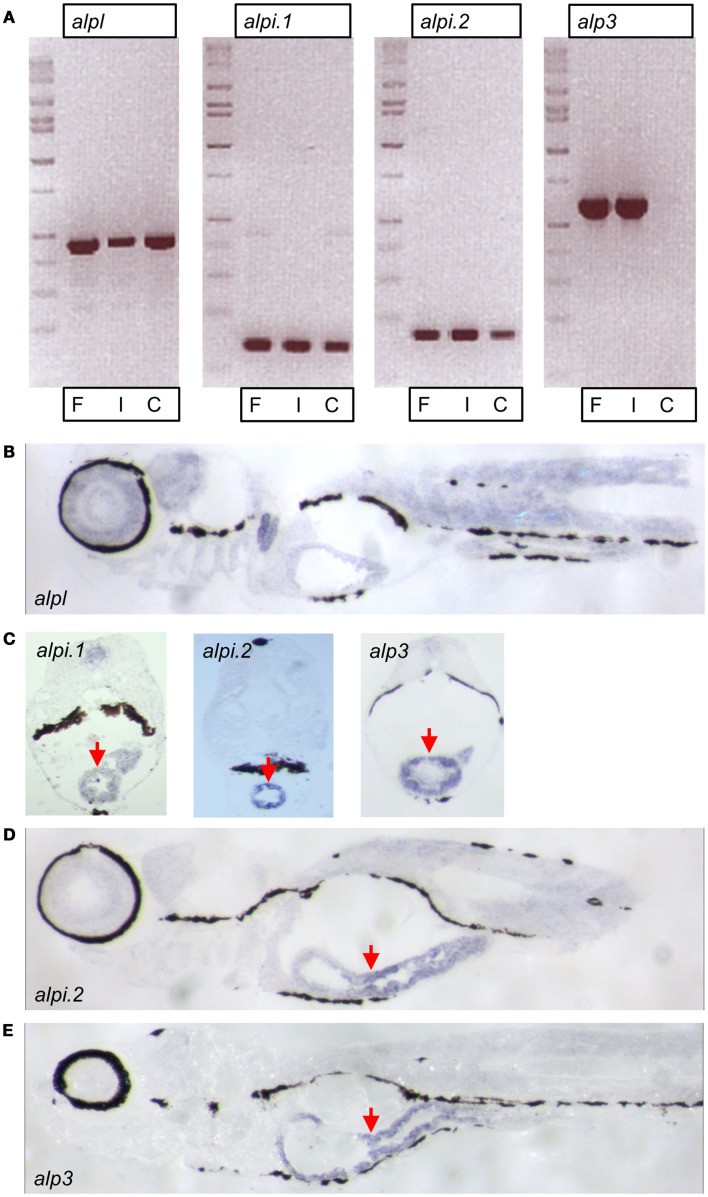
**Expression of zebrafish *alp* genes**. **(A)** Semi-quantitative reverse transcription PCR analysis of *alp* gene transcript levels at 7 days post fertilization (dpf) in whole fish (F), dissected intestines (I), and carcasses with intestine removed (C). *In situ* hybridization of 7 dpf larval sagittal **(B,D,E)** and transverse **(C)** sections with probes to *alpl*, *alpi.1*, *alpi.2*, and *alp3* as indicated. The hybridization is visible in blue. Arrows point to the intestinal epithelium.

We further explored the four zebrafish *alp* genes by investigating their tissue expression patterns. We used semi-quantitative reverse transcription PCR to estimate the abundance of the transcripts in intestinal tissue (“I”) versus the rest of the body (referred to as carcass, “C”). Transcript levels of *alpl* were abundant in the carcass as well as the intestine (Figure 4A). In contrast, transcripts of the other three genes were enriched (*alpi.1* and *alpi.2*) or exclusively expressed (*alp3*) in intestinal tissue (Figure 4A). We next performed *in situ* hybridization with gene-specific RNA probes to further examine the expression patterns of the four *alp* transcripts. Consistent with our previous analysis (Bates et al., 2007), we observed that *alpl* was diffusely expressed in many tissues (Figure 4B). Also, as we showed previously (Bates et al., 2007), a*lpi.1* was highly expressed in the intestine (Figure 4C). We also observed high levels of intestinal-specific expression of *alpi.2* (Figures 4C,D), indicating that the tissue-specific expression of this *alpi* coortholog has been maintained. Finally, we observed intestinal-specific expression of the teleost-specific *alp3* gene (Figures 4C,E), suggesting that intestinal-specific expression of *alp* genes is an ancestral trait possessed by the single *Alp* gene found in non-vertebrate chordates before the VGD1 and VGD2 events about 650 million years ago (Hedges et al., 2006; Braasch and Postlethwait, 2012).

### Mammalian *Alpi* genes have undergone rapid and dynamic evolution

**Figure 5:**
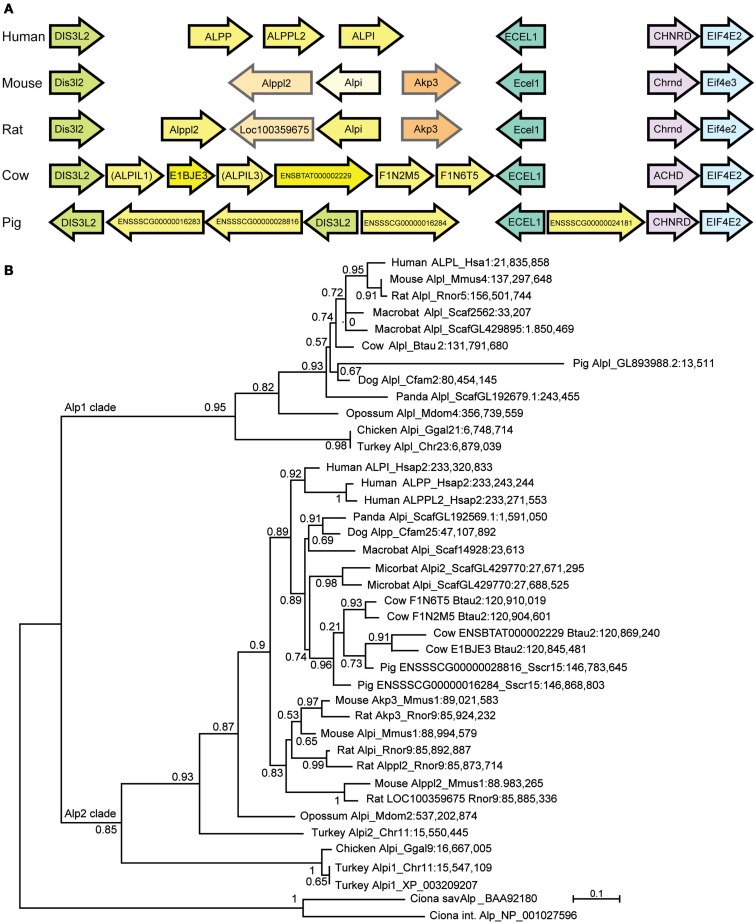
**The diversification of mammalian Alp protein sequences**. **(A)** The *Alpi-*related locus in five mammals showing *Alp* genes in yellow. Orthologs between mouse and rat shown in similar shades. **(B)** Maximum likelihood tree showing mammalian and bird *Alp* genes. In general, although the *Alpi* (*Alp2*) locus is orthologous and the flanking genes are orthologs among the various mammals, the tree shows that the *Alpi-*related genes themselves are usually not one-to-one orthologs.

Our phylogenetic analysis of the Alp gene family (Figure 1) suggested a dynamic evolutionary history of clades containing the intestinal-specific zebrafish *alp* genes. We examined this further through a phylogenetic analysis of mammalian *Alp* genes. All mammals examined in this study had a single *Alp* gene at the *Alpl-like* locus, which was surrounded by genes that were orthologs in all species with locally well-assembled genomes, showing that this region of the genome has been well-conserved among mammals. The genomic situation at the *Alpi-like* locus, however, differed greatly among taxa, with several species having multiple *Alpi-*related genes (Figure 5A).

Phylogenetic analysis rooted on bird and non-vertebrate chordate Alp sequences showed that many of these Alpi-like genes have arisen very recently (Figure 5B). For example, none of the three human genes (*ALPI*, *ALPP*, and *ALPP2*) is a unique ortholog of any non-primate *Alpi-like* gene despite names in common use.

The rodents mouse and rat also show shared and independent *Alpi-like* gene duplications (Figure 5B). The mouse *Alpi-like* locus contains three genes called *Alpi*, *Alppl2*, and *Akp3*. Although the human genome has genes called *ALPI* and *ALPPL2*, the mouse and human genes are not one-to-one orthologs according to phylogenetic analysis (see also Figure 3A). If this were the case, then the human and mouse *ALPI/Alpi* genes would group together in the tree and the *ALPPLP/Appl2* genes would group together; instead, the tree clearly shows that the human and mouse lineages diverged long before *ALPI* diverged from *ALPP* and *ALPPL2* and before *Alpi* diverged from *Alppl2* and *Akp3*.

The phylogenetic tree suggests that mouse and rat genomes contain at least two pairs of orthologous *Alpi-like* genes called *Akp3/Akp3* and *Alppl2/LOC100359675*, respectively (Figure 5B). In addition, the tree shows that rat has two genes derived from a recent duplication called *Alpi* and *Alppl2*. The tree shows with strong support that rat Alpi and Alppl2 arose in a tandem gene duplication event and that the rat Alpi sequence is more closely related to the rat Alppl2 sequence than it is to the mouse Alpi sequence, despite the names. In addition, although genes flanking these mammal’s *Alpi-like* locus are orthologs (Figure 5A), gene orientations are consistent with non-orthology or a gene-specific inversion event for the rodent genes called *Alpi*.

**Figure 6:**
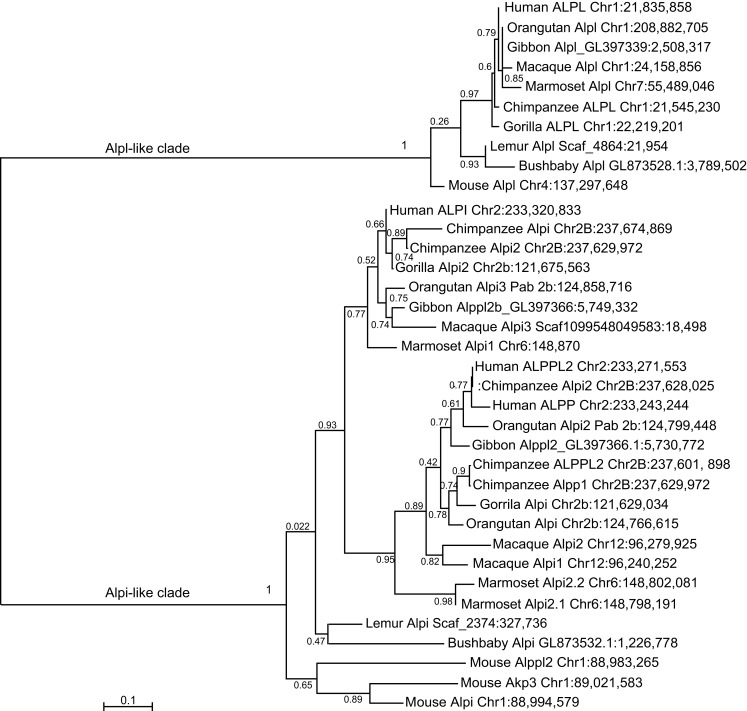
**Maximum likelihood tree of primate *Alp* sequences**. After the divergence of the Strepsirrhini (lemur and bushbaby) lineage from the Haplorrhini lineages, a tandem gene duplication produced an *Alpi-*like gene and an *Alpp*-like gene with further duplications within some of the Haplorrhini sub-lineages.

Similar findings come from a detailed phylogenetic analysis of the primate *Alp* genes (Figure 6). Although each primate had a single *Alpl* gene, the Alpi-like clade displayed several cases of independent tandem duplication events. The three *Alpi*-related genes of mouse formed a strong outgroup for the primate *Alpi* genes, suggesting that these three murine genes duplicated independently from the primate genes, confirming that none is a unique ortholog of any of the human paralogs. The single *Alpi* genes in the two Strepsirrhini species – lemur and bushbaby – grouped together at the base of the primate Alpi clade, which would be expected if ancestral primates had a single *Alpi* gene (Figure 6). A tandem duplication after the divergence of Strepsirrhini and Haplorrhini produced an Alpi clade and an Alpp clade; subsequently, after the divergence of the human and chimpanzee lineage about 6 million years ago, the human lineage experienced a tandem duplication in the ALPP clade and the chimpanzee lineage had independent duplications in the ALPP lineage, evidence of a dynamic diversification of ALP-related sequences in our recent history.

### Variable copy numbers of *Alpi* genes among mammals

**Figure 7:**
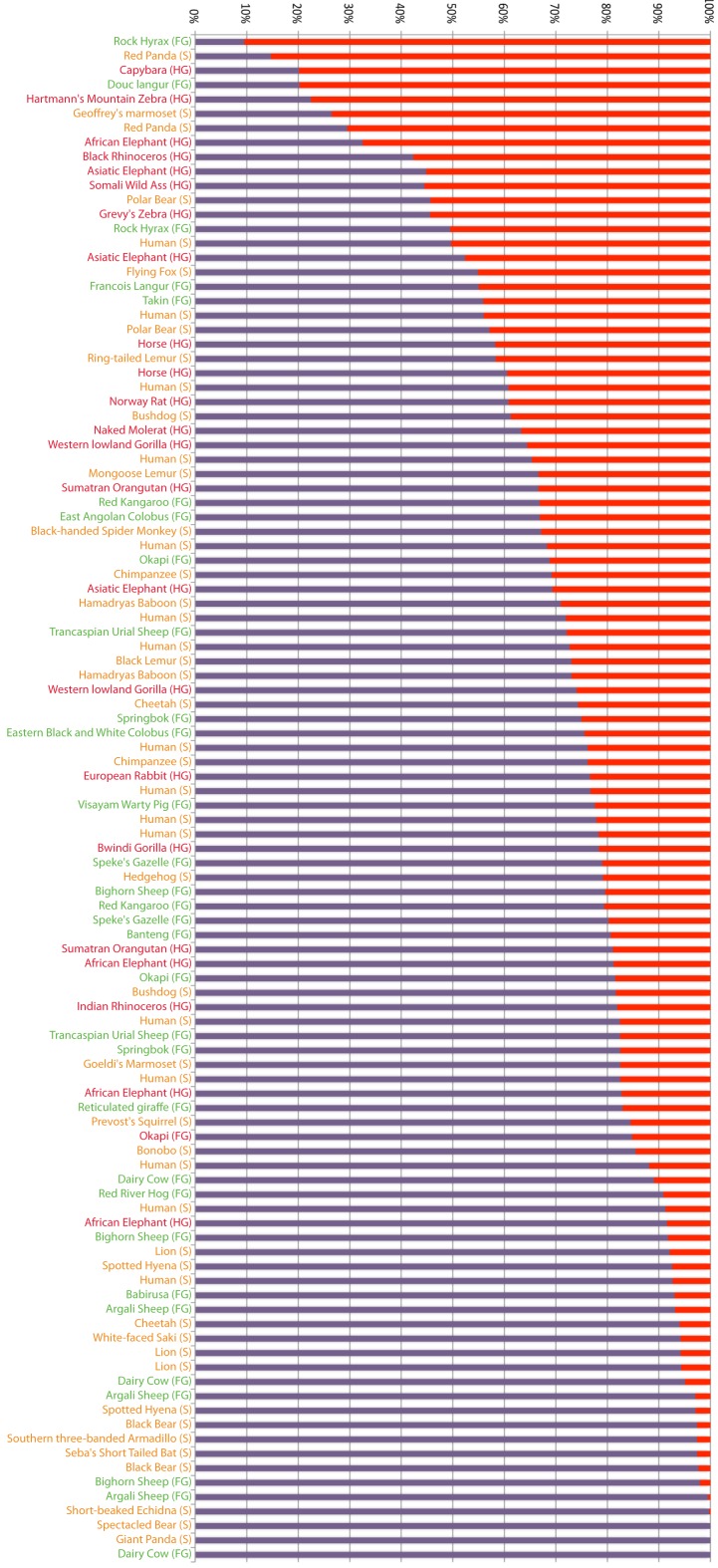
**Proportional representation of Gram-negative phyla in fecal samples from different mammals**. Bacterial composition of mammalian fecal samples, as previously reported (Ley et al., 2008a), are represented as the proportion of phyla composed of Gram-negative (red) and Gram-positive (purple) bacteria. Host animals’ gut morphologies are indicated and color coded (FG, foregut fermenter in green; S, simple in yellow; HG, hindgut fermenter in red).

We speculate that the rapid gains and losses of *Alpi* genes manifest in vertebrate lineages are driven by dynamic changes in host-microbe associations. In particular, if Alps play a conserved function in detoxifying LPS, then requirements for this gene could change rapidly with changing proportions of Gram-negative bacterial associates, possibly driven by adaptations to different diets that require different microbial physiologies for their metabolism. To explore this hypothesis, we determined the representation of Gram-negative phyla present in a published dataset of fecal samples from 60 mammalian species (Ley et al., 2008a; Figure 7). These samples contained an enormous range of proportional representation of Gram-negative phyla from 90% (rock hyrax) to 0% (cow). Even within one host species (humans), proportions ranged from 50 to 9%, emphasizing the variable nature of gut-associated microbial communities. We found no significant correlation between the proportional representation of Gram-negative bacteria and gut physiologies. We note, however, that fecal sampling is unlikely to capture the microbial composition of specialized gut compartments. For example, whereas the cow fecal sample from this study contained no Gram-negative phyla, surveys of cow rumen content typically find a high proportion of Gram-negative species (Jami and Mizrahi, 2012).

The limited number and sometimes low quality of whole genome sequences from mammalian species precluded us from performing a systematic correlation between *Alpi* gene copy number and gut morphology, but we noted some interesting trends. Although many of the mammals have multiple *Alpi-like* genes, three members of the order Carnivora that have simple gut morphologies – the giant panda, domestic dog, and domestic cat – all have only one *Alpi* gene (despite the fact that the panda is an herbivore). In contrast, Artiodactyls, which have more complex digestive systems, showed more complex *Alpi-like* genomics. The cow has six *Alpi*-related genes (called here *Alpi-like* *1–6*), only four of which (*Alpil2*;*E1BJE3*, *Alpil4;ENSBTAT000002229*, *Alpil5;F1N2M5*, and *Alpil6*;*F1N6T5*) are annotated in Ensemble. These four annotated genes are arranged in neighboring pairs oriented in the same direction (Figure 5A), with Alpil5 and Alpil6 falling as sisters in the tree and as neighbors in the genome and Alpil4 and Alpil5 also diverging as sisters and neighbors, suggesting tandem duplication events (Figure 5B). The pig has about three *Alpi-like* genes, although only two of them are well-assembled in Ensemble. One of the pig genes (*Alpil2*; *ENSSSCG00000028816*) appears to be coorthologous to at least two of the cow genes (*Alpi2* and *4*), suggesting that tandem gene duplications occurred in the Artiodactyl lineage before the divergence of swine and bovine lineages. The tree suggests the loss of ancestral Artiodactyl genes in the pig lineage and the tree and gene orientations indicate that independent duplications occurred at least in the cow lineage as well as local inversions during lineage divergence. An open and interesting question is whether different bovine *Alpi-like* genes are expressed in specific portions of the cow’s complex digestive system and are adapted to the different microbial contents of each compartment.

## Discussion

**Figure 8:**
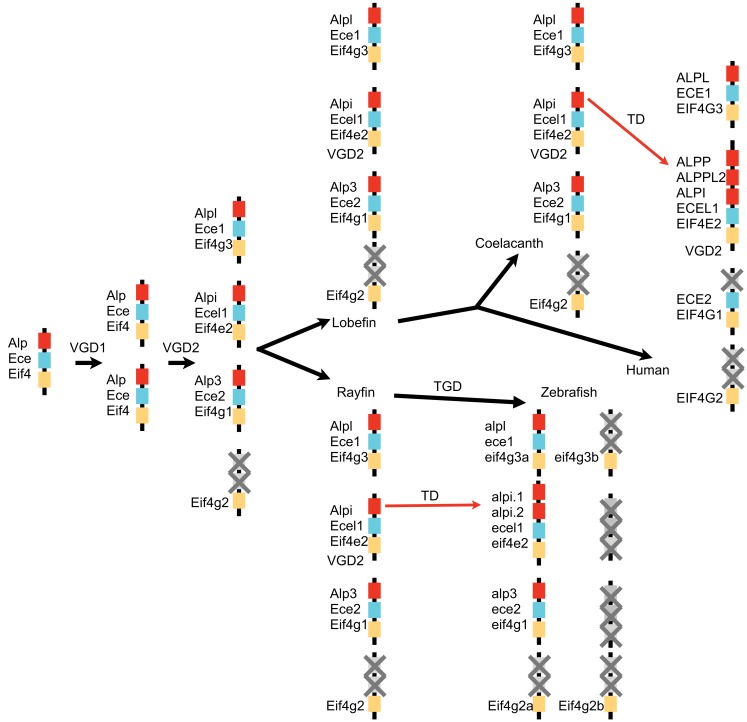
**A model for the history of *Alp* genes in vertebrates**. A chromosome segment in a non-vertebrate chordate contained *Ece*, *Alp*, and *Eif4* genes and duplicated twice in the vertebrate genome duplication events (VGD1 and VGD2) to make four paralogons, one of which lost its *Alp* gene. After the divergence of rayfin and lobefin bony fish lineages, the teleost genome duplication event (TGD) did not result in further extant *Alp* genes, but tandem duplications (TD) in the *Alpi* locus provided a number of separate *Alp* genes.

Our investigation into the phylogenetic relationship between *Alp* genes from zebrafish and other vertebrates reveals a remarkable evolutionary history for this gene family. As summarized in Figure 8, our analysis supports the model that the vertebrate *Alp* genes arose from a single *Alp* gene in ancestral chordates that duplicated during two rounds of genome duplication events (VGD1 and VGD2) that preceded the diversification of extant vertebrates after the divergence of vertebrates from non-vertebrate chordates and initially yielded four genes. One of these genes became the modern *Alpl*. This gene was faithfully maintained as a single copy throughout the vertebrates, with the coorthologous gene that would have been generated during a third round of genome duplication, the teleost whole genome duplication (TGD), having been lost. Indeed none of the three predicted coorthologous Alp genes are found within the teleost genomes we examined, but this could be due to chance; because about 75% of zebrafish coorthologues have been lost, the likelihood of all three Alp coorthologues being lost by chance is 42%, clearly a frequent occurrence for any three genes taken at random. The predicted fourth *Alp* generated during the two vertebrate genome duplications was lost before the divergence of lobefin and rayfin bony vertebrates. *Alp3* persisted in the rayfin lineage where it underwent tandem duplication in the gar and can be found in the basally diverging lobefin, the coelacanth, but was lost from crown group lobefins, the tetrapods. The evolutionary history of *Alp2* (*Alpi-like*) is the most dynamic. Examination of phylogenies of fishes, mammals, and primates reveals that this gene has undergone multiple independent losses and tandem duplications in its history. The role of gene conversion in homogenizing sequences after tandem duplications is as yet unstudied.

We speculate that the basis for the dramatically different evolutionary histories of the tissue non-specific *Alpl* and intestinal *Alpi* genes, which encode enzymes with well-conserved catalytic activities, lies in their different patterns of tissue expression. The ubiquitously expressed *Alpl* plays an important function in bone mineralization in mammals (Golub and Boesze-Battaglia, 2007). In humans, over 261 different mutations in *ALPL* have been linked to hypophosphatasia and skeletal abnormalities, of which 75% are missense mutations^1^. Several of these mutations result in dominant inheritance of the disease (Mornet et al., 2011), suggesting that modest changes in Alp function at sites of bone mineralization can lead to deleterious phenotypes, possibly restricting the evolution of copy number variation of this gene once it became dedicated to this function.

The frequent gains and losses of the intestinally expressed *Alp* genes across vertebrate lineages indicate much greater plasticity in the requirements for the enzyme encoded at this locus. The rapid evolution of gene copy number in the *Alpi* gene clade is especially striking among the mammals, which have undergone dramatic changes in their gut morphologies that accommodate different microbial fermentation strategies during adaptations to different diets, with innovations such as foregut fermentation arising independently multiple times in the mammalian tree (Stevens and Hume, 2004). Possibly the loss of *Alp3* prior to the divergence of the tetrapods put extra pressure on *Alpi* to accommodate changing requirements for Alp activity in the intestine. We hypothesize that requirements for Alpi function in detoxification of luminal LPS from Gram-negative gut bacteria changed dramatically with alterations in associated microbial communities, driven by dietary changes. Additionally, dietary changes may have altered the selective pressures on Alpi’s lipid absorption function. *Alpi* gene expansion may have been a way to tune the levels and spatial distribution of this enzyme within the gut and to allow the evolution of different gut compartments adapted to different bacterial communities or other gut functions. For example, one of the three mouse *Alpi-like* genes, *Akp3*, is restricted in its expression to the proximal duodenum (Narisawa et al., 2007). It would be interesting to determine whether the six *Alpi* genes in the cow are restricted to different regions of the elaborate gastrointestinal tract anatomy of this foregut fermenter. The gut is also a major site of infections, and the capacity of intestinal Alp to detoxify LPS is likely to be important for modulating immune responses to pathogens as well as resident beneficial bacteria. Epidemics of infections with Gram-negative enteric pathogens may have been additional driving forces that shaped the rapid evolution of the intestinal *Alp* genes.

In summary, the stark contrast between the evolutionary history of the vertebrate *Alpl* and *Alpi* clades suggests that these genes diversified in the functions they perform in organisms. We propose that the highly dynamic pattern of gene evolution of the *Alpi* clade is indicative of a gene family that serves important functions in mediating host-microbe interactions, which can change dramatically over short periods of time and impose strong selective pressures on animals.

## Materials and Methods

### Use of vertebrate animals

All zebrafish experiments were performed using protocols approved by the University of Oregon Institutional Care and Use Committee, and following standard protocols (Westerfield, 2007).

### Sanger sequencing of zebrafish Alp gene coding sequences

Fresh RNA was extracted from 7 days post fertilization (dpf) zebrafish larvae (illustra RNAspin mini kit, GE Healthcare). Total cDNA was synthesized using RNA as template (SuperScript III reverse transcription kit, Life technologies). The coding sequences of Alp genes were amplified from cDNA in PCR (Phusion DNA polymerase, Thermo Scientific). Primers used in PCR included alplF: 5′-ATGTGGGAATGTGGATGCTTTCTTG-3′, alplR: 5′-TCAGCAAAGCAGCCATTTGACC-3′; alpi.1F: 5′-ATGTGTTTGGTTTACGGTCGGGC-3′, alpi.1R: 5′-TCATCTCAAAACAAGCCAAAACACG-3′; alpi.2F: 5′-ATGGCCAAAACACAAGCCCTG-3′, alpi.2R: 5′-CTAAATAAGAGCAGTAATGGAGGACATCAG-3′; alp3F: 5′-ATGTTTGCTGTCCGTGTGTCC-3′, alp3R: 5′-TCAGTGCAGTAAAATCCTCATCAGTG-3′. PCR products were evaluated by DNA gel electrophoresis for purity and then extracted from gel. The purified PCR products were cloned into the pCR^®^ – Blunt II TOPO^®^ vector (ZeroBlunt^®^ TOPO^®^ PCR Cloning kit, Life Technologies). Clones carrying Alp gene coding sequences were sequenced (Sequetech). The complete coding sequences were assembled using the plasmid editor ApE^2^ and submitted to GenBank.

### PCR detection of zebrafish Alp gene transcription

Total cDNA was synthesized from fresh RNA at 7 dpf from whole fish, dissected intestines, or carcasses with guts removed. Gene-specific primers were used in PCR to test the presence of gene transcripts (alplF: 5′-TATTTCTTGGAGATGGGATGGGTG-3′, alplR: 5′-TTCAAAGAGTTGTCTGGCGATGTC-3′; alpi.1F: 5′-GCACCGCGCCAAAGCACAAG-3′, alpi.1R: 5′-CGGGCTTCGGAGGGCACATC-3′; alpi.2F: 5′-TGCGCTTTACGGAAACGGTCCA-3′, alpi.2R: 5′ – TGCGCCATCGGGCCTTTAGC-3′; alp3F: 5′-ATGTTTGCTGTCCGTGTGTCC-3′, alp3R: 5′-ACGA GAAACCGCCTCATCCAG-3′). Equal amounts of cDNA (200 ng/μl, NanoDrop 1000) were added as template in PCR.

### *In situ* hybridization

Zebrafish cDNA was obtained as describe above and used in PCR to amplify a unique fragment of *alpl* (alplF: TTCCAGAGCAAGAGAAGCGG; alplR: GTCTTAGAGAGGGCGACGTG), *alpi.1* (alpi.1F: CGACCGGGCGATTCAGAGAG; alpi.1R: TGGTGTACGGCTCAAGGCAC), *alpi.2* (alpi.2F: TCACTAACGGGACTCGACCT; alpi.2R: AGGCCATAGCGTGAGGAATG), and alp3 (alp3F: CAGGGTCATCACTCCAGTCG; alp3R: TCTGGACGCTTGTTGTTGGT). The purified PCR product was cloned into the pCR^®^ – Blunt II TOPO^®^ vector (ZeroBlunt^®^ TOPO^®^ PCR Cloning kit, Life Technologies) and validated by sequencing (Sequetech). The verified plasmid was used in PCR to add T7 RNA polymerase binding sites to the gene-specific fragment (alplF: TTCCAGAGCAAGAGAAGCGGC, alplT7-R: GTAATACGACTCACTATAGGGGTCTTAGAGAGGG; ali.1p-F: CGACCGGGCGATTCAGAGAG, alpi.1T7-R: GTAATACGACTCACTATAGGGTGGTGTACGGCTCAA; alpi.2F: TCACTAACGGGACTCGACCTGATGT, alpi.2T7-R: GTAATACGACTCACTATAGGGAGGCCATAGCGTGAG; alp3F: CAGGGTCATCACTCCAGTCGGGC, alp3T7-R: GTAATACGACTCACTATAGGGTCTGGACGCTTGTTG). The purified PCR product was used as template to synthesize the DIG labeled RNA probe (DIG RNA Labeling Mix (T7), Roche Applied Science).

Seven dpf zebrafish larvae were fixed in 4% PFA overnight at 4°C and then washed in 1×PBST. Rinsed larvae were embedded in 1.5% agar and cryo-cut into 16 μm thick sections. Sections were defrosted and air died overnight at room temperature. Diluted RNA probes were added on slides and incubated overnight at 70°C. After hybridization, slides were rinsed with the wash solution (1×SSC, 50% formamide, 0.1% Tween-20). Slides were then treated with the block solution (MABT, 2% blocking reagent, 20% heat inactivated sheep serum) for 3 h. Following that, first antibody solution (AP conjugated anti-DIG) was applied to slides and incubated overnight at 4°C. Slides were then washed in AP staining buffer (5 M NaCl, 1 M MgCl_2_, 1 M Tris pH 9.5, 20% Tween-20) and incubated with NBT/BCIP color regents in the dark at 37°C. The color developing process was stopped by washing slides in PBST. Slides were then rinsed in ddH_2_O, dehydrated in ethanol and eventually mounted in Permount. Images were taken under a Nikon TE2000 inverted microscope with a CoolSNAP camera (Photometrics).

### Genomic analyses

ALP-related sequences were obtained from sequenced genomes present in Ensembl^3^ and Pre-Ensembl^4^ by BLASTP searches using the human ALPI (ENSG00000163295) and ALPL (ENSG00000162551) sequences as query. Protein sequences for non-annotated genes in Ensembl were assembled using GeneMark^5^ (Lomsadze et al., 2005). Protein sequences were retrieved and subjected to phylogenetic analysis using MUSCLE for multiple alignment (Edgar, 2004), Gblocks to remove poorly aligned positions and divergent regions (Castresana, 2000), the PhyML maximum likelihood method for tree building (Guindon and Gascuel, 2003), and TreeDyn^6^ for tree rendering (Chevenet et al., 2006; Dereeper et al., 2008). Conserved synteny analyses were performed using the Synteny Database (Catchen et al., 2009). The sequences used in phylogeny study were provided in supplements.

## Conflict of Interest Statement

The authors declare that the research was conducted in the absence of any commercial or financial relationships that could be construed as a potential conflict of interest.

## Supplementary Material

The Supplementary Material for this article can be found online at http://www.frontiersin.org/Molecular_Innate_Immunity/10.3389/fimmu.2012.00314/abstract

Supplementary Data sheet S1 **Alp sequences used to generate Figure 2**.

Supplementary Data sheet S2 **Alp sequences used to generate Figure 3**.

Supplementary Data sheet S3 **Alp sequences used to generate Figure 5**.

Supplementary Data sheet S4 **Alp sequences used to generate Figure 6**.

## Footnotes

^1^http://www.sesep.uvsq.fr/03_hypo_mutations.php#legend

^2^http://biologylabs.utah.edu/jorgensen/wayned/ape/

^3^http://www.ensembl.org

^4^http://pre.ensembl.org

^5^http://exon.gatech.edu/eukhmm.cgi

^6^http://www.phylogeny.fr/

## Author Note

New sequences submitted to GenBank: zebrafish *alpl* complete CDS (accession number JX847415), zebrafish *alpi.1* complete CDS (accession number JX847416), zebrafish *alpi.2* complete CDS (accession number JX847417), and zebrafish *alp3* complete CDS (accession number JX847418).
