# Supplementary material for: Dynamic Evolution of the LPS-Detoxifying Enzyme Intestinal Alkaline Phosphatase in Zebrafish and Other Vertebrates
Source: Front Immunol. 2012 Oct 12;3:314. doi: 10.3389/fimmu.2012.00314 (PMC3469785; doi:10.3389/fimmu.2012.00314)
Supplement: Supplementary Data sheet S1 — Alp sequences used to generate Figure 2. [file 35467_Guillemin_DataSheet1.DOCX]

>Zebrafish_Alpi_Chr22:41,820,800_NP_001014375_ENSDARG00000015273]

MCLVYGRAPGALLLLLLALVLNTSCSLDSAAEWEKDPAYWNDQARRTLQTALTLPLRVNRAKNIILFVGDGMGVSTVSAARILRGQMEGQSGEETILAMDTFPYLALSKTYCVDKQVADSASTATAYHCGVKANAKTVGLSAKAVAYECNTTFGNEVFSVLHRAKAQGKSVGIVTTTRVQHASPAAAYAHSVSRKWYSDADVPSEARRQGCKDIATQLVTNTDIDVILGGGRMYMTPKGTPDPEYSSSSHKGDRKDKKNLINVWLNARKGRNAQYVWNKEQFNAVDVKTTDCLMGLFEPKDMRFEVFRNRTRDPSIVDMTEKAIQILSKNPKGFFLFVEGGRIDHGHHDGVAKLALTETIMFDRAIQRASELTSESDTLTVVTADHSHVFTFGGKTPRGNPIFGLAPKQAEDDLPYTSILYANGPGYDHVNGTRGNVSVLDYYDEEYKQQAAVPLESETHGGEDVAIYAKGPMAHLFHGVKEQNYVAHAMAYAACLEPYTNCPLDLYSSAGWKTPLSLTLISLTGVFWLVLR

>Zebrafish_Alpil_Chr22:41,823,911_NP_001020359_zgc:110409_ENSDARG00000053774]

MAKTQALLLIGIFASAGFDGCFSVVPEEEKNPNFWYVKAKDSLHASLSMTFNTHRAKNLILFLGDGMGISTVTAARVLKGQMNKKTGEESVLAMDTFPYLALSKTYNVDQQMPDSAATATAYLCGVKANYGTLGLSAAAQREVCSSVKGNEVKSILHQAKMAGKSVGIVSTARVQHASPAASYSHTPERGWYSDKELTSEAVAGGCQDIAVQLITNTDINVILGGGRQYMFPRETTDPEYSTVTGSRKDKRNLVDEWLKNRKNAQYVWNKQQFDAVNEDKTDYLMGLFEPKDTRYELERDPKMDPSLTEMVEKAIKILRKNPNGFYLFVEGGRIDHGHHAGQAKYALTEAVEFDNSIERAGQLTSELDTLSVVTADHSHVFSFGGYSYRGNPVLGVSYAKGEDGKSFTNALYGNGPGYQITNGTRPDVNESVSGRDDYLQQAAVPLDSETHGSEDVAIFAKGPMAHLFHGVQEQSYIPHAMAFAACIEPYSDCQLHDSGVYTQFSVAVLLFSLMSSITALI

>Zebrafish_Alp3_CABZ01061890_ENSDARG00000039048_Zv9_NA903:29,242_ENSDART00000024841_YeYang2]

FAVRVSVVCFLLTITSGSVEEENPEFWRKSAQNTLRSALSRKINTNVAKNIVLFLGDGGVTTITAARILKGQLQKHSGEETVNDTFPNVGLAKVYSVDFQIPDSAATATAYLCGVKTNLNTVGVSAAARNGVCRSQKGNEVTSILRWAKDAGKSVGIVTTTRVQHATPAASFAHSASRTWYSDADLPASAATEGCVDIATQLLHNIDIDVIIGGGRKYTPKGFPDPEYPSDASAQGQRRDGRNLIQDWIQKEGKVARYVWNKTDFDAVDPEKTDYLALFEPADLRFDVERDHSDPSISETTDKAIQILKKNPKGFFLLVEGGRIDQGHHSSRASAALHEAVALDEAVSRGLELTDEEETLTIVTADHSHAFTFNGYPFRGNSILGKSPIFASDFLPYTTLYGNGPGHKITNNKRPDIRKVDTADKDYIQQSAVPLDSETHGGEDVAVFARGPAHLFQGVYEQNYIPHAAYAACIGENQQHCAITAKPDENPDTSTDPGNSAQNPDYTFTILISTLMRILLH

>Zebrafish_alpl_ENSDARG00000015546_Chr11:28,845,674_YeYang1]

MWECGCFLVLWSELSVVWHPWNVKIRKRRLIDNKMKVVQLLILSCLVWEGKTKVQFPEQEKRPDYWRDFAQRSLKDALKLQELNKNIAKNIILFLGDGMGVPTVTAARILKGQLSGQNGEETQLEMDKFPHVALSKTYNTNAQVPDSAGTATAFLCGVKANEGTVGVSAAAVRSQCNTTQGNEVTSILKWAKDAGKSVGIVTTTRVNHATPSAAYAHCVDRDWYSDADMPNEALQSGCKDIARQLFENIPDINVIMGGGRRSMYPKNTPDVEYPGDKKQNGTRKDGRNLVGEWIDRVKEKRGYYVWNKKDLLSLNPNNVDYLLGLFEPADLNYELERNTENDPSLTEMVDVAIKILKKNERGFFLLVEGGRIDHGHHEGKAKQALHEAVEMDRAITRAGLLTSEYDTLTVVTADHSHVFSFGGYTPRGNSIFGLAPTLSDVDQKPFTAILYGNGPGFKLVNGARENVSTVDYQQNNYQAQSAVPLRMETHGGEDVAIFSKGPMAHLLHGVQEQHYIPHVMAYAACIGQNKDHCRTNSGSSSYFSHISPALLFPLLVKWLLC

>Stickleback_Alpi_VIII:10,684,744_ENSGACT00000011847_ENSGACG00000008915]

MPTAKCRANHHLLILLGPILLAVGRSAMESQAALYELEREPAYWDAQAKATLDAALKLRPREHQAKNLILFLGDGMGVSTVSAARILRGQMEGRSGEETMLAMDTFPYVALSKTYSVDKQVADSASTATAYHCGVKANAKTVGLSAKAVAYECNTTFGNEVFSVLRRAKAQGKSVGVVTTTRVQHASPAAAYAHSVSRSWYSDADLPSSAHQQGCVDIATQLVTNVDIDVILGGGRMYMTPKGTPDPEYPTSNSRKGDRKDKRNLIDVWLKAKPNKKSHYVWHRKEFDEINVKATDRLMGLFEPKDMRFEVFRNRTRDPSIVEMTEKAIQILSKNPKGYFLFVEGGRIDHGHHSGIAKLALTEAEMFDRAIQRAAQLTREHDTLTVVTADHSHVFTFGGNTPRGNPIFGLAPKNADDKMPFTSLLYANGPGYVHINGTRGNITMVDYYDEEYMQQAAVPLDVETHGGEDVAIYAKGPMAHLIHGVKEQNYVAHVMAYAACLEPYKNCPPHPHSHTSSGCATPTSSLLFGLLSFLWLLR

>Stickleback_Alpp_VIII:10,677,035_ENSGACT00000011789_ENSGACG00000008908]

MASIRELLFTGLFLLISVQWTVSISEEELHASYWNNKARQTLHTALNVQRNLKRAKNVILFLGDGMGLPTVTAARILKGQLAGKSGEETSLVMDTFPHLGLSKTYNVDQQMPDSAGTATAYLCGVKANYGTLGVTAATRRNDCSATYGNEVESVLSRAKKAGKSVGIVTTTRVQHASPAANYAHTANRDWYCDSDLTPEAIQNGCQDIAYQLVNNTEINVILGGGRRYMFSRTTKDPEYPTETGNRNDGQNLVEGWLKNKKKAKYVWNKVDFDAVKPSNTDFLMGLFEPKDCRYELERDPSMDPSLTEMTEKAIKILSKNPKGFYLFVEARGRIDHAHHGGTAKKALYEAVEFDRAIGRAAELTSELDTLTVVTADHSHVFAFGGHSARGNSVLGVSRSKANDNKHFTTAVYGNGPGYRNGTRPDMNETMSSDNNYLQQAPVPLDSETHGIEDVAIFAKGPMAHLFHGVHEQNYIAHVTAYAACLEPYENCELPDHAGSIHPSLLLLLMGLLFLSLCNV

>Stickleback_Alpl_ENSGACT00000006064_XVII:2,448,443_ENSGACG00000004517]

MKVIALLVVGTSALISGSFGKPQFPEQEKDPKFWNTWAQRTLKSALTLQKFNTNRAKNLVFFLGDGMGVPTVTAARILKGQLNGQSGEETQLEMDKFPFVSLAKTYNTNAQVPDSAGTATAYLCGVKANEGTVGVSAAATRTQCNTTRGNEVTSILRWASDTGMSVGIVTTTRVNHATPSAAYAHSVDRDWYSDNEMPAEALKNGCKDIARQLFDNIPNIAVILGGGRKYMFPKNTSDVEYPGVAKHSGTRNDGRNLVQEWIDGKKAKKGHYVWNKKQLLSLNPNNVEYLLGLFEPADMTYNLERNTDTDPSLPEMVEVAIKILRKNPKGFFLLVEGGRIDHGHHEGKAKQALHEAVEMDKAIGLAGLMTSVHDTLTVVTADHSHMFNFGGYTGRGNPMFGLAPMLSDIDQKPYTSIIYGNGPGYKLVNGGRENVSTVDYQENNYQAQAAVPLTMETHGGEDVAVFAKGPMAHLLHGVYEQNYIPHAMAYASCIGENREHCSGRSAALRPVLSSAAALLTVTRLLC

>Cod_Alpi_Scaf_1671:845,976_ENSGMOT00000001357_ENSGMOG00000001250]

ALYEREKEPAYWDAQARDTLVAALKLRPREHRAKNLILFLGDGMGVSTVTAARILKGQMEGKSGEETVLAMDSFPYLALSKTYSVDRQVADSASTATAYHCGVKANAKTVGLSARAVAYECNTTFGNEVYSVLRRAKAQGKAVGIVTTTRVQHASPAAAYAHSVSRSWYSDADLPASARRHGCVDIATQLVTNVDIDVILGGGRMYMTPKGTPDPEYPTSNSRKGDRKDRKNLIEQWLKAKPKKKSHYVWHKEQFDEINPKTTDRLMGLFEPKDMRFEVFRNNTRDPSIVEMTEKAIQILSKNPRGYFLFVEGGRIDHGHHDGVAKLALTEAVMFDRAIHRASRLTRESDTLTVVTADHSHVFTLGGNTPRGNSIFGLAAKKADDSMPFTSILYANGPGYVHVNGTRANITLVDYYDQEYMQQAAVPLDSETHGGEDVAIYARGPMAHLFHGVKEQNYVAHVMAYAACLPPYKDCPPHPHTQAASSGGRRTPASG

>Cod_Alpl_Scaf_1020:9_ENSGMOT00000021014_ENSGMOG00000019071]

QEKEPKFWNDWAQKTLDKALSLQTLNKNKAQNLILFLGDGMGVPTVTAARILKGQLRGQPGEEGQLEMDKFPFVALSKTYNTNAQVADSAGTATAYLCGVKANEGTVGVSAAAVRSQCNTTQGNEVTSILRWAKDAGKSIGIVTTTRVNHATPSAAYAHCVDRDWYSDNEMPADAVEAGCKDIARQLFENIPDIDVIMGGGRKYMYPKNTTDVEYPGQPKHSGTRKDGRNLVKEWVDRNTEKKGHYVWNKKDLLSLNPTKVDYLLGLFEPADLPYDLERNKETDPSLSEMVEVAIKILRRNPNGFYLLVEGGRIDHGHHEGKAKQALHEAVEMDRAIGRADLMTSTSDTLTVVTADHSHLFGFGGYTQRGNSIFGLAAMISDVDQKPFTAILYGNGPGYKLVNGARENVSTVDYQDNSYLAQAAVPLSSETHGGEDVAVFAKGPMAHLLHGVHEQNYIPHAMAYAACIGQN

>Cod_Alpp_Scaf_1671:834,825_ENSGMOT00000001326_ENSGMOG00000001225]

QELHPWYWNNGPGCYRRVPQNDPHRGQAKNLVLFLGDGMGVTTVTATRILKGQLAGFNGEETSLAMDTFPYLALSKTYNVNQQMPDSAGTATAYLCGVKANYGTLGVSAMIERGKCSGNADNDVVSVLHRAKMAGKSVGIVTTTRVQHASPGANYAHTPDRGWYSDIELPPDAVRNGCRDIAWQLIKNTEINVILGGGRQYMFSNITADPEYPTYFGERSDGKDLVKEWLQGKKNAKYVVNKEGLDAVDASKTDFLMGLFEPKDCQYENKRDTTTDPSLTQMMEKAIKILKRNPKGFYLFVEARGRIDHGHHDGNAQRALTEAVEFDRAIERAAQLTSEKDTLTVVTADHSHVFAFGGHSQRGNSVFGVSRELATDKKHFTTAVYGNGPGYQIKNGIRPDVNATVAAEPEYHQQAPVPKNSETHGIEDVPIFAKGPMAHLFHGVQEQSYIAHVLAYAACLPPYEKCDLEDNHSVCLHPSSVLVLMSLLLALVSC

>Cod_Alp3_ENSGMOG00000009300_Scaffold_47:152,651_ENSGMOT00000010217

QTYSVDFQIPDSAATATAYLCGVKTNLNTIGVSAAARNGVCRSQKGNEVTSILRWAKDAGQTFTHSSPHSGFCKYLDRLWLNNLLIGSTIFPHKNIHKLTWQSLSGRTINQIDHIIINGKWHAYSDHHLIAATINLKANKSNPKKPPAEDIAKLQYPKKNKEFVLELRNRFSMPEASSEILLWTFSRRSGSDRSDPHKHTNSHAPPLPLVTIRSVQAYPRYILIQSTGGRIDQAHHAGRGSMALHEAVALDRAVGKALELTNEEETLIVVTADHSHPLVINGYPFRGNSILGKSPLWASDMMPFTTLMYGNGPGNKMEGGKRPDIRKVDTKTSGYVQLAAVPLKSATHGGEDVVILARGPMAHLLQGVHEQSYIAHAMAYAACVGTDRRHCAARPAVAAARNQAGPYILQSVQWLLHIWF

>Human_ALPI_Hsa2:233,320_NP_001622_[Homosapiens]_ENSG00000163295]

MQGPWVLLLLGLRLQLSLGVIPAEEENPAFWNRQAAEALDAAKKLQPIQKVAKNLILFLGDGLGVPTVTATRILKGQKNGKLGPETPLAMDRFPYLALSKTYNVDRQVPDSAATATAYLCGVKANFQTIGLSAAARFNQCNTTRGNEVISVMNRAKQAGKSVGVVTTTRVQHASPAGTYAHTVNRNWYSDADMPASARQEGCQDIATQLISNMDIDVILGGGRKYMFPMGTPDPEYPADASQNGIRLDGKNLVQEWLAKHQGAWYVWNRTELMQASLDQSVTHLMGLFEPGDTKYEIHRDPTLDPSLMEMTEAALRLLSRNPRGFYLFVEGGRIDHGHHEGVAYQALTEAVMFDDAIERAGQLTSEEDTLTLVTADHSHVFSFGGYTLRGSSIFGLAPSKAQDSKAYTSILYGNGPGYVFNSGVRPDVNESESGSPDYQQQAAVPLSSETHGGEDVAVFARGPQAHLVHGVQEQSFVAHVMAFAACLEPYTACDLAPPACTTDAAHPVAASLPLLAGTLLLLGASAAP

>Human_ALPL_Hsa1:21,835,858_NP_000469_ENSG00000162551]

MISPFLVLAIGTCLTNSLVPEKEKDPKYWRDQAQETLKYALELQKLNTNVAKNVIMFLGDGMGVSTVTAARILKGQLHHNPGEETRLEMDKFPFVALSKTYNTNAQVPDSAGTATAYLCGVKANEGTVGVSAATERSRCNTTQGNEVTSILRWAKDAGKSVGIVTTTRVNHATPSAAYAHSADRDWYSDNEMPPEALSQGCKDIAYQLMHNIRDIDVIMGGGRKYMYPKNKTDVEYESDEKARGTRLDGLDLVDTWKSFKPRYKHSHFIWNRTELLTLDPHNVDYLLGLFEPGDMQYELNRNNVTDPSLSEMVVVAIQILRKNPKGFFLLVEGGRIDHGHHEGKAKQALHEAVEMDRAIGQAGSLTSSEDTLTVVTADHSHVFTFGGYTPRGNSIFGLAPMLSDTDKKPFTAILYGNGPGYKVVGGERENVSMVDYAHNNYQAQSAVPLRHETHGGEDVAVFSKGPMAHLLHGVHEQNYVPHVMAYAACIGANLGHCAPASSAGSLAAGPLLLALALYPLSVLF

>Human_ALPP_Hsa2:233,243,244_NP_001623_[Homosapiens]_ENST00000392027]

MLGPCMLLLLLLLGLRLQLSLGIIPVEEENPDFWNREAAEALGAAKKLQPAQTAAKNLIIFLGDGMGVSTVTAARILKGQKKDKLGPEIPLAMDRFPYVALSKTYNVDKHVPDSGATATAYLCGVKGNFQTIGLSAAARFNQCNTTRGNEVISVMNRAKKAGKSVGVVTTTRVQHASPAGTYAHTVNRNWYSDADVPASARQEGCQDIATQLISNMDIDVILGGGRKYMFRMGTPDPEYPDDYSQGGTRLDGKNLVQEWLAKRQGARYVWNRTELMQASLDPSVTHLMGLFEPGDMKYEIHRDSTLDPSLMEMTEAALRLLSRNPRGFFLFVEGGRIDHGHHESRAYRALTETIMFDDAIERAGQLTSEEDTLSLVTADHSHVFSFGGYPLRGSSIFGLAPGKARDRKAYTVLLYGNGPGYVLKDGARPDVTESESGSPEYRQQSAVPLDEETHAGEDVAVFARGPQAHLVHGVQEQTFIAHVMAFAACLEPYTACDLAPPAGTTDAAHPGRSVVPALLPLLAGTLLLLETATAP

>Human_ALPPL2_Hsa2:233,271,553_NP_112603_ENST00000295453_[Homosapiens]

MQGPWVLLLLGLRLQLSLGIIPVEEENPDFWNRQAAEALGAAKKLQPAQTAAKNLIIFLGDGMGVSTVTAARILKGQKKDKLGPETFLAMDRFPYVALSKTYSVDKHVPDSGATATAYLCGVKGNFQTIGLSAAARFNQCNTTRGNEVISVMNRAKKAGKSVGVVTTTRVQHASPAGAYAHTVNRNWYSDADVPASARQEGCQDIATQLISNMDIDVILGGGRKYMFPMGTPDPEYPDDYSQGGTRLDGKNLVQEWLAKHQGARYVWNRTELLQASLDPSVTHLMGLFEPGDMKYEIHRDSTLDPSLMEMTEAALLLLSRNPRGFFLFVEGGRIDHGHHESRAYRALTETIMFDDAIERAGQLTSEEDTLSLVTADHSHVFSFGGYPLRGSSIFGLAPGKARDRKAYTVLLYGNGPGYVLKDGARPDVTESESGSPEYRQQSAVPLDGETHAGEDVAVFARGPQAHLVHGVQEQTFIAHVMAFAACLEPYTACDLAPRAGTTDAAHPGPSVVPALLPLLAGTLLLLGTATAP

>Mouse_Alppl2_Mmu1:88,983,265_ENSMUST00000027455[Mouse

MWGACLLLLGLSLQVCPSVIPVEEENPAFWNRKAAEALDAAKKLKPIQTSAKNLVILMGDGMGVSTVTATRILKGQQQGHLGPETQLAMDRFPHMALSKTYNTDKQIPDSAGTGTAFLCGVKTNMKVIGLSAAARFNQCNTTWGNEVVSVMHRAKKAGKSVGVVTTTSVQHASPAGTYAHTVNRGWYSDAQMPASALQDGCKDISTQLISNMDIDVILGGGRKFMFPKGTPDQEYPTDTKQAGTRLDGRNLVQEWLAKHQGARYVWNRSELIQASLNRSVTHLMGLFEPNDMKYEIHRDPAQDPSLAEMTEVAVRMLSRNPKGFYLFVEGGRIDHGHHETVAYRALTEAVMFDSAVDKADKLTSEQDTMILVTADHSHVFSFGGYTQRGASIFGLAPFKAEDGKSFTSILYGNGPGYKLHNGARADVTEEESSNPTYQQQAAVPLSSETHSGEDVAIFARGPQAHLVHGVQEQNYIAHVMAFAACLEPYTDCGLASPAGQSSAVSPGYMSTLLCLLAGKMLMLMAAAEP

>Mouse_Alpi_Mmu1:88,994,579_ENSMUST00000113270[mouse

MQGDWVLLLFLGLRIHLSFGIIPAEEENPAFWNKKAAEALDAAKKLQPIQTSAKNLIIFLGDGMGVPTVTATRILKGQLEGHLGPETPLAMDLFPYMALSKTYNVDRQVPDSAGTATAYLCGVKANYKTIGLSAAARLDQCNTTFGNEVFSVMYRAKKAGKSVGVVTTTRVQHASPAGTYAHTVNRNWYSDAEMPASALQDGCKDIATQLISNMDIDVILGGGRKFMFPKGTPDPEYPSDSNQSGTRLDDQNLVQTWLSKHQGARYVWNRSELIQASQDPAVTHLMGLFEPTEMKYDANRNPSVDPSLAEMTEVAVRMLSRNPQGFYLFVEGGRIDQGHHAGTAYLALTEAVMFDSAIEKASQLTNEKDTLILITADHSHVFAFGGYTLRGTSIFGLAPLKALDDKSYTSILYGNGPGYELKSGNRPNVTEAQSVDPNYKQQAAVPLSSETHGGEDVAIFARGPQAHLVHGVQEQNYIAHVMAFAGCLEPYTDCGLAPPAGQSPVITPGQATTTNNAAGQATTTNNAAGQATVLLSLQLLVSMLLLVGTAMVVS

>Mouse_Akp3_Mmu1:89,021,583_ENSMUST00000044878[mouse

MQGTWVLLLLGLRLQLSLSVIPVEEENPAFWNKKAAEALDAAKKLQPIQTSAKNLIIFLGDGMGVPTVTATRILKGQLEGHLGPETPLAMDRFPYMALSKTYSVDRQVPDSASTATAYLCGVKTNYKTIGVSAAARFDQCNTTFGNEVFSVMYRAKKAGKSVGVVTTTRVQHASPSGTYVHTVNRNWYGDADMPASALREGCKDIATQLISNMDINVILGGGRKYMFPAGTPDPEYPNDANETGTRLDGRNLVQEWLSKHQGSQYVWNREQLIQKAQDPSVTYLMGLFEPVDTKFDIQRDPLMDPSLKDMTEAAVKVLSRNPKGFYLFVEGGRIDRGHHLGTAYLALTEAVMFDLAIERASQLTSERDTLTIVTADHSHVFSFGGYTLRGTSIFGLAPLNALDGKPYTSILYGNGPGYVGTGERPNVTAAESSGSSYRQQAAVPVKSETHGGEDVAIFARGPQAHLLHGVQEQNYIAHVMAFAGCLEPYTDCGLAPPADESQTTTTTRQTTITTTTTTTTTTTTPVHNSARSLGPATAPLALALLAGMLMLLLGAPAES

>Mouse_Alpl_Mmu4:137,297,648_ENSMUST00000030551

MISPFLVLAIGTCLTNSFVPEKERDPSYWRQQAQETLKNALKLQKLNTNVAKNVIMFLGDGMGVSTVTAARILKGQLHHNTGEETRLEMDKFPFVALSKTYNTNAQVPDSAGTATAYLCGVKANEGTVGVSAATERTRCNTTQGNEVTSILRWAKDAGKSVGIVTTTRVNHATPSAAYAHSADRDWYSDNEMPPEALSQGCKDIAYQLMHNIKDIDVIMGGGRKYMYPKNRTDVEYELDEKARGTRLDGLDLISIWKSFKPRHKHSHYVWNRTELLALDPSRVDYLLGLFEPGDMQYELNRNNLTDPSLSEMVEVALRILTKNLKGFFLLVEGGRIDHGHHEGKAKQALHEAVEMDQAIGKAGAMTSQKDTLTVVTADHSHVFTFGGYTPRGNSIFGLAPMVSDTDKKPFTAILYGNGPGYKVVDGERENVSMVDYAHNNYQAQSAVPLRHETHGGEDVAVFAKGPMAHLLHGVHEQNYIPHVMAYASCIGANLDHCAWAGSGSAPSPGALLLPLAVLSLRTLF

>Coelacanth_Alp_JH126591:1,952,656-1,938,480_ENSLACT00000019682_ENSLACG00000017187

MKIFLLLVSVQICLISAGFPACVEHEKDPWYWRGQAQKTLQKALSLQHLNTRVAKNIIMFLGDGMGVATVTAARILKGQLQSKSGEETQLEMDKFPYVALSKTYNTNAQVPDSAGTATAYLCGVKANEGTLGVTAATVRGQCNTTTGNEVTSILRWAKDAGKAVGIVTTTRVTHATPSAAYAHAANRDWFSDNEMPPEALQQGCKDIARQLVDNVPDIEVIMGGGRKYMAPQNTRDIEYPNEPKANGTRLDGRNLTEEWIERVKTENAQYVWNLRQLKQLNLKEVDFLLDFVKGLFEPHDLMYELDRNRETDPSLQDMVEVAIKILRKNPNGFFLLVEGGRIDHGHHEGKAKQALHEAVEMDKAIGIAGMMTSERDTLTVVTADHSHVFTFGGYTPRGNPLFGMAPMLSDVDNKPFTSILYGNGPGYKVINGERENVSNIDIHYNNYLAQSAVPLRQETHGGEDVAVFAKGPMAHLLHGVHEQNYIPHVMAYAACIGQNKEHCKTHYPLSCSASTVLATLSTLVLLLLF

>Coelacanth_Alpi_JH126941:1033352-1058969]

EKKPQFWYDKAKLSLETALNLKSLNYQAKNLILFLGDGMGVSTVTAARIYKGQRNGKLGEEEIMAMDTFPYVALSKTYCVDRQVPDSAATGTAYLCGVKTNYRVLGLSGSAVSSQCNTTYGHEVYSVLHRAKQAGRVQHASPAAAYAHIVNRNWYADSSMPKDAIQDGCKDIAYQLVHNTDFDVILGGGRMYMTPMGTPDPEYPWDQKQNGIRNDGVNLIQTWLDARKLGLFEPKDMKYELNRNITLDPSIVEMMEKAIGILSKNPKGFFLFVERGRIDHGHHDSKAKMALTETVLFEQAIQRAGELTSDIDTLSVVTADHSHVFSFGGKTYRGSNNLGVAPKNADDNLPYTSILYGNGPGFNIQNGTRPNITMTDIGDYTQQAAVPLDSETHAGEDVAIYAKGPMAYLFHGVQEQTYIAHAMAYAACIEPYMDC

>Coelacanth_ALPL2_JH126892:103,864_ENSLACT00000004503_ENSLACG00000003972]

LGFYLMCAVAAGIIPAKERDPNYWRQQAQGTLRNALNHQHNTNVAKNVVLFLGDGMGLATVTAARILKGQMKNKLGEETLLTMETFPHVALSKTYNIDLQVPDSAGAATAFLCGVKANSGTVGVSAAAFNGICTSQFGNEVTSILKWAKDTGKSVGVVTTTRVQHASPAAAYAHSVNRRWYTDSNVPAAAKKEGCRDIAYQLVFNTDIDVIMGGGRKYMTAKGTPDPEYPLDFRSRGTREDGLDLVTEWQRNKTGKVAHYVWNKQDFDAVNANTTDYLLALFEPSDMKYEQDRNPEKDPSIVEMTEKAIQILQRNPKGFFLFVEGGRIDQGHHAGKASQALHEAVMLDNAIQKAIELTSEEDTLTVVTADHSHAFIFGGQPLRGNPILGKSSLFATDFMPYTTLLYGNGPGFKISNDKRPDIQKVNTASKDYLQQAAVPLDSETHGGEDVAIFARGPMAHLFHGVQEQNYIAHVMAYAACIGQNLEHCAGQSSPAQTLTLLPLLLTLPPAS

>Coelacanth_Alpl_JH126941:1,066,783_ENSLACT00000026213_ENSLACG00000014466]

MKSLGSSGVLFCALFQLSIAIIPVQEIKPSFWNLKAKSSLEEALKLKQRHHRAKNLILFLGDGMGVPTVTAARILKGQLLSHFGEETVLTMDTFPYLALSKTYNVDHQVPDSAGTATAYLCGVKGNYGTIGLNAAAIRSNCTSSIGNHVTSILKRAKDVGKSVGIVTTTRVQHASPAGNYAHVAERNWYSDASMPTDALKDGCKDIAYQLLHNTDINVILGGGRKYMTPEGTKDPEYPEHPKENGTRKDGIDLIQNWLNSKKGAKYVWNKEQFDAVDVSTTNYLMGLFEPADMKYELNRNNSMDPSIVDMTEKAIRILSENPKGFYLFVEGGRIDHGHHSSKAKKALIEAVMFDHAIQRAGELTSEEETLTVVTADHSHTFTFGGYTERGNSIFGLAPAKANDRKHYTSILYGNGPGFAMQNGTRPAVNETISDDNEYKQQAAVPLDSETHGGEDVVIFSKGPMAHLFHGVQEQNYIPHVMAYAACIEPYSDCSFEAPASSHIPKASLLTLILALAVFVVNF

>Gar_Alpi1_LocLG14:8,377,253_ENSDARP00000095231_1

EKDPAYWNTQAKETLQAALKMKPISHRAKNIILFLGDGMGVSTVSATRILKGQMEGNSGEETVLTMDTFPHLALSKTYSVDMQVADSASTGTAYLCGVKANAKTVGVSAAAVAYKCNSTFGNEVYSVLHRAKAQGKSVGIVTTTRVQHASPAASYAHSVSRSWYSDADLSSSALRDGCKDLATQLVFNTDIDVILGGGRMYMTPKGTPDPEYPTSSSRRGKRKDGKNLIELWLFLLQSKNAQYVWHKEQFDAVDVNSTNCLMGLFEAKDMRFELYRNSTRDPSIIEMTEKAIQILSKNPKGFFLFVEDKYPLTFPHTAQ*GCILLY*SDLRGRIDHGHHDGIAKLALTEAVMFDRAIQRASQLTRDSDTLTVVTADHSHVFTFGGNTPRGNPIFGLVPEMAEDDLPYTSILYANGPGYLLENGTRANITVLPVDEEYMQQAAVPLDSETHGGEDVAIFAQGPMAHLFHGVKEQNYIAHVLAYAACLEPYTDCPPEPNSGSG

>Gar_Alpi2_LocLG14:8,360,649_ENSDARP00000070354_1

RQALEEALELKPNAGKAKNLILFLGDGMGVSTVTATRILKGQLEGKXXXXXXVCADFAACFPPS*TYNVDHQAPDSAGTATAYLCGVKANYGTVGLSAAARRYQCNTTKGNEVTSILHRARKAGKSVGIVTTTRVQHASPAASYAHSVDRNWYSDSDLPHDALAGGCADISFQLIHNSDINVILGGGRQYMTPNGTKDPEYSSASARGKRQDGINLIDKWSRLFQKAVYVWNKTEFDAVNENDTEYLMGLFEPKDTRYELDRNKTQDPSLTEMTEKAIKILRKNPKGFFLFVEDKGRIDHGHHDSRARYALTEAVEFDRAIERAAELTSKLDTLTVVTADHSHVFTIGGKAERGNSVFGLAPSNADDNKPYTTILYGNGPGYIMENGTRPAPNITNIQNKAYRQQSAVLLDSETHGAEDVAIFAKGPMAHLFHGVQEQSYIAHVMAFAACIEPYEACSMKD

>Gar_Alp1_LocLG14:17,585,550_ENSDARP00000019098_1

EERKPQFWFDLAQRRLQSRLSQRDSTSTARNLLLFIGDGMGLNTITATRILKGQLRGGTGEEFTLAMDTFPYTALSKTYCTDAQIADSTCAATALLCGVKTNKFTAGLSAGATYDRCNTSDGHHVTSILRWAKDAGKSVGFVTTVRVQHATLAPAYAHTPNRYWYSDANMPAEQRQQGCKDIAHQLVHNIPDIEVIMGGGRKYMTPAATPDPEYPSDSRSQGLRLDGVNLIQKWKDLKPGKNATYVWNKADLDRVDPDKTDYLLALFEPGDMSFELERNNTTDPSLPDMTDVALKILRKNPKGFFLLVEGGRIDQGHHASRASMALHEAVALDRAVARALEITNENDTLTLATADHSHAFSFNGYPFRGNPILGKSPLFGKDSLPFTTLMYGNGPGHKIVNNKRPDIRGVDTTSNAYVQQSAVPMDTETHGGEDVAVFARGPMAHLFHGVQEQNYIPHALAYAACLGENLGHC

>Gar_Alp2_LocLG14:17,601,188_ENSLACP00000004464_1

ADEEDPAFWRRQAQESLQAALSLHHNTNVAKNILLFLGDGMGITTLTAARILKGQLENRSGEETVMTMDTFPYVGLAKTYSVDFQIPDSASTATAYLCGVKSNLNTVGLSGAGRNGVCRSQKGNEVTSILRWAKDAGKSVGIVTTTRVQHATPAAAYAHSASRKWYADSDMPASAKEGGCRDIAHQLINNIDIDVIIGGGRKYMTPRGTKDPEYPYDLAAAGRRADGRDLIAEWQKSKAGKVAHYVWNKKDFDAIDSNTTDYLMALFEPGDLRFELERDPVKDPSVIETTEKAIQILQKNPKGFFLLVEGGRIDQGHHASRASMALHEAVALDRAVARALEITNENDTLTLATADHSHAFSFNGYPFRGNPILGKSPLFGKDSLPFTTLMYGNGPGHKIVNNKRPDIRGVDTTSNAYVQQSAVPMDTETHGGEDVAVFARGPMAHLFHGVQEQNYIPHALAYAACLGENLGHCLSPSSTTE

>Gar_Alpl_LocLG25:7,036_ENSDARP00000117214_1

EEEKNPRFWNDMAQKTLQKALSLQEHNKNIAKNIILFLGDGMGIPTVTAARILKGQMNGHSGEETELEMDKFPYVALSKTYNTNAQVPDSAGTATAYLCGVKANEGTVGVNAAAVRSQCNTTQGNEVSSILKWAKDAGKSVGIVTTTRINHATPSAAYAHCVDRDWYSDGEMPQDAIEGGCKDIARQLFENIPNIDVIMGGGRKYMYPKNTSDVEYPNERKHSGTRRDGRSLIEEWKNRTSSMNGHYVWNRRQLLSLDPNRVNYLLGLFEPGDMPYELERNKDTDPSLTEMVEIAIKILRRNPRGFYLLVEGGRIDHGHHEGKAKQALHEAVEMDKAIGRAGLLTSEEDTQTVVTADHSHVFTFGGYTHRGNPIFGLAPMMSDIDQKPFTSILYGNGPGYKLVNGGRENVSTTDYNHNDYLAQSAVPLRMETHGGEDVAIFSKGPMAHLLHGVKEQNYIPHVMAYAACIGKNKQHCISGSSGA

>Medaka_Alpi1_C4:12,378,719_ENSORLT00000008795_alkalinephosphatase,placental_ENSORLG00000006998]

MAKTHHFITGLLIFFSLQKALCISVDELEASYWNNKAKEALNAAIQVPRNLERAKNLILFLGDGMGMATVTAARMLKGQLARQSGEESSLVMDTFPYVALSKTYNVDEQMPDSAGTATAYLCGVKANYGTLGVTAATTRDNCLTSFGNNVSSILHRAKRAGKAVGIVTTTRVQHASPAASYAHSASRDWYSDSDLTEEALQNGCKDIAYQLINNTEINVILGGGRQYMFPKNTPDPEYPTLTGSRNDGLDLVEEWKKNKDTAKYVWDKKGFDAVNHRTTDNLMGLFEPKDCRYELERDPSMDPSLTEMTEKAIRILSKKPEGFFLFVEGGRIDHAHHGGQAKLALHEAVEFDRAIGRAAELTSELDTLTIVTADHSHVFAFGGYSARGNPVLGVSRELADDGKHFTTTVYGNGPGYQIANGSRPDVNDTVSSTNDYRQQAAVPLESETHGIEDVAIFAKGPMSHLIHGVQEQNYIAHVLAYAACLEPYENCRSHAAAFHLSLLLLLRSVCLPIMFLLFY

>Medaka_AlpiB_C13:18,337,006_ENSORLT00000011879_ENSORLG00000009477_NearCdAndEcel1,isItTgdDupl?]

WVMFFKLYHIPGATFASLLPAVEEENPEFWRAQAKQTLQSVLDRKLNTKVAKNILFFLGDGMGITTYTAARILKGQLQNQSGEETVMTMDTFPYVGLAKTYSVDFQIADSAATATAYLCGVKTNLNTVGVNAAARNGICRTQKGNEVTSILKWAKDAGKSVGIVTTTRVQHATPAGSYAHSASRTWYSDADMPDSAIGEGCTDISSQLLKNVDIDVIIGGGRQYMTPRGTKDPEYPADFSSGGKRRDGRHLIQEWQNMKNGKAAHYAWNKTEFDAIDPETTDYLMALFEPGDLRYEAERDPSMDPSIMETTEKAIRILQKNPKGFFLLVESGRIDQAHHQGNAYLALHEAVAFDGAIARGLELTSEDDTLTIATADHSHPLTFNGFPFRGQSILGKSPLWGTDMLPYTTLMYGNGPGHKIVNGKRPDIRNVNTNSMEYIQLSAAPLDSTTHSGEDVVVLARGPMAHLFSGIQEQNYIAHAMGYAACVGADLRHCQEHITPAVGRTSISPNGNGAAGGSATPISILLASLLALRILM

>Medaka_Alpl_C5:32,031,301_ENSORLT00000021361_alkalinephosphatase,liver/bone/kidney_ENSORLG00000017066]

TMLSALLIICSCLSVRSSGKPMFPENEKSPAFWNNGAQKTLKEALSTQELNKNKAKNLILFLGDGMGVPTVTAARILKGQLNGQSGEEYQLEMDKFPFVSLSKTYNTDAQVPDSAGTATAYLCGVKANEGTVGVSAAAVRSQCNTTKGNEVTSILKWAKDAGKSVGIVTTTRINHATPSAAYAHSVDRDWYSDNEMPSEALQAGCKDIARQLFENIPNIDVIMGGGRKYMFPKNMSDVDYPGVAKYNGVRNDGRNLVQEWIDRMKDQGGHYVWNKQQLLSLNPKKTSYLLGLFGPVDLPYDLERNADSDPSLTQMVEVAIKVLQNNPKGFYLLVEVGGRIDHGHHEGKAKQALYEAVEMDRAIGRADLLTSAQDTLTVVTADHSHVFTFGGYTRRGNTIFGLAPMVSDVDHKPFTSILYGNGPGYKLVGGARENVSTVDFAENNYKAQSAVPLTSETHGGEDVAVFAKGPLAHLLHGVHEQNFIPHVMAYAACIGQNRGHCPSGGTAALRPVFSSMAAVLTVTLLLC

>Medaka_ALPPL2_C4:12,379,014_ENSORLT00000008796_alkalinephosphatase,placental_C4:12,379,014_ENSORLG00000006998]

LRCTSFWFLLFLCVCEAALHEIEKEPAYWNAQAKAALNAALKLHPRHHRAKNLILFLGDGMGVTTVSAARILRGQMEGWSGEETVLAMDTFPYVALSKTYSVDKQVADSASTATAYHCGVKANAKTLGLNAKAVVYECNTTFGNEVHSVLRRAKAQGKSVGIVTTTRVQHASPAAAYAHSVSRKWYSDADLPSSAIQQGCVDIATQLVTNVDIDVILGGGRMYMTPQGTPDPEYPTSSSRKGDRKDKRNLINIWLKAKPKKKSHYVWNKKGFDEINVKTTDRLMGLFEPKDMKFEVYRNSSSDPSIVEMTEKAIQILRKNPKGYFLFVEGGRIDHGHHDNTAKLALRETVMFDAAIERAGQLTRESDTLTVVTADHSHVFTFGGSTPRGNPIFGVSRELADDGKHFTTTVYGNGPGYQIANGSRPDVNDTVSSTNDYRQQAAVPLESETHGIEDVAIFAKGPMSHLIHGVQEQNYIAHVLAYAACLEPYENC

>Tilapia_Alpi1_GL831204:1,034,254_ENSONIT00000015767_alkalinephosphatase,placental_ENSONIG00000012513]

MLNASFSWLITLSLSLSLLPLVPWDELDPLYWNNKGRNALHTALNKPRNSHQAKNVILFLGDGMGIPTVTAARILKGQLERKSGEESSLAMDSFPHLALSKTYNVDQQMPDSAGTATAYQCGVKANYGTLGVNAAAPRHNCQASHGNDVTSVLHRAKLAGKSVGIVTTTRVQHASPAAAYAHVASRDWYSDADLSEEAVQNGCRDIAYQLVYNTEIDVILGGGRTYMFPTTYQDPEYPTVKGARKDEKNLVDEWLKNKQNAKYVWNKADFDAINPATTDFLMGLFEPKDCRYELDRDPSMDPSLTEMTEKAIRILSKNPKGFFLFVEGGRIDHGHHATMAKKALHEAVEFDRAIERAADLVSDLDTLTIVTADHSHVFAFGGDSPRGNPVLGIADTQATDNKHFTTAVYGNGPGYKIANGSRPDVNESVSTDNNYLQQTAVPLDSETHGIEDVAIFAKGPMSHLFDGVQEQSYIAHVMAYALCIDPYVDCQLPEANHAVASHPSLLLLLMSLLLITFYPI

>Oni_Alpi2_GL831204:1021486:1032110_JHPbuilt]

DELDPLYWNNKGRNALHTALNKPRNSHQAKNVILFLGDGMGVSTVSAARILRGQMDGGSGEETILAMDTFPYVALSKTYSVDKQVADSASTATAYHCGVKANAKTVGLSAKAVPYECNTTFGNEVYSVLRRAKAQGKSVGIVTTTRVQHASPAAAYAHSVSRSWYSDADLPSSAREHGCVDIATQLVTNVDIDVILGGGRMYMTPKGTPDPEYPTSNSRKGDRKDRRNLIDVWLLLGLFEPKDMRFEVFRNSTRDPSIVEMTEKAIQILKKNPKGYFLFVERGRIDHGHHDGIAKLALTEAVMFDRAIHRAAQLTRESDTLTVVTADHSHVFTFGGNTPRGNPIFGLAPKKADDQMPFTSILYANGPGYVHINGTRENIEYMQQAAVPLDAETHGGEDVAIYAKGPMAHLFHGVKEQNYVAHVMAYAACLEPYTNC

>Tilapia_ALPL_GL831191:3,589,286_ENSONIG00000000682_Tilapia_OreoComisniloticus_ENSONIT00000000861_alkalinephosphatase,liver/bone/kidney]

MTALLIICFCLILGSLGKPQFPEQEKDPKFWNAWAQRTLKNALTLQELNKNKAKNLILFLGDGMGVPTVTAARILKGQLNGQNGEETQLEMDKFPFVSLSKTYNTNAQVPDSAGTATAYLCGVKANEGTVGVSAAAVRSQCNTTQGNEVTSILKWAKDAGKSVGIVTTTRVNHATPSAAYAHCVDRDWYSDNEMPAEALQAGCKDIARQLFENIPNIDVIMGGGRKYMFPKNASDIEYPNILKHSGTRKDGRNLVEEWVDRMRNKNGRYVWNKTQLLTLNPNNVDYLLGLFEPGDMTYDLERNTESDPSLTEMVDVAIKILKKNPSGFYLLVEGGRIDHGHHEGKAKQALHEAVEMDRAIGRAGLLTSDHETLTIVTADHSHVFSFGGYTPRGNTIFGLAPMLSDVDQKPFTSILYGNGPGYKVINGARENVSTVDYKGNNYQAQAAVPLNAETHGGEDVAVFAKGPLAHLLHGVHEQNYIPHVMAYAACIGQNREHCMSSSGSAGLRPAFSIVATILSAARLLC

>Fugu_Alkl_scaf_178:10,865_ENSTRUT00000010675_alkalinephosphatase_NP_001027823]_ENSTRUG00000004463]

RISFFVRLFVLFVCLQTTFLIEQEKDPKFWNTWAQQTLKNALSLQTLNQNKAKNLIFFLGDGMGVPTVTAARILKGQLSGQSGEETQLEMDKFPFVSLAKTYNTNAQVADSAGTATAFLCGVKANEGTVGVSAAAVRSQCNTTKGNEVTSILKWAKDAGKSVGLVTTTRVNHATPSASYAHSVDRDWLSDSVMPAEAVQDGCKDIARQLFENIPDINVIMGGGRKYMFPKNQVDVEYPDTAKYSGIRKDGRNLVGEWIEKMENKNGHYVWNKKQLLSLNPNNVDYLLGLFEPEDMTYDLERNPETDPSLTEMVEVAIKILKKNPNGFYLLVEGGRIDHGHHEGKAKQALYEAVEMDRAISRAGLMTSIHDTLTIVTADHSHVFNFGGYTHRGNTIFGLAPAVSDVDQKPFTAILYGNGPGYKLVNGGRENVSAIDYQENNYQAQAAVPLSMETHGGEDVAVFAKGPMAHLLHGVHEQNYIPHVMAYASCIGQNRQHCAGHNQGSRLRPALPSIAALFTLIRLLF

>Fugu_Alpp_scaf_43:1,103,898_ENSTRUT00000022198_alkalinephosphatase,placental_scaf_43:1,103,898_ENSTRUG00000008800]

TLCFGAVVGWSICLADDILKATLHELEKEPAYWDAQARATLGAALRLRPRDHQARNLILFLGDGMGVSTVSAARILRGQMEGGSGEETMLAMDTFPYVALSKTYSVDKQVADSASTATAYHCGVKANAKTLGLNANAVAYECNTTFGNEVYSVLRRAKAQGKSVGIVTTTRVQHASPAASYAHSVSRSWYSDSDLPESAIEQGCVDIAAQLVTNVDIDVILGGGRMYMTPRGTLDPEYPTSNSRKGDRNDKRNLIDVWLNAEPNKRSRYVWNKREFDEINIKTTDRLMGLFEPKDMKFEVFRNSTRDPSIVEMTEKAIQILSKNPNGYFLFVEGGRIDHGHHDSVAKLALTETVMFDRAIQRAAQLTRESDTLTVVTADHSHVFTFGGNTPRGNPIFGLAPKNADDGMPFTSILYANGPGYIHVNGSRENITMVDYNDDEYMQQAAVPLDAETHGGEDVAIYAKGPMAHLFHGVKEQNYIAHVMAYAACLEPYRTCPRTPTHSSSPAVKSPSNLLFFLLVLLLV

>Green_pufferfish_Alp_CUn_random:21,213,913_Q4S777_TETNGENSTNIT00000015366_ENSTNIG00000012197]

VMTATVALILGSCLILGSLGKPSFPEQEKDPKFWNTWAQQTLKNALGLQTLNQNKANNLIFFLGDGMGVPTVTAARILKGQLSGQSGEEGQLEMDKFPFVSLAKTYNTNAQVADSAGTATAFLCGVKANEGTVGVSAAAVRSQCNSTKGNEVTSILKWAKDAGKSVGIVTTTRVNHATPSASYAHSVDRDWFSDAEMPAEALQDGCKDIARQLFENIPNIDVILGGGRKYMFPKNQSDVEYPNIAKHSGTRKDGRNLVAEWTEKMKDSKGHYVWNKQQLVTLNPNMVDYLLGLFEPGDMTYDLERNSETDPSLTEMVDVAIKILKKNPNGFYLLVEGGRIDHGHHEGKAKQALYEAVEMDRAIQRAGLLTSVHDTLTIVTADHSHVFNFGGYTHRGNPIFGLAPAVSDVDQKPFTAILYGNGPGYKLVNGGRENVSTINYQENNYQAQAAVPLSSETHGGEDVAVFAKGPMAHLLHGVHEQNYIPHVMAYASCIGQNRQHCLQHNQASSLRPTLSSTAALLAAIKLLF

>Green_pufferfish_Alpi1_C1:18,545,349_ENSTNIT00000001814_ENSTNIG00000001649]

QQGYYGRLLLLLLKLLKTFSALHELEKEPAYWDAQASATLGAALKLRPRDHQARNIILFLGDGMGVSTVSAARILRGQMEGGSGEETMLAMDTFPYVALSKTYSVDKQVADSASTATAYHCGVKANAKTIGLNANAVAYECNTTFGNEVYSVLQRAKAQGKSVGIVTTTRVQHASPAASYAHSVSRSWYSDSDLPDSAIEQGCVDIATQLVTNVDIDVILGGGRMYMTPRGTPDPEYPTSNSRKGDRNDNRDLIDVWLKAKPNKRSRYVWNKMEFDEINIRTTDRLMGLFEPKDMKFEVFRNSTRDPSIVEMTEKAIQILNKNPKGYFLFVEVRGRIDHGHHDGIAKLALTETVMFDRAIQRAAQLTRESDTLTVVTADHSHVFTFGGNTPRGNPIFGLAPKNADDRMPFTSILYANGPGYVHINGSRENITMVDYYDDEYMQQAAVPLDAETHGGEDVAIYAKGPMAHLFHGVKEQNYVAHVMAYAACLEPYRNCPHPHTHSSSPGMNLPTILEFFLLGLLLLL

>Green_pufferfish_Alpi2_C1:18,552,681_ENSTNIT00000015356_alkalinephosphatase,placental_ENSTNIG00000012188]

MAGHQRMIVTGLILSILMQWTLSAPEKDKQELHADFWNNKGKQAIFTAMNVQPNIRRAKNMILFIGDGMGVPTVSAARIFKGQLGGRSGEESNLVMDTFPHLALSKTYNVDQQMPDSAGTATAYLCGVKANYGTLGVTAATPRYDCKAAIDNPVTSVLHRAKKAGKSVGIVTTTRVQHASPGASYAHTADRGWYADSDLSPEAIRDGCRDIAHQLISNTAIDVILGGGRQYMFSTTTQDPEYPSSKGTRNDGRDLVKEWMTNKTNAKYVWKKSDFDSVDPRKTDFLMGLFEPKDCRYELDRDPNMDPSLTEMMEKAIRILSKNPKGFFLFVEASGRIDHAHHAGNAKRALYEAVEFDRAIGRAAELTSELDTLSVVSADHSHVFTFGGYSPRGNPVLGVSRSLAGDNKRFTTIIYGNGPGYQINETRPDVNETISADKDYLQQAAVPLDSETHSIEDVAIFAKGPMSHLFHGVQEQSYIAHVLAYAACIEPYEDCGLPNHAGFLHPSLLLLLLAVCLLSLC

>Green_pufferfish_Alpl_C16:7,089,940_Q4RYZ7_TETNGENSTNIT00000017532_Alkalinephosphatase_ENSTNIG00000014299]

EEENPEFWRSQARKSLQSVLNRELNTNVSRNILFFLGDGMGMTTYTAARILKGQLQGRAGEETVLTMDTFPSVGLAKTYSVDFQIPDSAATATAFLCGVKTNLNTVGVSAAARNGVCKMQKGNEVTSILKWAKDAGKSVGIVTTTRVQHATPAASYAHSASRKWYSDADVPESAKKEGCTDISSQLLNNTDIDVIMGGGRKYMTPRGTKDPEYPLDFFSRGQRKDGRDLTKEWQSMKAGKVARYVWNKAGFDAVDPETTDYLMALFEPGDLRFDVDRDPKLDPSIVEMTEKAIRILRKNPKGFFLLVEGGRIDQAHHDGRAYMALHEAVAFDRAVARGLELTHEQDTLTVVTADHSHPLTFNGFPFRGQSILGKSPLWGTDFRPYTTLMYGNGPGYKLINGSRPDLRDVNTKHKDYVQLSAAPTDSTTHSGEDVVVLARGPMAHLFQGVHEQNYLAHAMAYAACVGADLRHC

>Fugu_Alpi3_scaf_123:791164:797773_JHPbuilt]

VEEENPEFWRSQAQKSLQSVLDRKLNTNVSRNILFFLGDGMGVTTYTAARILRGQLQNQSGEETVMTMDTFPSVGLAKTYSVDFQIPDSAATATAFLCGVKTNLNTIGVSAAARNGICKTQKGNEVTSILKWAKDAGKSVGIVTTTRVQHATPAASYAHSASRKWYSDADVPESAKKDGCTDISSQLLNNTDIDVIMGGGRKYMTPRGTKDPEYPWDFLSRGRRKDGRDLTKEWQSMKAGLFEPGDLRFEVRDPKVDPSIVEMTEKAVRILRKNPKGFFLLVEGGRIDQAHHDGRAYMALHETIAFDDAIAKGLELTDEHETLTVVMADHSHPITFNGFPFRGQSILGKSPLWGTDFKPYTTLMYGNGPGYKLANGSRPDLRDSASFSPETKDYVQMSAAPTESTTHSGEDVAVLARGPMSHLFQGVHEQNYIAHAMAYAACV

>Fugu_ALPP_scaf_43:1,103,412_ENSTRUG00000008800_ENSTRUT00000022197_2dHalf,AA480-954

FWYNKGRQALFTAMNVQPNIKKAKNMILFIGDGMGVPTVTAARMLKGQLGGRTGEEADLVMDTFPHLALSKTYNVDKQMPDSAGTATAYLCGVKANYGTLGVNAATPRYDCNATFGNEVTSVLHRAKKAGKSVGIVTTTRVQHASPGASYAHIANRGWYADSDLSPEAVAGGCRDIAHQLVYNTEIDVILGGGRQYMYPKTMQDPEYPASRGSREDGRNLILEWVKDKTNVKYVWEKSEFDSVNPSHTDFLMGLFEPKDCRYELDRDPKMDPSLTEMTEKAIQILSKNPKGFFLFVEDKPSSGRIDHAHHGGKAKRALYETVEFDQAIGRAAELTSELDTLTVVSADHSHVFAFGGDSPRGNPVLGVSSLLAEDKKHFTTAVYGNGPGYQIANGTRPDVNETISSDKDYRQQAAVPLDSETHGMDDVAIYAKGPMSHLFHGVQEQSYIAHVLAYAACIEPYDDCNLCSCCFHKVH

>Cionaintestinalis_Alp_NP_001027596.1|endoderm-alkalinephosphatase[Cionaintestinalis]

MLDMMFHQGLLLLCASVAVIGQDLTEKSAAYWEQVSEAELLETLQYQKLNIKKAKNVIIFIGDGMGVTTVTAGRILKGQNSGASGEETKLAMDKLPYTGVSRTYSVNRQVSDSASTATAFLTGVKTNDYVLGLNGNSVKGICAGSINESNLLTSVLLEAKMAGMSAGIVTTTTINHATPAAAYANSPDRLWYSDAEMTAEAKENGCKDIAQQFIDKSDQFTVVLGGGRQYFKPNTTFDVEYTDRANLRLDGQDLIEVWKAKQSDRNSAYVWNKEQFDQVDVAKTDSLLGLFEPSHMNYEAHRAQDGAGEPSLKDMTSKAIRMLKKNDQGFILLVEGGKIDHGHHAGKAYLALHDLVALDDAIEAAVEMTSDDETMIIVTADHSHVFTIGGYSHRGNPIFGAAPNVNNPKLVDDGKPFTTLLYGNGPGHSTLNGVGSCERENITLIATDDPNYKQQSAVPLPSETHGGEDVVIMARGPMAHLFEGVHEQSYIAHIIRYATCIGKKSKNCAAQLEQSTDLIFVSFLGFRLSSGQAQLALYITFGLLMAACIIAIAANLQLCRMARQSARKHEDPKVLNEKV

>Cionasavignyi_BAA92180.1|endoderm-alkalinephosphatase[Cionasavignyi]

MSMICFVTMMCVCSLGTAQVLTERSAHFWELRNQAELDEAITKQSLNIRKAKNVIIFVGDGMGITTITSGRILKGQVSGTSGEETKLAMDKFPFSGISKTYSVNKQVSDSASTATAFLTGVKTNDFILGLTGSAQRGICKGSIDENNIVTSVLIEAKNAGKSAGFVTTTRINHATPGATYAHTPERMWYGDADLTEEAKANGCKDVAQQFIDNSHLFTVALGGGRQYFRPNTTQDEEYPNKTNARLDGQDLIEQWKQIQLQQGNRAAYVWNATEFAGINPDNTDSLLGLFQPKDMHYEAHRSGDVAGEPSLSEMTAKAISLLKKNEEGYILLVEGGRIDHGHHEGNAYLALHDLVAFDDAIDTAVQMTSDDETMLIVTADHSHVFTIGGYSDRGNPIFGLAPNAIKPTLGDDNKTFTTLLYGNGPGYAFESCERENVTGVPTDVSTYLQQSAVPLSYETHGGEDVIIMSRGPMAHLFEGVHEQTYIAHVIRYATCIGKLSKDCNERFNPPKENGVILYFLGISMTSSKAVLALYVTLALLIVTSIVAIAANIHIYRMVSSKPKSEQMQKV
