# Supplementary material for: Dynamic Evolution of the LPS-Detoxifying Enzyme Intestinal Alkaline Phosphatase in Zebrafish and Other Vertebrates
Source: Front Immunol. 2012 Oct 12;3:314. doi: 10.3389/fimmu.2012.00314 (PMC3469785; doi:10.3389/fimmu.2012.00314)
Supplement: Supplementary Data sheet S2 — Alp sequences used to generate Figure 3. [file 35467_Guillemin_DataSheet2.DOCX]

>Human_ALPI_Hsa2:233,320_NP_001622_[Homosapiens]_ENSG00000163295]

MQGPWVLLLLGLRLQLSLGVIPAEEENPAFWNRQAAEALDAAKKLQPIQKVAKNLILFLGDGLGVPTVTATRILKGQKNGKLGPETPLAMDRFPYLALSKTYNVDRQVPDSAATATAYLCGVKANFQTIGLSAAARFNQCNTTRGNEVISVMNRAKQAGKSVGVVTTTRVQHASPAGTYAHTVNRNWYSDADMPASARQEGCQDIATQLISNMDIDVILGGGRKYMFPMGTPDPEYPADASQNGIRLDGKNLVQEWLAKHQGAWYVWNRTELMQASLDQSVTHLMGLFEPGDTKYEIHRDPTLDPSLMEMTEAALRLLSRNPRGFYLFVEGGRIDHGHHEGVAYQALTEAVMFDDAIERAGQLTSEEDTLTLVTADHSHVFSFGGYTLRGSSIFGLAPSKAQDSKAYTSILYGNGPGYVFNSGVRPDVNESESGSPDYQQQAAVPLSSETHGGEDVAVFARGPQAHLVHGVQEQSFVAHVMAFAACLEPYTACDLAPPACTTDAAHPVAASLPLLAGTLLLLGASAAP

>Human_ALPL_Hsa1:21,835,858_NP_000469_ENSG00000162551]

MISPFLVLAIGTCLTNSLVPEKEKDPKYWRDQAQETLKYALELQKLNTNVAKNVIMFLGDGMGVSTVTAARILKGQLHHNPGEETRLEMDKFPFVALSKTYNTNAQVPDSAGTATAYLCGVKANEGTVGVSAATERSRCNTTQGNEVTSILRWAKDAGKSVGIVTTTRVNHATPSAAYAHSADRDWYSDNEMPPEALSQGCKDIAYQLMHNIRDIDVIMGGGRKYMYPKNKTDVEYESDEKARGTRLDGLDLVDTWKSFKPRYKHSHFIWNRTELLTLDPHNVDYLLGLFEPGDMQYELNRNNVTDPSLSEMVVVAIQILRKNPKGFFLLVEGGRIDHGHHEGKAKQALHEAVEMDRAIGQAGSLTSSEDTLTVVTADHSHVFTFGGYTPRGNSIFGLAPMLSDTDKKPFTAILYGNGPGYKVVGGERENVSMVDYAHNNYQAQSAVPLRHETHGGEDVAVFSKGPMAHLLHGVHEQNYVPHVMAYAACIGANLGHCAPASSAGSLAAGPLLLALALYPLSVLF

>Human_ALPP_Hsa2:233,243,244_NP_001623_[Homosapiens]_ENST00000392027]

MLGPCMLLLLLLLGLRLQLSLGIIPVEEENPDFWNREAAEALGAAKKLQPAQTAAKNLIIFLGDGMGVSTVTAARILKGQKKDKLGPEIPLAMDRFPYVALSKTYNVDKHVPDSGATATAYLCGVKGNFQTIGLSAAARFNQCNTTRGNEVISVMNRAKKAGKSVGVVTTTRVQHASPAGTYAHTVNRNWYSDADVPASARQEGCQDIATQLISNMDIDVILGGGRKYMFRMGTPDPEYPDDYSQGGTRLDGKNLVQEWLAKRQGARYVWNRTELMQASLDPSVTHLMGLFEPGDMKYEIHRDSTLDPSLMEMTEAALRLLSRNPRGFFLFVEGGRIDHGHHESRAYRALTETIMFDDAIERAGQLTSEEDTLSLVTADHSHVFSFGGYPLRGSSIFGLAPGKARDRKAYTVLLYGNGPGYVLKDGARPDVTESESGSPEYRQQSAVPLDEETHAGEDVAVFARGPQAHLVHGVQEQTFIAHVMAFAACLEPYTACDLAPPAGTTDAAHPGRSVVPALLPLLAGTLLLLETATAP

>Human_ALPPL2_Hsa2:233,271,553_NP_112603_ENST00000295453_[Homosapiens]

MQGPWVLLLLGLRLQLSLGIIPVEEENPDFWNRQAAEALGAAKKLQPAQTAAKNLIIFLGDGMGVSTVTAARILKGQKKDKLGPETFLAMDRFPYVALSKTYSVDKHVPDSGATATAYLCGVKGNFQTIGLSAAARFNQCNTTRGNEVISVMNRAKKAGKSVGVVTTTRVQHASPAGAYAHTVNRNWYSDADVPASARQEGCQDIATQLISNMDIDVILGGGRKYMFPMGTPDPEYPDDYSQGGTRLDGKNLVQEWLAKHQGARYVWNRTELLQASLDPSVTHLMGLFEPGDMKYEIHRDSTLDPSLMEMTEAALLLLSRNPRGFFLFVEGGRIDHGHHESRAYRALTETIMFDDAIERAGQLTSEEDTLSLVTADHSHVFSFGGYPLRGSSIFGLAPGKARDRKAYTVLLYGNGPGYVLKDGARPDVTESESGSPEYRQQSAVPLDGETHAGEDVAVFARGPQAHLVHGVQEQTFIAHVMAFAACLEPYTACDLAPRAGTTDAAHPGPSVVPALLPLLAGTLLLLGTATAP

>Mouse_Alppl2_Mmu1:88,983,265_ENSMUST00000027455[Mouse]

MWGACLLLLGLSLQVCPSVIPVEEENPAFWNRKAAEALDAAKKLKPIQTSAKNLVILMGDGMGVSTVTATRILKGQQQGHLGPETQLAMDRFPHMALSKTYNTDKQIPDSAGTGTAFLCGVKTNMKVIGLSAAARFNQCNTTWGNEVVSVMHRAKKAGKSVGVVTTTSVQHASPAGTYAHTVNRGWYSDAQMPASALQDGCKDISTQLISNMDIDVILGGGRKFMFPKGTPDQEYPTDTKQAGTRLDGRNLVQEWLAKHQGARYVWNRSELIQASLNRSVTHLMGLFEPNDMKYEIHRDPAQDPSLAEMTEVAVRMLSRNPKGFYLFVEGGRIDHGHHETVAYRALTEAVMFDSAVDKADKLTSEQDTMILVTADHSHVFSFGGYTQRGASIFGLAPFKAEDGKSFTSILYGNGPGYKLHNGARADVTEEESSNPTYQQQAAVPLSSETHSGEDVAIFARGPQAHLVHGVQEQNYIAHVMAFAACLEPYTDCGLASPAGQSSAVSPGYMSTLLCLLAGKMLMLMAAAEP

>Mouse_Alpi_Mmu1:88,994,579_ENSMUST00000113270[mouse]

MQGDWVLLLFLGLRIHLSFGIIPAEEENPAFWNKKAAEALDAAKKLQPIQTSAKNLIIFLGDGMGVPTVTATRILKGQLEGHLGPETPLAMDLFPYMALSKTYNVDRQVPDSAGTATAYLCGVKANYKTIGLSAAARLDQCNTTFGNEVFSVMYRAKKAGKSVGVVTTTRVQHASPAGTYAHTVNRNWYSDAEMPASALQDGCKDIATQLISNMDIDVILGGGRKFMFPKGTPDPEYPSDSNQSGTRLDDQNLVQTWLSKHQGARYVWNRSELIQASQDPAVTHLMGLFEPTEMKYDANRNPSVDPSLAEMTEVAVRMLSRNPQGFYLFVEGGRIDQGHHAGTAYLALTEAVMFDSAIEKASQLTNEKDTLILITADHSHVFAFGGYTLRGTSIFGLAPLKALDDKSYTSILYGNGPGYELKSGNRPNVTEAQSVDPNYKQQAAVPLSSETHGGEDVAIFARGPQAHLVHGVQEQNYIAHVMAFAGCLEPYTDCGLAPPAGQSPVITPGQATTTNNAAGQATTTNNAAGQATVLLSLQLLVSMLLLVGTAMVVS

>Mouse_Akp3_Mmu1:89,021,583_ENSMUST00000044878[mouse]

MQGTWVLLLLGLRLQLSLSVIPVEEENPAFWNKKAAEALDAAKKLQPIQTSAKNLIIFLGDGMGVPTVTATRILKGQLEGHLGPETPLAMDRFPYMALSKTYSVDRQVPDSASTATAYLCGVKTNYKTIGVSAAARFDQCNTTFGNEVFSVMYRAKKAGKSVGVVTTTRVQHASPSGTYVHTVNRNWYGDADMPASALREGCKDIATQLISNMDINVILGGGRKYMFPAGTPDPEYPNDANETGTRLDGRNLVQEWLSKHQGSQYVWNREQLIQKAQDPSVTYLMGLFEPVDTKFDIQRDPLMDPSLKDMTEAAVKVLSRNPKGFYLFVEGGRIDRGHHLGTAYLALTEAVMFDLAIERASQLTSERDTLTIVTADHSHVFSFGGYTLRGTSIFGLAPLNALDGKPYTSILYGNGPGYVGTGERPNVTAAESSGSSYRQQAAVPVKSETHGGEDVAIFARGPQAHLLHGVQEQNYIAHVMAFAGCLEPYTDCGLAPPADESQTTTTTRQTTITTTTTTTTTTTTPVHNSARSLGPATAPLALALLAGMLMLLLGAPAES

>Mouse_Alpl_Mmu4:137,297,648_ENSMUST00000030551]

MISPFLVLAIGTCLTNSFVPEKERDPSYWRQQAQETLKNALKLQKLNTNVAKNVIMFLGDGMGVSTVTAARILKGQLHHNTGEETRLEMDKFPFVALSKTYNTNAQVPDSAGTATAYLCGVKANEGTVGVSAATERTRCNTTQGNEVTSILRWAKDAGKSVGIVTTTRVNHATPSAAYAHSADRDWYSDNEMPPEALSQGCKDIAYQLMHNIKDIDVIMGGGRKYMYPKNRTDVEYELDEKARGTRLDGLDLISIWKSFKPRHKHSHYVWNRTELLALDPSRVDYLLGLFEPGDMQYELNRNNLTDPSLSEMVEVALRILTKNLKGFFLLVEGGRIDHGHHEGKAKQALHEAVEMDQAIGKAGAMTSQKDTLTVVTADHSHVFTFGGYTPRGNSIFGLAPMVSDTDKKPFTAILYGNGPGYKVVDGERENVSMVDYAHNNYQAQSAVPLRHETHGGEDVAVFAKGPMAHLLHGVHEQNYIPHVMAYASCIGANLDHCAWAGSGSAPSPGALLLPLAVLSLRTLF

>Amphioxus_Alp2_XP_002598196_BRAFLDRAFT_119115[Branchiostomafloridae]

MARASIDEALRLQTLNTNVAKNVVLFLGDGMGVSTVTTARILKGQKAGNPGEETVLAMDSLPYTAMSKTYNIDKQVPDSAACATAFLCGVKTNLGLLGLDGRATYKNCSSAKGHEVESIIEHAEKAGKSTGIVTTTRVTHATPAAGYAHTASRNWEADSDLTAEARENNCTDIAFQLVEEAPDIEVILGGGRRAFLTNNMTDPEDGSKGTRKDGRDLIQRWLDRKTGPARYVWNETEFNSVDPESVDYLLGLFEPSSMQYSVDRLNDTAGEPSLAAMTRKAIQVLQKNRNGFFLLVEGGRIDQAHHASKAVKALEETVVFDDAVQVANDMLDTSDSLIVVTADHSHTLTFAGYPDRGHPIFGQNVYTSSTPDYDWDKLPYTTLLYGNGPGYTLVETTNGNDTQVTRQNITDVNTADKEYKQQSAAPLRSETHGGEDVIIMADGPMAHLFHGVQEQHYIAHVMMYAACLGEYTEHCDKPGTPKPVRDAGPAARGSYYTVALTTVLSGLLMTTATHF

>Fly_Aph-4-RA_Dmel3R:26,874,654_FBtr0085733_brain&MalpighianTubules]

MHCLVILGFLLGSLVAFSWAGVTTQPPPLIRTLSAGGDIGPQFDVGKTKEPEDAEFWHNVGLRQLEKTIKQAQRVKEDSYQKKARNIIIFIGDGMGISTISAGRIYKGQYLKHGYGEEETLVFDDFPNTGMAKTYNVDKQVPDSAGTATAIFSGSKTHYGAIGMDATRSKKNGQQGRVQSVMEWAQKEGKRTGVVTTTRITHATPAATYAHIYDRDWECDTEVPAESVGFHVDIARQLVENAPGNRFNVILGGGMSPMGILNASEVKTTIFEGPTETICTRGDNRNLPAEWLAHHANDTVPPALVHNRKDLLNVNVKKVDHLMGLFRNNHITYSIAREAGEPSLQEMTETALGILERGDESNGFVLLVEGGRIDQGHHMNYARAALHELYEFDLAIQAAVNNTDPDETLILVTADHSHAVTFNGYALRGADILGTANSHEKNDPMFYETISYANGPGYWDHLANDSRPQNSSNMWMPLKHFTAEERAAPTYRHLATVPRKDETHGGEDVAVFAYGPGSSLIRGVFEQNYLAYVMSYAGCLGPAKDFDDSCEDHKDGQKDRPLDKPNPKRNGATVVGASLIPILTAATAAILRGRGL

>Fly_CG8105-RA_DmelX:15,481,766_FBtr0074048]

MLLQLRRLAILFLLHALIPILALSVPMVEQVQPADELLSSHWLRQAQSQLRERLARTKDSIADVRQAKNVVMLLGDGLSITTLTAARILKGQRRGGRGEDAQLAVEQFPFSGLSKTYCIDEQTPDSACTATAYFGGVKTHSGTVGQSGSGERVDSVLQWAQRAGKATGVVTTTRLTDASPAGAYAHVSRRGEELEIARQLVEEEPGRNLNVILGGGLGKFADERRDGKDLLAQWRESNPNGCFSRSLKELRNCTDATGTLLGIFSDSHMSYHLAASKELPRLCDMAAAAIEKLERKSQEDGNGYFVFIEGGRIDHGHHETRVGYALDEMVEFDAAVETVIQMTDPRDTLVVVTADHSHTLSMSGYAKRGTPILGMDAQQRDVNGVPYSTLNYAIGKWQSLDKDGRRESPSKTLSPTSYTPSYIHGRKGVHSGEDVVVFAMGPQSHLFGGVMEQNLLPHIMGYAACLGNGLTLCNQDKDADMIA

>Fly_CG1809-RA_Dmel2R:5,449,154_FBtr0088520]

MARRFILLLVLAVCLSGSASGQRDHPRGRSFDAESHPIKYAEEQQTQYWVDKAQAKLLSKLAEQESATTNKAKNVILFLGDGMSVHTVTATRNLLGDSAEQVYFEGFPYTGLSKTYCVNRQVADSACTATAYLGGVKANYGTIGVNANVSRYSCDGAANEEDRVLSIAHWAQAAGKDAGLVTTARVTHASPAGVYAHIADRNWENDWEVANRECDPEQTIDIARQLVEQPVGQQLKVILGGGRKNFIDATVNDEEGYPGKRTDGRHLIRSWLDQKKEANVSAKYVWSRKGLSLVDLENTDYLLGLFANDHLPYNGDRDRKRSQLADPSLSELTEAAITVLSRNDKGFFLFVEGARIDMAHHDTFAKRSLEDTAEFARAVQKARELTSEDDTLIVVTADHAHVMSINGYPYRDQEITGLAQLADDNLPYTILSYANGPGYYSGYNRAEGRALLKEKLVADSDYQYPTLAPLDAETHGGDDVAVYASGPYAQYFSGNYEQSNIPALMARAAGIGPYA

>Fly_CG3292-RA_Dmel2R:18,091,199_FBtr0071783]

MKTFVAISLVLCTLVATSLAASIDMPEIHNQFQLVGGSVSTAKTREGNLIDPNAMAKGKPGPEEEKNAQFWYDLAYEEIAKRLEQPQLDKRKAKNVILFLGDGMSLSTVAAARIHKGQLKGNTGEEDSLSFEKFPYTGLSRTYCSNAQVPDSACTATAYLCGVKTNIVALGITAAVSFNNCSGSEDPANQVDSIAAWAQAAGKATGIVTTTTLTHASPSGAYAKTTNRFFESDTDILTYGEGQNDPATCTDIATQLITQAPGKNFDVMLGGGIGKFLPNTITDPFNKKGERSDGVNLLSRWQGLHPGGVLAYNRNQLLSVNVSKITNLIGTFRSGVMSFNKMADPKEEPTLAEMTRKAIEVVSKRDGGYFLFVEGGLIDYGNHFNSPTYSLSETLQFEQAVQEALDITDPEETLIVVTSDHAHPLTISGYPGRGTPILGLNQDDTDVNGVKYATLNYAVGTNQYLDEHGQRIDLTDQIGAEDFIHPSYIHGTIGVHAGDDVGIFATGPQSHLFTGVMQQSTIPHLMAYASCIGNGPTLCDNED

>Fly_CG3290-RA_Dmel2R:18,093,510_FBtr0071782]

MHISQRRCLYPIFLFLLAFVGTALGRVQEVHNVLELTGQKASKLRFSTDVATGKYTPPEEMDPQFWYKLADEEIAKRLQLPQPNSDRAKNVIMFLGDGMPISTVTAARILKGQRQGNTGEESSLSFEKFPYSGLSRTYCANAQVPDSACTATAYLCGVKTNIINIGVSAAVNFNNCTASQDPANRLTSIAEWAQNAGKSTGFVTTTTLTHASPSGAYAKTANRMWQSDTDVTSYGVDASTCVDMATQLVTQTPGKNFEVMFGGGMGKFLPKTTKDSHGKYGERSDGVNLLSRWQGLHDKGVLVTNRKQLLNLDVSAASSIIGTFQSGLMDFHMDADPTYQPTLSELTEVAIKKLSHNENGYFVFIEGGLIDYGNHYTQAGYALDEALEFEKAIQLAREMTNISDTLIVVTADHGHAVSIAGYPGRGTPILGLNQHDTDINGVKYSVLNYAAGPNQYLDEHGQRIPLDDILGPDDAISPSYIDKEIGVHSGEDVGIWASGPQSHLFTGVMQQSTIPHLMAYASCVGSGKQVCGSSGRKT

>Fly_CG3264-RA_Dmel2R:18,096,412_FBtr0071781]

MGSSQRQLVLPILLLLALAGTALCGVQEVHNVLELTGQAANKLKFSTDVATEKYTPPEEMDPQYWYNIADEEITKRLQLPQPNSQKAKNIIMFLGDGMPLSTVAAARILKGQRQGNTGEESSLSFERFPYTGLSRTYCSNAQVPDSACTATAYLCGVKTNIVNIGVSAAVEYNNCTASQDPANRLTSIAEWAQNAGKSTGIVTTTTLTHASPSGAYAKTANRMWECDTDVTSYGVDANSCIDMATQLVTQTPGKNFEVMFGGGMGKFLPNSIVDSHGNPGERSDGVNLLSRWQGLHDGGVLVTNRNQLLKLNVSAVSSVIGLFQSKLMNFHLEADDTYQPTLSELTEVTIKKLSQNKEHGYFAFIEGGLIDYGNHYTKAGYALDEALEFEKAIQLARDMTDIEDTLIVVTADHGHAVSIAGYPGRGTPILGINQHDTDINGVQYSVLNYAAGPNQYLDENGQRIPLDDILGSDDAITPSYIPKDQGVHSGEDVGIFASGPQSHLFTGVMQQSTIPHLMAYAACIGSGKQVCDN

>Fly_CG5150-RA_Dmel3L:5,588,646_FBtr0077086]

MLSLKFWIRLGLLTVVWGAAVPGRAPVDEERMHPHLPTSPRLRTISGEETQEFWHSASKKLIREKLEFVRNTKKAKNIILFLGDGMGLATLAAARSYIGGEELKLSFEEFPFTGLSKTYSVDKIVPDSACTSTSYLCGVKANYGTIGVNAHVKRGDCAAMANETNHVFSLGKWAMDAGKAAGLVTTTRVTHASPSGVYAHVADREWENNAVLEEACGELSDGLQDIAVQLIHGEVGSKLKVMLGGGKRSFYSPEHYDKGRRTDGRNLVEEFEALDAGNTFVKTQKKLLNVNATETGRLLGLFSKSHMHYHLEQLADPDNNEPTLEEMTQKAIEVLETEEQGYFLFVEGGKIDISHHETMARIALDETAELSKAVKKAREMTNPEETLIVVTSDHSHTFSVSGYQPRGSDIFGAAKANGKDGKPYLALSYANGKSFQDYYNTETHERVDPTSLPTIGDFDQLFPAMVPLESETHGGEDVGVFASGPWAHLFTGVYEQNTIPHMMAFAACVGDGLTACD

>Fly_CG10827-RA_Dmel3R:16,830,882_FBtr0084048]

MFRLLNALLIAIVLVAHPAWSAPECGRTPEECREHRMHPDMPEPERHGKAFRVVDGEDTNAYWRQQGVQFVQQKLASEPNKRQAKNVILFLGDGMGVTTTSAARNLLGGEEKSLSFENFPFTGLSKTYSVDKIVPDSACTATAYLCGVKGQEGTIGVNGQVPRTDCKVMLDESTHVDSIAKWAMEAGKWAGLVTTTRVTHASPSGVYAHIAERDWENDAEVATDCGAGSGINDIAYQLARGEVGSKLKVIMGGGRKHFVDSSLESWGKRTDGLNLIDEFKSASENNVYVTTAAELAAVDVTKTDRLLGLFNDDHLQYLMETTTESSQPTLEQMTRKAIEYMSQNEQGYFLFIEGGRIDQAHHINQARMALNETIEFSKAIAAAQELTSEDDTLLVVTADHSHVFTYAGYSYRGSDVFGKTPVNGHDGKPYMVLSYANGPSYENFYDAETMERKDPTTVVKGDHDDEFPSGVPIDMDSHGGDDVPVYALGPWAHLFTGVYEQSTIPHMMAYASCLGDGHTMCK

>Fly_CG8147-RA_Dmel3R:5,320,525_FBtr0082060]

MRLQLFFFLGLSVLVSGGESSHDAERRMHPVFSLMGTGSNPIGGGRYKRAMPQIVFDAVRSEERYAEYWQGLAAQTLDQQLESKLRLNTQLARNVMLFIGDGMSIPTITAGRVYLGGEEKQFAFEQFPYVGLSKTYCANMQVADSACTATAYLGGVKANYGTIGVSAAVQFKDCQAQAQAAHHVSSIAAWAQKQGMATGLVTTTSVTHASPAGVYAHLANRNWENDAEVVGDNGDPDLCPDAAAQLINSPVGQKLNVIMGGGRENFLPKGVTDSSGAPGRRLDGRNLIDEWKNQHTNSAQYVENRRELLNLSNHTSRVLGLFAPYHMAYHLDASPAEQPTLEEMVQSAMEILERQSAGRGYFLFVEGGRIDHGHHDTLALRAIDETAEFDKAVRFARSHTSTDDTLIVVSSDHSHTMSLAGYSSRKNDIFGINDGQLAADDLPYATLSYANGPGYDSNYLREGGAVRRKNLRAINMKNKDFMFPSTVPLESETHGGDDVAVFASGPYAQLFTGVFEQHFIPHALGYASCLSDRNMCVDGGVARRPR

>Ciona intestinalis_Alp1_CinHT000075.1:5,734_Alp_Q9NL48_CIOIN_ENSCINT00000000365_NP_001027596]

MLDIMFHQGLLLLCVSVAVIGQDLTEKSAAYWEQVSEAELLETLQYQKLNIKKAKNVIIFIGDGMGVTTVTAGRILKGQNSGASGEETKLAMDKLPYTGVSRTYSVNRQVSDSASTATAFLTGVKTNDYVLGLNGNSVKGICAGSINESNLLTSVLLEAKMAGMSAGIVTTTTINHATPAAAYANSPDRLWYSDAEMTAEAKENGCKDIAQQFIDKSDQFTVVLGGGRQYFKPNTTFDVEYTDRANLRLDGQDLIEVWKAKQSDRNSAYVWNKEQFDQVDVAKTDSLLGLFEPSHMNYEAHRAQDGAGEPSLKDMTTKAIRMLKKNDQGFILLVEGGKIDHGHHAGKAYLALHDLVALDDAIEAAVEMTSDDETMIIVTADHSHVFTIGGYSHRGNPIFGAAPNVNNPKLVDDGKPFTTLLYGNGPGHSTLNGAGSCERENITLITTDDPGYKQQSAVPLPSETHGGEDVVIMARGPMAHLFEGVHEQSYIAHVIRYATCIGKKSKNCAAQLEQSTDLIFVSFLGFRLSSGQAQLALYITFGLLMAACIIAIAANLQLCRMARQSARKHEDPKVLNEKV

>Ciona intestinalis_Alp3_Cin9:6,188,305_F6U0P5_CIOINENSCINT00000013633]

FQMIHQILRTCVCLIVLSAQIFTAREETAEYWNEAAEKTLHKIYGKENIKRNPAKNVILLIGDGMGLSTATAGRILKGQNEGRPGEETVSALDKFPNVGLSKVYCVDKQSPDSASTATAILSGVKTKFGVLGLNGRAVHKKCSSAAGNEVESILTRSANLGKSTGIVTTTEVQHATPAAAYAHSASRGWYVDSAMNSTTKSEGCKDISLQLLAMGRKINVVLGGGRAYMRPDGAYDEEYFSRRPGMRDDGRDLIREWKNLQPSSKARYVWRRSALNAVNADNTDYLLGLFQPKELRYDHDRNNDIAGEPSLEEMTEKAIQILRKNPSGYFLLVEGGKMDHAHHYNQANIALREFLVFEKAVQKAMDMTSTEDALIVVTADHGHVFTLGGYADRGNPIHGYARNHNEPRKAVDKHQYTSIAYANGPGYTLDTATGKRKGITGDTTRDIAYRYQAAVPLTSETHSAEDTVVFARGPMSYLFNGVYEQNYIAHALMFASCVG

>Ciona intestinalis_Alp4_Cin1:7,085,341_F6W9Y0_CIOINENSCINT00000014537]

AENTKEFWYRNGLQGIESSVDTFAQLNPTRAKNVVMMIGDGMNIATLTAGRIAIGQKSGASGEEHVTAIDQLPHTGIAKTYSVDYQTPDSAATATAFLTGVKTKQGTLGVRAGATNCDTDAVTSALEHAISQGKSTGIVTTTRVQQATPGAGYAHSLQRSAYTDRLLTMNGRGCKDISMQLYEKRRQIQVILGGGRSHMRPPSVFDEEGLGNRGDRGDNRDLIQMWRNDMERMNGSYVWNKRAFDQVNPLTTDYLLGLFSPLDMEFIINKPRNQRYDEPTLEEMTRKAIQILSKNPNGFFLLVEGGMIDKGHHHGRAKFALEEFHEFDKAVAAVKSMTSEQDTLLIVTADHGHMFTFGGGAVRGNSVYGLAKNNSRPDIALDGRPFTSILYGNGPGYSMYKGQRRYPGPWVANSKFYKAQSAVPLRAETHSAEDVAVYASGPWSHFISGVHEQSYLAHVMFYSACLGPLYTTYPHCATSLNTIQPIPNPGRIKHDEPPTPNASTQDKKR

>Ciona intestinalis_Alp5_Cin1:7,079,871_F6W9J4_CIOINENSCINT00000014541]

MKYRDRANNCIDISLQLLKKRRDIQVLLGGGRSYMRSMDARDEEGYKGDRKDGRDLIQEWKEDMERRGGRYVWNISSLNDINPSSTNYLLGMFNARHMIYSVRRKNEPTLAQMTAKAIQILSKNQKGFILLVEAGLIDVAHHQGRARFALEEFHELERAVEVAKTATTEEDTLLIVTADHGQMFMFGSGSVRGNPVYGLAPDNDNPGEAADGKRYTSLLYGTGPGNAAVQKSLSRQEVSTRVSRWKNYVVQSAVPMRGATHSAEDVVVFGRGPMAHMISGVHEQSYIGHVMMYAACLGPQYQHFSHCKDNGPTKR

>Ciona intestinalis_Alp7_Cin11:2,941,162_ENSCINT00000032115]

MHPPPVSRDSKKEKSAQRQALAGENTAQFWYDSAAKELDEALLLEKRNKNVAKNVILFLGDGMGIPSITSGRILKGQNNGQSGEETKLMMDTFPHAGLSKTYNVDHQVPDSAGTGTAYMTGVKTDMAVLGYNAAITPGVCSTTPGNEVSSVLEDSHKEGKAVGVVTTTRLNHATPGSAYAHSAYRYWYSDADLPDEAKINGCKDIAYQLYAALPNIQVALGGGRGYFRPNTTLDEEYKTNNFRTDGQELIEKWKNNMEDMGRNAKYVWNKQQFDAVDASSVDHLWGMFEPKDMNYETDRSTDGAGEPSLAEMTQKAIEILQKDEDGFFLLVEGGRIDHAHHESNAYRSLHEVISFDDAIAKAVELTSESDTLLVVTADHSHTFTLGGYTIRGNPLFGKARDDANPRTGLDGLPYTALNYGNGEGFQGGDSYSSPNEPVVREDLNTVDTGAKDYLQQSGVPLYSESHGGEDVAILARGPMSHLFHGVHQQSYVAHVMRYASCVGENKAHCENQPQTSVATEENVTFLGYNLNPSEAAGALYALFALELTFSIVIIGIGVYTVK

>Ciona savignyi_Alp1_Csareftig_194:404007_ENSCSAVT00000012048]

MHPLLKTSKKPAGRAGNPAENSPEFWHQSAAAELSEALSIEKRNKNVAKNVILFLGDGMGVASITTGRILRGQNAGSSGEETKLTMDTFPHFGFSKTYNVDAQVADSAGTATAYMTGVKTDLGVLGYNAAITPGVCNTTYGNEVSTVLEDSHKLGKAVGVVTTTRLNHATPASAYAHSAYRYWYGDADLTEEARINGCKDIAYQLYNALPNIQVALGGGRRYFKPNGIKDDEYPTLDNYRNDGQDLIQKWKNNMVDLGRNAQYVWNSAQFHGVDASQVDHLWGMFEPGDMNYEVDRSSDGAGEPSLAEMTSKAIEILQKDDDGFFLLVEGGRIDHSHHGGNAYKALHEVLAFDDAIAKGVEMTSESDTLIVVTADHSHTFTFGGYTLRGNPLFGLARDDANPKNALDGRPYTSLNYGNGKGFQGGDSFSTGGSVVRENLTNVNTKSKDYLQQTGVPKSSETHGGEDVAIFARGPMAHLFHGVHEQSYIAHVMRYASCVGSNKLHCENQPTEEVVVPPEIVSFLGYNLSKSSAEGALYALFALEMIFSLVILAFSAFYFKRK

>Ciona savignyi_Alp2_Csareftig_30:2,127,570_ENSCSAVT00000012778]

MTKIMLLYVSCILLGMTDQTLSVHPSENTKDFWFDQGTGSIEESVATFSRLKLNKAKNVVMMIGDGMNIATLTAGRVSIGQKSGASGEEHVTAIDQLRHAGIVKTYSVDYQTPDSAATATAILAGVKTNQGSLGVRAGATNCDTDKVTSVLEYAISQGKSTGIVTTTRVQQATPGAAYAHAINRMAYTDRRNTGGGRGCKDIATQLYEKRRGIQVILGGGRSHMRPYTVLDEEGYGKRGDRGDNKDLIQMWRNDMGPLNGVYTWNKRQFDMVDANSTDYLLGLFSDLDMNFVTNKPTNERYEEPSLEEMTRKAIQILSKNPNGYFLLVEGGMIDKGHHQGRARFALEEFHELDKAVAMVKSMTSEDDTLLIVTADHGHMFTFGGGSIRGNPVYGGSSKNLYVHGRPFTSILYGNGPGYSLYGGRRSSPGSWVARTRYYKQSSAVPLRAETHSAEDVPVYASGPWSHFVTGVHEQNYLAHIMMYSACLGE

>Ciona savignyi_Alp3_Csareftig_77:3,122,833_ENSCSAVT00000014770_BAA92180]

MSMICFVTMMCVCSLGTAQVLNERSAHFWELRNQAELDEAITKQSLNMRKAKNVIILVGDGMGITTITSGRILKGQVSGTSGEETKLAMDKFPFSGISKTYSVNKQVSDSASTATAFLTGVKTNDFILGLTGSAQRGICKGSIDENNIVTSVLIEAKNAGKSAGFVTTTRINHATPGATYAHTPERMWYGDADLTEEAKENGCKDVAQQFIDNSHLFTVALGGGRQYFRPNTTYDEEYPNNTNARLDGQDLIKQWKKIQLQQGNRAAYVWNATEFAAINPDNTDSLLGLFQPKDMHYEAHRSGDVAGEPSLSEMTAKAISLLKKNEEGYILLVEGGRIDHGHHEGNAYLALHDLVAFDDAIDTAVQMTSDDETMLIVTADHSHVFTIGGYSDRGNPIFGLAPNAIKPTLGDDNKTFTTLLYGNGPGYAFESCERENVTGVPTDVSTYLQQSAVPLSYETHGGEDVIIMSRGPMAHLFEGVHEQTYIAHVIRYATCIGKLSKDCNERFNPPKENGVILYFLGISMTSSKAVLALYVTLALLIITSIVAIAANIHIYRMVSSKPSEQMQKV

>Ciona savignyi_Alp4_Csareftig_14:183,717_ENSCSAVT00000016054]

MLHEISSLFLGDGMSITTITAGRIMKGQLDGKSGEEVKLAMDQFQFSGLSKTYSVDQQVSDSACTATAYLCGVKSDYSTLGLNGNVEYGVCSSQKGNEVESTLVKAYNAGKSTGIVTTRVVHASPAGAYAHSASRSWYGDADMPAEAKAEGCKDIAQQLMDSFDMITVALGGGRQYFRPNTVSDEEYDTRTYARKDGKDLLQDWETKYGKNAKFVWNQTDFDAIDPVTTEKLLGMFEQSDMKYEALRNTDKAGEPSLAEMTEKAIRMLQKNSKGYFLFVEGGRIDHGHHAGNAHLALRDLVAFDDAIKRAVDLTVDSDTLIVVTADHAHTLSMGGYSDRGNPILGLAPSSNNPYLVEDGKPYTTLLYANGPGFQGSSYIGAHERENLTDVDTESVSYLQQSSTPLSSETHGGDDVAILARGPMAHLFHGVHEQSYIPHAMWYASCIGDDTSHCSQTAKDVVAMPTATTTFDTAAEFLGINLTSTDTVVALYVQFVLVICLALFILLLLQGSKRSSSPKSPGVVGN

>Ciona savignyi_Alp5_Csareftig_58:1,653,485_ENSCSAVT00000016106]

KIYICIVCCYCVSALSTAHEENPEFWNDSAEQTLRSKYAQMKPQAKPAKNVILFIGDGMGLSTITAGRILKGQLNGQAGEETITALDKFTETGLSKVYCVDKQSPDSGSTATAILSGVKTKFGVVGLDGRTVFKKCSSAAGNEVESILLKSANLGKSTGIVTTTEVQHATPAAAYAHSASRGWYADNSMNSTTKSEGCKDISLQLLEMGRKINVILGGGRSYMRPKGIYDEEYYSRHAGVRGDGRDLIREWRNLQPSNRARYVWNRPGFNSVNAQNTDYLMGLFQPRDLRFDYDRRRDVGGEPSLEEMTEKAIQILQKNPMGYFLLVEGGKIDHAHHQNKANVALREFIAFEKAVQKAMDMTSTDETLIVVTADHGHVFTLGGYANRGNPIHGFARDHSDPEKAVDKLHYTSIAYANGPGYSLDSTTGKRTELTGETTRAMGYRFQTAIPLKGESHSSEDTIVFARGPMSQLFSGVYEQNYIAHAMMFASCVGK

>Silkmoth_Alp_NP_001036856[Bombyxmori]

MVRLLTLLSLLAAGSCGRRYGLDKDGYHRDDVGSRRSLQTPSTPAPELESEYWSRDAQSELGERAWYDGSSGYARNVVMFLGDGMSVATLTAARTLLGQRRGQTGEESRLSFEHFPTVGLSKTYCLDAQVADSACSASAYLCGAKANLGTIGVSGHVARHHCTAATDAAHQLASIASWALDADRDAGIVTTTRVTHASPAGAYAHTADRNWESDGDVTAGCSGHAQLDIAQQLVHARPGKDFKVILGGGRREFLPNTMTDEEGSRGRRYDGRNLISEWLTEKDSRGVTHEYVWNRQQLMEVSEELPEYLLGLFEPSHLQYHMEANHTTEPTLAELTEVAIKSLSRNKEGFFLFVEGGRIDHAHHENLAHLALDETIELSAAVEKARELLSEQDSLIVVTADHAHVMSISGYTQRGGDILGPSDALGDDGIPYMTLSYGNGPGYREPRGGQRVDPTRQDYRGSEYVYPAAVPLDSETHGGDDVAVFAWGPHHALFSGLYEQSHIPHRMAYAACIGPGKHACQHSPHSTT

>Budworm_Alp_ACP39714[HeliothisvirescensTobaccoBudworm]

MMSLYQCLLAVLCCAACARAHWFHPAATAGRAAATTRVETSANYWVQDAQAAIDARLAQVESVKKARNVIMFLGDGMSVPTLAAARTLLGQRQGNTGEETKLHFETFPTIGLVKTYCVDAQIADSACTATAYLCGVKNNYGAIGVDATVRRGDCQTASNTATHVESIAEWALADGRDVGIVTTTRITHASPAGTYAKTANRTWENDGEVSQMGFDAKDCPDIAHQLVHHHPGNKFKVILGGGRRAFLPNTVQDDEGSYGRRIDNRDLIKEWEDDKVARNVSHQYVWNREQLMSLNDDLPEYMLGLFGSSHMKYHMKSNPQSDPTLAELTEVAIRSLRRNEKGFFLFVEGGRIDHAHHDNLVELAPDETLEMDKAVATATQLLSEDDSLIVVTADHAHVMTINGSSGRGNDILGPSRDLGRDSMPYMTLSYTNGPGFRPHVNGIRPDVTAEPNFRTLDWESHVDVPLGDETHGGDDVAVFARGPHHSMFTGLYEQSQLPHLMAYAACIGPGRHACVSAAHLPSAHFLIALLALFTSILLR

>Beetle_Alp_EFA08950_TcasGA2_TC006657[Triboliumcastaneum]

MPKIISLIIYLAICAKTKGLNDKLMHPHPQTTPKIPYKSDENTAKFWNEQAQSTLKAHVSETINTNVAKNVILFMGDGMSVPTLAATRVYMGGENFELSFDKFPHTAISKTYCVNYQVPDSACTATAYLGGVKGNLGTVGVTAHVPWDDCQAMTNTSNNVPSIALWAQKQGKSTGFVTTARVTHASPAGVFGHSAQREWETDTDILNDGKDPKICTDLATQLVYHDGKNLNVIMGGGRYAFLPNDTKGEEGDYGYRSDGVNLIEGWKKLRKGKSKYIWNREQLLGLDPQDIDYVLGIFESDHCPFHLERDPAMDPTLTEMTESAIKILSKNPDGFFLFVEGARIDMGHHQAMAHLALEETAEFSRAVERAVSLTSDHDTLIVVTSDHAHTMSMGGYPFRGNDILGVVGDQAADGLAFTTLNYANGPGFRKAEDGARHDPSKDDLGDIEYRYPAEVPLDSETHGGDDVIIFARGPWAHLYRGVVEQNVIPHFMAYASCIGNGEENACKSKATLHGFATVLVVLMLAGIIL

>MosquitoAedes_XP_001659699[Aedesaegypti]

MYLLRMIAVFVVLLSSVLRASRGNPSQEFRLISASDYEETAQFWNIGAQLKLKEQLLKRKNFNKAKNVIFFLGDGMSIPTLAASRMYLGQKQGHSGEETQLSFEEFPDVGLVKTYCVDKQVADSACSATAYLCGVKANYATIGVTAAVKYNNCTTGNDPKNHVHSIMSWAQAAGKATGIVTTTRVTHASPAGTYAHVSNREHECDADILAQNADPNVCQDIASQLVRNNPGKNLKVILGGGRRKFTPNTEKDPSGKPGQRMDGVNLISEWYYSKPLGSARYVTNKQGLMGINFNETEYLMGLFNYDHMKYHMDSNPKEDPTLSDLTYAAIKTLEKNKAGFVLFVEGGKIDLAHHETKARKSLDETVQLSEAVQLATQYTNSEDTLILVTADHAHTMSMAGYSKRGHDILGVSGSSHDKGKSPYTTLSYANGPGGPSLTDGRRLNITEEMLTNKDFQYPKLVPLKYETHGGDDVALFAYGPWSHLFSGMYEQNVIPHIIGYAACIGSGLTACIG

>MosquitoCulex_XP_001843363[Culexquinquefasciatus]

MSTAIKIKNFSNTWWIQRNPEGNDTVIMHPPPVSLPTDEKETLRQKRLISASEYEKTAQFWNVGAQLKLKEQLLKRTNRNVAKNVIFFLGDGMSIPTLAASRMYLGQMHGHTGEETQLSFEEFPDVGLVKTYCVDKQVADSACSATAYLTGVKANYATIGVTAAVKYNNCADSSDPKNHVQSIMSWAQAAGKGTGIVTTTRVTHASPAGTYAHVANRDWESDADVEAKVEDSSQCQDIASQLVRNEPGKNFNVILGGGRRKFIPKNEKDVSGKPGQRMDSANLISEWFYSKPMGTARYVTNKRDLMGINFNETEYLMGLFNSDHLKYHMDSDLSEDPSLSDLTYAAIKSLEKHRNGFVLFIEGGKIDLAHHETKARKSLDETVQFSEAVLLATQLTNSDNTLIVVTADHSHVMSLAGYSKRGHDILDISDSISDVDKKAYTTLSYANGPGGPGPDEKGHRRNITQQMVKNKEFQYPKMVPLKYETHGGDDVALFAYGPWSHLFGGMYEQNVIPHLIGYAACIGDGLTACKR

>Zebrafish_alpl_ENSDARG00000015546_Chr11:28,845,674_zebrafish_alp_cDNA_YeYang1

MWECGCFLVLWSELSVVWHPWNVKIRKRRLIDNKMKVVQLLILSCLVWEGKTKVQFPEQEKRPDYWRDFAQRSLKDALKLQELNKNIAKNIILFLGDGMGVPTVTAARILKGQLSGQNGEETQLEMDKFPHVALSKTYNTNAQVPDSAGTATAFLCGVKANEGTVGVSAAAVRSQCNTTQGNEVTSILKWAKDAGKSVGIVTTTRVNHATPSAAYAHCVDRDWYSDADMPNEALQSGCKDIARQLFENIPDINVIMGGGRRSMYPKNTPDVEYPGDKKQNGTRKDGRNLVGEWIDRVKEKRGYYVWNKKDLLSLNPNNVDYLLGLFEPADLNYELERNTENDPSLTEMVDVAIKILKKNERGFFLLVEGGRIDHGHHEGKAKQALHEAVEMDRAITRAGLLTSEYDTLTVVTADHSHVFSFGGYTPRGNSIFGLAPTLSDVDQKPFTAILYGNGPGFKLVNGARENVSTVDYQQNNYQAQSAVPLRMETHGGEDVAIFSKGPMAHLLHGVQEQHYIPHVMAYAACIGQNKDHCRTNSGSSSYFSHISPALLFPLLVKWLLC

>Zebrafish_alpi_Dre22:41,820,800_NP_001014375_ENSDARG00000015273_intestinal-alkalinephosphatase[Daniorerio]

MCLVYGRAPGALLLLLLALVLNTSCSLDSAAEWEKDPAYWNDQARRTLQTALTLPLRVNRAKNIILFVGDGMGVSTVSAARILRGQMEGQSGEETILAMDTFPYLALSKTYCVDKQVADSASTATAYHCGVKANAKTVGLSAKAVAYECNTTFGNEVFSVLHRAKAQGKSVGIVTTTRVQHASPAAAYAHSVSRKWYSDADVPSEARRQGCKDIATQLVTNTDIDVILGGGRMYMTPKGTPDPEYSSSSHKGDRKDKKNLINVWLNARKGRNAQYVWNKEQFNAVDVKTTDCLMGLFEPKDMRFEVFRNRTRDPSIVDMTEKAIQILSKNPKGFFLFVEGGRIDHGHHDGVAKLALTETIMFDRAIQRASELTSESDTLTVVTADHSHVFTFGGKTPRGNPIFGLAPKQAEDDLPYTSILYANGPGYDHVNGTRGNVSVLDYYDEEYKQQAAVPLESETHGGEDVAIYAKGPMAHLFHGVKEQNYVAHAMAYAACLEPYTNCPLDLYSSAGWKTPLSLTLISLTGVFWLVLR

>Hydra_Alpl_XP_002166181alkalinephosphatase,liver/bone/kidney[Hydramagnipapillata]

MTFISGLLAFLKIISTLDAARINQQKDFNNNSLHKETDFNIQNSIIQQQSENQWFKNGVETIKKKLRVKKINKKAKNVILFIGDGMGPSTVTAARILDGQKKKKKGEENVLSWEEFDNVALSKTYNVDAQVPDSAGTATAFMSGVKTNMGTINVNENVTYKDCASSIGNEVLSLAQYAELAGLSTGVVTTTRLTHATPATVYASSASRNWEHDKTVNDSRCKDIASQLIDNKYGDGLEIAFGGGRCSFLPVTTKDHTATSYGLRGDGRNLLEEWEKKTNQNAKYEYVTNASQLKSLKPNEVDHLLGVFSYDHMNYSHYRLNGTDEPSLVEMVEFAVKVLSKNSKGYFLLVEGGRIDHGHHANKAFHALHDTVELNDAVHKAKEITDSDETLLIVTADHSHVFTIGGYQSRGNPIFGLVNNENDVALDKKRYSTLGYSNGPSGKINGKRKNITQADLNDPDSRYQSLVPLKDETHGGEDVGIYARGPQAHLVSGVVEQNYIFHVMDYALCLTEEKKYICKQEGLI

>SeaAnemone_Alp_XP_001637718[Nematostellavectensis]

MIRVDTLLVFAFCLVVLTALCSGDVPASQSNNQWFKDGVSTVKKHLLQRPNTKPAKNLIIFVGDGCDINTNTAGRILKGQLKGQVGEKGWLSYEEFPYTGLSKTYTTNRQGSDSAGTANAMFTGVKTRSAMIGVNEEVVTNKCETLTEDRKVDSILKLAEEAGMATGFITSMRLTHATPANLYAHSASRYWESDKEMVSRGYGNTSCKDMAQQLVDFQGSVGDGIEVVMGGGRRSFLPNTTRDPEHVNKTGERYDGRNLIQEWLNKREGSVYAWNKTQFDAVDPKTTKHFLGMFESSHMQFAIDRDEDVGGEPSIAEMVEKAVSILKNNDNGKGFFLAVEAGLIDIGHHNGIARQALNEVVNLEEGVAKAVEMTDKEDTLIISTADHGHVFTLTGYPDINSPIFGLMKQDGEVKLALNDNKPYTILGYMNGPGAVKGVRANLTGVDTGAKDFKQQALYIGYDYDEAHGSQDVGIYSRGPWAHLLTGVVEQNVIFHVMDHALCLSSSKQGTCSKPEARGGPSASAAMVTALFAPLVAALVTCLVLY
