# Supplementary material for: Dynamic Evolution of the LPS-Detoxifying Enzyme Intestinal Alkaline Phosphatase in Zebrafish and Other Vertebrates
Source: Front Immunol. 2012 Oct 12;3:314. doi: 10.3389/fimmu.2012.00314 (PMC3469785; doi:10.3389/fimmu.2012.00314)
Supplement: Supplementary Data sheet S3 — Alp sequences used to generate Figure 5. [file 35467_Guillemin_DataSheet3.DOCX]

>Human_ALPL_Hsa1:21,835,858_NP_000469.3_[Homosapiens]ENSG00000162551

MISPFLVLAIGTCLTNSLVPEKEKDPKYWRDQAQETLKYALELQKLNTNVAKNVIMFLGDGMGVSTVTAARILKGQLHHNPGEETRLEMDKFPFVALSKTYNTNAQVPDSAGTATAYLCGVKANEGTVGVSAATERSRCNTTQGNEVTSILRWAKDAGKSVGIVTTTRVNHATPSAAYAHSADRDWYSDNEMPPEALSQGCKDIAYQLMHNIRDIDVIMGGGRKYMYPKNKTDVEYESDEKARGTRLDGLDLVDTWKSFKPRYKHSHFIWNRTELLTLDPHNVDYLLGLFEPGDMQYELNRNNVTDPSLSEMVVVAIQILRKNPKGFFLLVEGGRIDHGHHEGKAKQALHEAVEMDRAIGQAGSLTSSEDTLTVVTADHSHVFTFGGYTPRGNSIFGLAPMLSDTDKKPFTAILYGNGPGYKVVGGERENVSMVDYAHNNYQAQSAVPLRHETHGGEDVAVFSKGPMAHLLHGVHEQNYVPHVMAYAACIGANLGHCAPASSAGSLAAGPLLLALALYPLSVLF

>Human_ALPP_Hsa2:233,243,244_NP_001623.3_[Homosapiens]_ENST00000392027

MLGPCMLLLLLLLGLRLQLSLGIIPVEEENPDFWNREAAEALGAAKKLQPAQTAAKNLIIFLGDGMGVSTVTAARILKGQKKDKLGPEIPLAMDRFPYVALSKTYNVDKHVPDSGATATAYLCGVKGNFQTIGLSAAARFNQCNTTRGNEVISVMNRAKKAGKSVGVVTTTRVQHASPAGTYAHTVNRNWYSDADVPASARQEGCQDIATQLISNMDIDVILGGGRKYMFRMGTPDPEYPDDYSQGGTRLDGKNLVQEWLAKRQGARYVWNRTELMQASLDPSVTHLMGLFEPGDMKYEIHRDSTLDPSLMEMTEAALRLLSRNPRGFFLFVEGGRIDHGHHESRAYRALTETIMFDDAIERAGQLTSEEDTLSLVTADHSHVFSFGGYPLRGSSIFGLAPGKARDRKAYTVLLYGNGPGYVLKDGARPDVTESESGSPEYRQQSAVPLDEETHAGEDVAVFARGPQAHLVHGVQEQTFIAHVMAFAACLEPYTACDLAPPAGTTDAAHPGRSVVPALLPLLAGTLLLLETATAP

>Human_ALPPL2_Hsa2:233,271,553_NP_112603.2_[Homosapiens]_ENST00000295453

MQGPWVLLLLGLRLQLSLGIIPVEEENPDFWNRQAAEALGAAKKLQPAQTAAKNLIIFLGDGMGVSTVTAARILKGQKKDKLGPETFLAMDRFPYVALSKTYSVDKHVPDSGATATAYLCGVKGNFQTIGLSAAARFNQCNTTRGNEVISVMNRAKKAGKSVGVVTTTRVQHASPAGAYAHTVNRNWYSDADVPASARQEGCQDIATQLISNMDIDVILGGGRKYMFPMGTPDPEYPDDYSQGGTRLDGKNLVQEWLAKHQGARYVWNRTELLQASLDPSVTHLMGLFEPGDMKYEIHRDSTLDPSLMEMTEAALLLLSRNPRGFFLFVEGGRIDHGHHESRAYRALTETIMFDDAIERAGQLTSEEDTLSLVTADHSHVFSFGGYPLRGSSIFGLAPGKARDRKAYTVLLYGNGPGYVLKDGARPDVTESESGSPEYRQQSAVPLDGETHAGEDVAVFARGPQAHLVHGVQEQTFIAHVMAFAACLEPYTACDLAPRAGTTDAAHPGPSVVPALLPLLAGTLLLLGTATAP

>Human_ALPI_Hsa2:233,320,833_NP_001622.2_[Homosapiens]_ENSG00000163295

MQGPWVLLLLGLRLQLSLGVIPAEEENPAFWNRQAAEALDAAKKLQPIQKVAKNLILFLGDGLGVPTVTATRILKGQKNGKLGPETPLAMDRFPYLALSKTYNVDRQVPDSAATATAYLCGVKANFQTIGLSAAARFNQCNTTRGNEVISVMNRAKQAGKSVGVVTTTRVQHASPAGTYAHTVNRNWYSDADMPASARQEGCQDIATQLISNMDIDVILGGGRKYMFPMGTPDPEYPADASQNGIRLDGKNLVQEWLAKHQGAWYVWNRTELMQASLDQSVTHLMGLFEPGDTKYEIHRDPTLDPSLMEMTEAALRLLSRNPRGFYLFVEGGRIDHGHHEGVAYQALTEAVMFDDAIERAGQLTSEEDTLTLVTADHSHVFSFGGYTLRGSSIFGLAPSKAQDSKAYTSILYGNGPGYVFNSGVRPDVNESESGSPDYQQQAAVPLSSETHGGEDVAVFARGPQAHLVHGVQEQSFVAHVMAFAACLEPYTACDLAPPACTTDAAHPVAASLPLLAGTLLLLGASAAP

>Mouse_Alppl2_Mmu1:88,983,265_ENSMUST00000027455[Mouse

MWGACLLLLGLSLQVCPSVIPVEEENPAFWNRKAAEALDAAKKLKPIQTSAKNLVILMGDGMGVSTVTATRILKGQQQGHLGPETQLAMDRFPHMALSKTYNTDKQIPDSAGTGTAFLCGVKTNMKVIGLSAAARFNQCNTTWGNEVVSVMHRAKKAGKSVGVVTTTSVQHASPAGTYAHTVNRGWYSDAQMPASALQDGCKDISTQLISNMDIDVILGGGRKFMFPKGTPDQEYPTDTKQAGTRLDGRNLVQEWLAKHQGARYVWNRSELIQASLNRSVTHLMGLFEPNDMKYEIHRDPAQDPSLAEMTEVAVRMLSRNPKGFYLFVEGGRIDHGHHETVAYRALTEAVMFDSAVDKADKLTSEQDTMILVTADHSHVFSFGGYTQRGASIFGLAPFKAEDGKSFTSILYGNGPGYKLHNGARADVTEEESSNPTYQQQAAVPLSSETHSGEDVAIFARGPQAHLVHGVQEQNYIAHVMAFAACLEPYTDCGLASPAGQSSAVSPGYMSTLLCLLAGKMLMLMAAAEP

>Mouse_Alpi_Mmu1:88,994,579_ENSMUST00000113270[mouse

MQGDWVLLLFLGLRIHLSFGIIPAEEENPAFWNKKAAEALDAAKKLQPIQTSAKNLIIFLGDGMGVPTVTATRILKGQLEGHLGPETPLAMDLFPYMALSKTYNVDRQVPDSAGTATAYLCGVKANYKTIGLSAAARLDQCNTTFGNEVFSVMYRAKKAGKSVGVVTTTRVQHASPAGTYAHTVNRNWYSDAEMPASALQDGCKDIATQLISNMDIDVILGGGRKFMFPKGTPDPEYPSDSNQSGTRLDDQNLVQTWLSKHQGARYVWNRSELIQASQDPAVTHLMGLFEPTEMKYDANRNPSVDPSLAEMTEVAVRMLSRNPQGFYLFVEGGRIDQGHHAGTAYLALTEAVMFDSAIEKASQLTNEKDTLILITADHSHVFAFGGYTLRGTSIFGLAPLKALDDKSYTSILYGNGPGYELKSGNRPNVTEAQSVDPNYKQQAAVPLSSETHGGEDVAIFARGPQAHLVHGVQEQNYIAHVMAFAGCLEPYTDCGLAPPAGQSPVITPGQATTTNNAAGQATTTNNAAGQATVLLSLQLLVSMLLLVGTAMVVS

>Mouse_Akp3_Mmu1:89,021,583_ENSMUST00000044878[mouse

MQGTWVLLLLGLRLQLSLSVIPVEEENPAFWNKKAAEALDAAKKLQPIQTSAKNLIIFLGDGMGVPTVTATRILKGQLEGHLGPETPLAMDRFPYMALSKTYSVDRQVPDSASTATAYLCGVKTNYKTIGVSAAARFDQCNTTFGNEVFSVMYRAKKAGKSVGVVTTTRVQHASPSGTYVHTVNRNWYGDADMPASALREGCKDIATQLISNMDINVILGGGRKYMFPAGTPDPEYPNDANETGTRLDGRNLVQEWLSKHQGSQYVWNREQLIQKAQDPSVTYLMGLFEPVDTKFDIQRDPLMDPSLKDMTEAAVKVLSRNPKGFYLFVEGGRIDRGHHLGTAYLALTEAVMFDLAIERASQLTSERDTLTIVTADHSHVFSFGGYTLRGTSIFGLAPLNALDGKPYTSILYGNGPGYVGTGERPNVTAAESSGSSYRQQAAVPVKSETHGGEDVAIFARGPQAHLLHGVQEQNYIAHVMAFAGCLEPYTDCGLAPPADESQTTTTTRQTTITTTTTTTTTTTTPVHNSARSLGPATAPLALALLAGMLMLLLGAPAES

>Mouse_Alpl_Mmu4:137,297,648_ENSMUST00000030551

MISPFLVLAIGTCLTNSFVPEKERDPSYWRQQAQETLKNALKLQKLNTNVAKNVIMFLGDGMGVSTVTAARILKGQLHHNTGEETRLEMDKFPFVALSKTYNTNAQVPDSAGTATAYLCGVKANEGTVGVSAATERTRCNTTQGNEVTSILRWAKDAGKSVGIVTTTRVNHATPSAAYAHSADRDWYSDNEMPPEALSQGCKDIAYQLMHNIKDIDVIMGGGRKYMYPKNRTDVEYELDEKARGTRLDGLDLISIWKSFKPRHKHSHYVWNRTELLALDPSRVDYLLGLFEPGDMQYELNRNNLTDPSLSEMVEVALRILTKNLKGFFLLVEGGRIDHGHHEGKAKQALHEAVEMDQAIGKAGAMTSQKDTLTVVTADHSHVFTFGGYTPRGNSIFGLAPMVSDTDKKPFTAILYGNGPGYKVVDGERENVSMVDYAHNNYQAQSAVPLRHETHGGEDVAVFAKGPMAHLLHGVHEQNYIPHVMAYASCIGANLDHCAWAGSGSAPSPGALLLPLAVLSLRTLF

>Macrobat_Alpi_scaf_14928:23,613_ENSPVAT00000014879_megabat

MQGAWVLLLLLGLGLRLSLGVIPVEEEDPAFWNRQAAQALDTAKKLQPIQTAAKNLIIFLGDGMGVSTVTTARILKGQENDKLGPETPLAMDGFPYLALSKTYNVDRNVPDSAGTATAFLCGVKTNYKTIGVSAAARFNQCNSTWGNEVISVMNRAKKAGKSVGVVTTTRVQHASPAGAYAHTVNRNWYSDADMPARALKEGCQDIATQLVSNMDIDVILGGGRQYMFPNGTPDPEYPDDAARSGVRLDSRNLVQEWQAKRQGARYVWNRTALIQASQDPSVTHLMGLFEPKDMKYELYRDPQQDPSLREMTEAALRLLSRNAQGFYLFVEGGRIDHGHHDSKAQLALTETVSFDDAIDRAGQLTSEKDTLTLVTADHSHVFTFGGYPLRGSSVFGLAPEKASDGKAYTSLLYGNGPGFSLSEGSRSNVSDSQSTNPEYRQQAAVPVASETHGGEDVAVFARGPQAHLVHGVQEQSFVAHVMAFAACLEPYTDCGLTPPAGPTDAAHPGRAAGPSLALLAGALLLLPLLGAVAL

>Macrobat_Alpl_Scaf_2562:33,207_ENSPVAT00000000629_megabat

MISPFLVLAISTCVTTSLVPEKEKDPKYWRDQAQQTLKNALKLQNLNTNVAKNVILFLGDGMGVSTVTAARILKGQLHHSPGEETRLEDKFPYVTYNTNAQVPDSAGTATAYLCGVKANEGTVGVSAATQRSHCNTTQGNEVTSILRWAKDAGKSVGIVTTTRVNHATPSAAYAHSADRDWYSDNEMPPEALNQGCKDIAYQLMHNIRDIEVIMGGGRKYMFPKNRTDVEYEIDEKARGTRLDGLNLIDIWKSFKPRHKHSHYIWNRTELLALDPNTVDYLLGRIDHGHHEGKAKQALHEAVEMDQAIEQAGTMTSVEDTITVVTADHSHVFTFGGYTPRGNSIFGLAPMIDTYKKPFTAILYGNGPGYKVVGGERENVSMVDYAHNNYQAQSAVPLRHETHGGEDVAVFAKGPMAHLLHGVHEQNYIPHVMAYAACIGANRDHCASVSSLGSSSPGSLLLPLALLPLGILF

>Microbat_Alpi_ScafGL429770:27,688,525_ENSMLUT00000007631_microbat

MQGAHLLVLLLLLGLRIQLSLGFIPAEEEDPAFWNHQAAQALDTAKKLQPIQTAAKNLILFLGDGMGVPTVSAARILKAQMNGKLGPETPLAMDKFPFLALAKTYNVDRQVPDSAGTATAYLCGVKTNYRTIGVSAAARYNQCNTTHGNEVISVMNRAKKAGKSVGVVTSTRVQHASPSGTYAHTVNREWYSDADMPTQALKDGCRDIATQLITNMDIDVILGGGRKYMFPKGTPDPEYPGDASKSGVRLDMRNLVQEWQAKHEGARYVWNRTALIQASQDSSVTHLMGLFEPGDMNYEAKRDTQQDPSLKEMTEAALRLLRRNPRGFYLFVEGGRIDQGHHAGRAYYALTDAVAFDDAIDRASQLTSEKDTLTLVTADHSHVFTFGGYTLRGSSIFGLAPEPASDGKTYTSILYGNGPGFAINGGSRPNVSDSQSGDPNYKQQAAVPLSSETHGGEDVAVFARGPQAHLMHGVQEQSVVAHVMAFAACLEPYTACGLEPPTSTSIPDAAHPGLSTGPSLALVIGALWLILLGLLW

>Microbat_Alpl_ScafGL429895:1,850,469_ENSMLUT00000001966_microbat

MISPFLVLAIGTCLTTSLVPEKEKDPKYWRDQAQQTLKNALSLQNLNTNVAKNTIMFLGDGMGVSTVTAARILKGQLHHNTGEETRLEMDKFPHVALSKTYNTNAQVPDSAGTATAYLCGVKANEGTVGVSAATQRSHCNTTQGNEVTSILRWAKDAGKSVGIVTTTRVNHATPSATYAHSADRDWYSDNEMPPEALSQGCKDIAYQLMHNIRDIEVIMGGGRKYMFPKNRTDVEYELDEKARGTRLDGLNLIDIWKSFKPRHKHSHYVWNRTELLALNPNTVDYLLGLFEPGDMQYELNRNNVTDPSLSEMVEVAIKILSKNPKGFFLLVEGGRIDHGHHEGKAKQALHEAVEMDQAIGLAGTMTSLDDTLTVVTADHSHVFTFGGYTPRGNSIFGLAPMLSDTDKKPFTSILYGNGPGYKVVGGERENVSMVDYAHNNYQAQSAVPLRHETHGGEDVAVFAKGPMAHLLHGVHEQNYIPHVMAYAACIGANRDHCASASSSGSPSPGPLVLLLALLPLGILF

>Microbat_Alpi2b_ScafGL429770:27,671,295_ENSMLUT00000026875+Johnbuilt_microbat

MQGAHVLLLLLLLGLRIQLSIGFIPAEEEDPAFWNHQAAQALDTAKKLQPIQTAAKNLILFLGDGMGVSTVTATRILKGQMNGKLGPETSLAMDKFPFLALAKGKAPGGLGPELGAQGLVMIWVSVPERSGCLCPQTYNVDRQVPDSAGTATAYLCGVKTNFLIIGVSAAARYNQCNTTHGNEVISVMNRAKKAGGLGASLVGRGRAQRAQWLTLTPPSPSGRHPQGSQWGVVTSTRVQHASPAGTYAHTVNRNWYSDADMPAQALKDGCRDIATQLITNMDIDVGHEGNGDLSRGGGPHAGHSPRHPTA*APGPGAWQCLWALGRWCRRYRQARPCHRPGGGGTRWFVQGAKGQAGPKLTCPTPLAPGDPGWRPQVHVPRDPKSMVAHVMAFAACLEPYTACGLTPPA

>Panda_Alpi_AmelGL192569.1:1,591,050_ENSAMET00000018233[Ailuropodamelanoleucapanda]

SMARMLLLLLLGLRPQLVLGIIPVEEEDADFWNRQAAQALDTAKKLEPIQTAAKNLILFLGDGMGVPTVTATRILKGQLNDKLGPETPLAMDHFPYLALSKTYNVDRQVPDSAATATAYLCGVKANYQTIGVSAAARWNQCNTTRGNEVTSVMNRAKKAGKSVGVVTTTRVQHASPAGTYAHVVNRNWYSDADMPAKAREEGCQDIAQQLISNMDIDVILGGGRKYMFPQGTPDPEYPSDAGQDGVRLDGRNLVQEWQAKYQGARYVWNRTALIEASQDPSVTHLMGLFEPGDTKYEVRRDTTQDPSLMEMTEVAVRLLSRNPRGFYLFVEGGRIDHGHHDGTAYLALTEAVMFDSAIDKASQITSEKDTLTLVTADHSHVFSFGGYTLRGSSIFGLAPSIAQDNKTYTSILYGNGPGGSFALLGISRPNVSDS

>Panda_Alpl_AmelGL192679.1:243,455_ENSAMET00000015653[Ailuropodamelanoleucapanda]

MISLFLVLAIGTCLTNSFVPEKEKDAKYWRDQAQQTLKNALRLQKLNTNVAKNVIIFLGDGMGVSTVTAARILKGQLQHNPGEETSLEMDKFPYVALSKTYNTNAQVPDSAGTATAFLCGVKTNEGTIGVSAATQRSQCSTAKGNEVTSILHWAKDAGKSVGIVTTTRVNHATPSAAYAHSAEREWYSDNEMSPEALRQGCKDIAYQLMHNIKDIEVIMGGGRKYMFPKNRTDVEYEMDEKSRGTRLDGLNLIDTWKSFKPKHKHSHYVWNRTELLTLDPHTVDYLLGLFEPGDLQYELNRNNVTDPSLSEMVEVAVKILSKNPKGFFLLVEGGRIDHGHHESKAKLALHETVEMDRAIEKAGAMTSLEDTLTIVTADHSHVFTFGGYTPRGNSIFGLAPMVSDTDNKPFTAILYGNGPGYKVIGGERENIYKVDYAHSNYQAQAAVPLRYETHGGEDVAVFAKGPMAHLLHGVHEQNYIAHVMGYAACVGANQDHCASASSAGGPSPGPLLLLLTLLPLGTLF

>Dog_Alpp_Cfam25:47,107,892_ENSCAFG00000011195

LPRPCHPTAMQGARLRPRLALGIIPAEEEDPAFWNRQAAQALDAAKKLQPIQTAAKNLILFLGDGMGVPTVTATRILKGQINDNLGPETPLAMDQFPYLALSKTYNVDRQVPDSAGTATAYLCGVKANYQTIGVSAAARFNQCNTTRGNEVISVMNRAKKAGKSVGVVTTTRVQHASPAGTYAHVVNRNWYSDANMPAKALEDGCQDIAQQLISNMEIDVILGGGRKYMFPKGTPDPEYPTDAKQNGIRLDGRNLVQEWQAKYQGARYVWNRTALIQASQDASVTHLMGLFEPGDTKYDVHRDGIQDPSLMEMTEAALRLLSRNPKGFYLFVEGGRIDHGHHDGTAYLALTEAVMFDSAIDKAGQLTSERDTLTLVTADHSHVFSFGGYTLRGSSIFGLAPSMAKDNKTYTSILYGNGPGFALSGVPRPNFSDAESRDPAYKPQAAVPLDSETHGGEDVAVFARGPQAHLVHGVQEQSFVAQVMTFAACLEPYPATRPAGPLLAEEAAGRGAVPPTLGTSWGPSLLLHTGHAD

>Dog_Alpl_Cfam2:80,454,145_ENSCAFT00000023578_NP_001184066_dog

TEKEKDPKYWRDQAQQTLKYALRLQNLNTNVAKNVIMFLGDGMGVSTVTATRILKGQLHHNPGEETRLEMDKFPYVALSKTYNTNAQVPDSAGTATAYLCGVKANEGTVGVSAATQRTQCNTTQGNEVTSILRWAKDAGKSVGIVTTTRVNHATPSAAYAHSADRDWYSDNEMPPEALSQGCKDIAYQLMHNVKDIEASVIMGGGRKYMFPKNRTDVEYEMDEKSRGTRLDGLNLIDIWKNFKPRHKHSHYVWNRTELLALDPYTVDYLLGLFEPGDMQYELNRNNVTDPSLSEMVEIAIKILSKNPRGFFLLVEGGRIDHGHHEGKAKQALHEAVEMDRAIGKAGVMTSLEDTLTVVTADHSHVFTFGGYTPRGNSIFGLAPMVSDTDKKPFTAILYGNGPGYKVVGGERENVSMVDYAHNNYQAQSAVPLRHETHGGEDVAVFAKGPMAHLLHGVHEQNYIPHVMAYAACIGANQDHCASASSAGGPSSPGPLLLLLALLPVGILF

>Cow_Alpi2_Btau2:120,845,481_ENSBTAT00000022731

MQGACVLLLLGLRLQLSLGLVPVEEEDPAFWNRQAAQALDVAKKLQPIQTAAKNVILFLGDGMGVSTVTAARILKGQMAGKPGPETPLAMDQFPYLALSKTYNVDRDVPDSAGTTTAYLCGVKTRMKVIGVSAAAQFNQCNTTYGNEVTSVMNRAKKAGKSVGVVTTTTVQHASPAGAYAHTVNRNWYSDANMPAEAKREGCQDIATQLVYNMDIDVILGGGRIYMFPEGTPDPEYPGNTKQNGVRKDKRNLVQEWQAKHQGAQYVWNRTALLQAANDSSVTHLMGLFEPGDMTYDIHRDHIKDPSLEEMTEAAVRVLSRNPRGFFLFVEGGRIDHGHHESIAYRALTEAVMFDNAIAKASQLTSEADTLTLVTADHSHVFTFGGYPLRGTSIFGLADGKAKDGKSYTTLLYGNGPGHRLVMGSRPDVNEKESMDPEYQQQSAVPLWGETHAGEDVAVFARGPWAHLVHGVQEQTFVAHVMAFAACVEPYTTDCHPHPHSGPSDTAHQAACPSSLALLAGALLLLLVPTLH

>Cow_Alpi4_Btau2:120,869,240_ENSBTAT00000022269

MQGACVMLLLGLQLQLSLGLVPVEEEDPAFWNRQAAQALDVAKKLQPIQTAAKNVILFLGDGMGVSTVTAARILKGQMAGKPGPETPLAMDQFPYLALSKTYNVDRDVPDSAGTTTAYLCGVKTNMRTIGVSAAARFDQCNTTRGNEVTSVINRAKKAGKSVGVVTTTRVQDASPAGAYAHTVNRDWFSDADLPPDAQTYGCLDIATQLVYNMDIDVILGGGRKYMFPAGTPDPEYPDDNGVRKDKRNLVQEWQAKYQGAQYVWNRTELLKAADDSNVTHLMGLFQPGEMAYEIFRDHTTDPSLEEMTEAALRVLSRNPRGFFLFVEGGRIDHGHHANTAYWALNETIMFDNAIAKASQLTSEADTLTLVTADHSHVFTFGGYPLRGTSIFGLADGKAKDGKSYTSLLYGNGPGYRLDVGPRPDVNEKESTDPEYQQQAA

>Cow_Alpi5_Btau2:120,904,601_F1N2M5_BOVIN_ENSBTAT00000043359

MQGACVLLLLGLQLQLSLGLIPVEEEDPAFWNCQAAQALDVAKKLQPIQTAAKNVILFLGDGMGVPTVTATRILKGQMNGKLGPETPLAMDQFPYVALSKTYNVDRQVPDSAGTATAYLCGVKGNYKTIGVSAAARYNQCNTTSGNEVTSVMNRAKKAGKAVGVVTTSRVQHASPAGAYAHTVNRNWYSDADLPADAQMNGCQDIATQLVYNMDIDVILGGGRMYMFPEGTPDPEYPYDVNQTGVRKDKRNLVQEWQAKHQGAQYVWNRTALLQAADDSSVTHLMGLFEPADMKYNVQQDHTKDPTLQEMTEVALRVLSRNPRGFYLFVEGGRIDHGHHEGKAYMALTDTVMFDNAIAKANELTSELDTLILVTADHSHVFSFGGYTLRGTSIFGLAPSKALDSKSYTSILYGNGPGYALGGGSRPDVNDSTSEDPSYQQQAAVPLASETHGGEDVAVFARGPQAHLVHGVQEETFVAHIMAFAGCVEPYTDCNLPAPTTATSIPDAAHLAASPPPLALLAGAMLLLLAPTLY

>Cow_Alpi6_Btau2:120,910,019_F1N6T5_BOVIN_ENSBTAT00000005937

MQGACVLLLLGLHLQLSLGLVPVEEEDPAFWNRQAAQALDVAKKLQPIQTAAKNVILFLGDGMGVPTVTATRILKGQMNGKLGPETPLAMDQFPYVALSKTYNVDRQVPDSAGTATAYLCGVKGNYRTIGVSAAARYNQCKTTRGNEVTSVMNRAKKAGKSVGVVTTTRVQHASPAGAYAHTVNRNWYSDADLPADAQMNGCQDIAAQLVNNMDIDVILGGGRKYMFPVGTPDPEYPDDASVNGVRKDKQNLVQAWQAKHQGAQYVWNRTALLQAADDSSVTHLMGLFEPADMKYNVQQDHTKDPTLQEMTEVALRVLSRNPRGFYLFVEGGRIDHGHHDDKAYMALTEAVMFDNAIAKANELTSELDTLILVTADHSHVFSFGGYTLRGTSIFGLAPSKALDSKSYTSILYGNGPGYALGGGSRPDVNDSTSEDPSYQQQAAVPLASETHGGEDVAVFARGPQAHLVHGVQEETFVAHIMAFAGCVEPYTDCNLPAPTTATSIPDAAHLAASPPPLALLAGAMLLLLAPTLY

>Cow_Alpl_Btau2:131,791,680_PPBT_BOVIN_ENSBTAT00000011783

MISPFLLLAIGTCFASSLVPEKEKDPKYWRDQAQQTLKNALRLQTLNTNVAKNVIMFLGDGMGVSTVTAARILKGQLHHSPGEETKLEMDKFPYVALSKTYNTNAQVPDSAGTATAYLCGVKANEGTVGVSAATQRSQCNTTQGNEVTSILRWAKDAGKSVGIVTTTRVNHATPSASYAHSADRDWYSDNEMPPEALSQGCKDIAYQLMHNIKDIEVIMGGGRKYMFPKNRTDVEYELDEKARGTRLDGLNLIDIWKSFKPKHKHSHYVWNRTDLLALDPHSVDYLLGLFEPGDMQYELNRNNATDPSLSEMVEMAIRILNKNPKGFFLLVEGGRIDHGHHEGKAKQALHEAVEMDQAIGQAGAMTSVEDTLTVVTADHSHVFTFGGYTPRGNSIFGLAPMVSDTDKKPFTAILYGNGPGYKVVGGERENVSMVDYAHNNYQAQSAVPLRHETHGGEDVAVFAKGPMAHLLHGVHEQNYIPHVMAYAACIGANRDHCASASSSGSPSPGPLLLLLALLPLGSLF

>Pig_Alpl_GL893988.2:13,511_ENSSSCT00000032355

MISLFLVLAIGSCLTNSLVPDSSSDWESRAQQTERGTLCLNTMSLCWVEGTSVGNVIMFLGDGMGVSTVTAARILKGQLHHKPGEETRLEMDKFPYVALSKTYNTNAQVPDSAGTATAYLCGVKANEGAVGVSAATQRTQCNTTQGNEVTSILRWAKDAGKSVGIVTTTRVNHATPSAAYAHSAETWCSDGSLLPVLKQSRRALTFNSLRRGQELKVIMGGGRKYMFPKNRTDVESRCQEGERGPTSLHWTGVIDKPPLRKQHSHYIWNRTELLALDPHTVDYLLGLFEPGDMQYELNRNNVTDPSLSEMVEMAIRILIKNPKGFFLLVEDFGRQDHGHHEGKAKQALHEAVEMDRAIEQAGSMTSVEDTLTVVTADHSHVFTFGGYTPRGNSIFGLAPMVSDTDKKPFTAILYGRENVSMVDYAHDNYQAQSAVPLRHETHGGLLHGVHEQNYIPHVMAYAACIGANRDHCASASSSGSPSPGPLLLLLAFLPLGILF

>Pig_Alpi_Suscr15:146,783,645_ENSSSCT00000028730_ENSSSCG00000028816

MQGGWVLLLLGLRLPLSLGFIPVEEEDPAFWNRQAAQALDVAKKLQPIQTAAKNLILFLGDGMGVSTVTATRILKGQMNGKPGPETPLAMDRFPYLALSKTYNVDRQVPDSAGTTTAYLCGVKTNMKVIGVSAAARYDQCNTTQGNEVISVMNRAKKAGKSVGVVTTTRVQHASPAGAYAHTVNRNWYSDADLPAEAKKNGCQDISTQLVYNMDIDVILGGGRKYMFPEGTPDPEYPDNPRQNGVRKDKRNLVQEWQAKHQGARYVWNRTALIQASQDPSLTHLMGLFEPGDMKYEKERDLSRDPSLVEMTEVALRLLSRNPRGFFLFVEGGRIDHGHHEGIAYRALIETVVFDTAIDKAGQLTSEEDTLTLVTADHSHVFTYGGYPLRGSSVFGLADGKASDGKAYTSILYGNGPGYKLSEGARPDVDETKSRDPAYVQQAAVPLGAETHGGEDVAVFARGPRAHLVHGVQEQSFVAHVMAFAACLEPYTSDCDLPPPSGPTAAGHPGPAACTSLLALLAGALLLLLAPALH

>Pig_Alpi1_Sscr15:146,868,803_ENSSSCT00000017732

MQGGWVLLLLGLRLPLSLGFIPVEEEDPAFWNRQAAQALDVAKKLQPIQTAAKNLILFLGDGMGVPTVTATRILKGQMNGKPGPETPLAMDRFPYLALSKTYNVDRQVPDSAGTATAYLCGVKANYQTIGLSAAARYNQCNTTQGNEVISVMNRAKKAGKSVGVVTTTRVQHASPAGTYAHTVNRNWYSDVNLPAEAQAQGCQDIATQLVYNMDIDVILGGGRKYMFPEGTPDPEYPDDASQNGVRKDKRNLVQEWQDKYQGARYVWNRRTFLQAAQDPSVTHLMGLFEPADMKFEAERDVSMDPSLEEMTEMALQMLSRNPRGFYLFVEGGRIDHGHHEGIAYMALTESVMFDTAIDKAGQLTSEKDTLILVTADHSHVFSFGGYTLRGSSVFGLAPSKALDLKPYTSILYGNGPGYALNGSSRPSVTGSEISDRMYRQQAAVPLESETHGGEDVAVFARGPQAHLVHGVQEQSFVAHVMAFAACLEPYTDCNLRPPEGLSNAAHPRPVACPPSLLLLLAGALLLLLMPALH

>Opossum_Alpl_Mdom4:356,739,559_ENSMODT00000020333_opossum

GSTMFLLLLCLMAGTCLSIVPEKEKNPWYWREQAQRTLQHSLKLQNLNTNVARNVILFLGDGMGVSTVTAARILKGQLHHKLGEDFQLEMDKFPFVALSKTYNTNAQVPDSAGTATAYLCGVKSNEGTVGVSAAVTRSQCNTTKGNEVTSILRWAKDAGKSVGIVTTTRVNHATPSAAYAHSADRDWYSDNEMPPEALNQGCKDIAYQLMHNIPDIEVIMGGGRKYMFPKNTTDVEYGDDPKARGTRLDGQVLIDVWKGKKPRNKNAHYVWNRMQLMNLDLNNVDYLLGLFEPSDMVYELNRNNKTDPSLSEMVEVAIKILRKNPKGFFLLVEGGRIDHGHHEGKAKQALHEAVEMDRAIGLAGTLTSLEDTLTVVTADHSHVFTFGGYTPRGNSIFGLAPVVSDTDNKPYTSILYGNGPGYKVVAGQRENVSTVDYAHDNYQAQSAVPLSYETHGGEDVAVFAKGPMAHLLHGVQEQNYIPHVMAYASCIGSIMDHCQSHPSAGLAPQPALFSGLLTLLLLLSLLF

>Opossum_Alpi_Mdom2:537,202,874_ENSMODT00000003218

MQGIFALFLVGLYFHTAKGEIPEEEENPSFWNRQAAEAIKTAQQLQPIQTAAKNLILFLGDGMGVPTVTATRILKGKLSGNLGPETPLAMDRFPYVALSKTYNVDRQVPDSAGTATAYLCGVKGNYKTIGLSAAARVDQCNSTFGNEVYSVLQRAKKAGKSVGVVTTTRVQHASPSGTYAHVVNRDWYSDANMPADALRSGCKDIASQLISNVDIDVILGGGRKYMFPVGTPDPEYPNLSSQNGIRRDDRNLVQEWLDARQGAKYVWDRQELLNAAENLEVTHIMGLFEPGDTKYDLQRNTSVDPSLMEMTEVAIKILRRNPKGYYLFVEGGRIDHGHHDGNAHLALTEAVMFDSTIDKAVQMTNEKDTLIAVTADHSHVFSFGGYTYRGSSIFGLAPSKAQDNKAYTSILYGNGPGYVNGTRPDVKESVSEDVSYKQQAAVPLSSETHGGEDVAIFARGPQAHLFHGVQEETYVAHVMAFAGCLEPYENCGLSWPNSSLQTSVS

>Rat_Alppl2_Rno9:85,873,714_ENSRNOT00000033487_ENSRNOG00000042889

WVLLLLLLGLRLQLSFGVIPVEEENPAFWNQKAKEALDVAKKLKPIRTSAKNLIIFLGDGMGVPTVTATRILKGQLGGHLGPETPLAMDHFPFTALSKTYNVDRQVPDSAGTATAYLCGVKANYKTIGVSAAARFNQCNSTFGNEVFSVMHRAKKAGKSVGVVTTTSVQHASPAGTYAHTVNCDWYSDAHMATAALQEGCKDIAMQLISNMDIDVILGGGRKFMFPKGTPDPEYSGNRAQSGTRLDGQNLVQKWLAKHQGARYVWNRTELIQASQDSAVTHLMGLFEPNDMKYDIYRDPTQDPSLAEMTEVAVHLLSRNPKGFYLFVEGLGASFNALDGRPFTSILYGNGPGYKLEKGTRPDVTDKESRDPKYRQQAAVPLSSETHSGEDVAIFARGPQAHLVHGVQEQNYIAHVMAFAGCLEPYTDCGLAPPAGRSSAVSPGYISTLLCLLAGTMLMLLVSAEP

>Rat_Alpi2_Rno9:85,885,336_LOC100359675ENSRNOT00000025978_ENSRNOG00000033672

MWGACLLLLGLSLQMSLSLVPVEEENPAFWNRKAAEALEAAKKLQPIQTSANNLIILMGDGMGVSTVTATRILKGQQQGHLGPETPLAMDRFPHMALSKTYNTDKQVPDSAGTGTAFLCGVKTNMKVIGLSAAARFNQCNTTWGNEVVSVMHRAKKAGKSVGVVTTTSVQHASPAGTYAHTVNRNWYSDAHMSTAALQEGCKDIAMQLISNTDIDVILGGGRKFMFPKGTPDQEYPTDPQQAGTRLDGRNLVQEWLAKHQGAQYVWNRTELIQASLNRSVTHLMGLFEPNDMKYNIDRDPTQDPSLAEMTEVAVHMLSRNPKGFYLFVEGGRIDHGHHEAIAYRALTEAVMFDSAVDKADKLTSEKDTMIIVTADHSHVFSFGGYTQREASIFGLAPFKAGDGKSFTSILYGNGPGYKLNNGIRADVTEEESSNPTYQQQAAVPLSSETHSGEDVAIFARGPQAHLVHGVQEQNYIAHVMAFAGCLEPYTDCGLAPPAGRSSAVSPGYISTLLCLLAGTMLMLMVSAEP

>Rat_Alpi_Rno9:85,892,887-ENSRNOT00000026190_ENSRNOG00000030020

MQGDWVLLLLLGLRIHLSFGVIPVEEENPVFWNQKAKEALDVAKKLQPIQTSAKNLILFLGDGMGVPTVTATRILKGQLGGHLGPETPLAMDHFPFTALSKTYNVDRQVPDSAGTATAYLCGVKANYKTIGVSAAARFNQCNSTFGNEVFSVMHRAKKAGKSVGVVTTTRVQHASPAGTYAHTVNRDWYSDADMPSSALQEGCKDIATQLISNMDIDVILGGGRKFMFPKGTPDPEYPGDSDQSGVRLDSRNLVEEWLAKYQGTRYVWNREQLMQASQDPAVTRLMGLFEPAEMKYDVNRNASADPSLAEMTEVAVRLLSRNPQGFYLFVEGGRIDQGHHAGTAYLALTEAVMFDSAIEKASQLTNEKDTLTLITADHSHVFAFGGYTLRGTSIFGLAPLNAQDGKSYTSILYGNGPGYVLNSGNRPNVTDAESGDVNYKQQAAVPLSSETHGGEDVAIFARGPQAHLVHGVQEQNYIAHVMAFAGCLEPYTDCGLAPPADENRPTTPVQNSAITMNNVLLSLQLLVSMLLLVGTALVVS

>Rat_Akp3_Rno9:85,924,232_ENSRNOT00000026294_ENSRNOG00000019375

MQGAWVLLLLGFRLQLSLSVIPVEEENPAFWNQKAADALNVAKKLQPIQTSAKNLIIFLGDGMGVPTVTATRILKGQLEGNLGPETPLAMDHFPYMALSKTYSVDRQVPDSASTATAYLCGVKTNYKTIGVSAAARFDQCNTTFGNEVLSVMYRAKKAGKSVGVVTTTRVQHASPAGTYVHTVNRNWYGDADMPASALQEGCKDIATQLVSNMDINVILGGGRKYMFPAGTPDPEYPNDVNETGTRLDGKNLVQEWLSKHQGSQYVWNRQELIQKSLDPSVTYLMGLFEPVDTKFEIQRDPLMDPSLKDMTEAALHVLSRNPKGFYLFVEGGRIDRGHHLGTAYLALTEAVMFDSAIERASQLTSEQDTLTIVTADHSHVFSFGGYTLRGTSIFGLAPLNALDGKPYTSILYGNGPGYVGTGERPNVTDAESHDPSYQQQAAVPVKSETHGGEDVAIFARGPQAHLLHGVQEQNYIAHVMAFAGCLEPYTDCGLAPPADENRPTTPVQNSTTTTTTTTTTTTTRVQNSASSLGPATGPLALALLAKALMLLLGAPADF

>Rat__Alpl_Rno5:156,501,744_ENSRNOT00000019004_ENSRNOG00000013954

MILPFLVLAIGTCLTNSFVPEKEKDPSYWRQQAQETLKNALKLQKLNTNVAKNIIMFLGDGMGVSTVTAARILKGQLHHNTGEETRLEMDKFPFVALSKTYNTNAQVPDSAGTATAYLCGVKANEGTVGVSAATERTRCNTTQGNEVTSILRWAKDAGKSVGIVTTTRVNHATPSAAYAHSADRDWYSDNEMPPEALSQGCKDIAYQLMHNIKDIDVIMGGGRKYMYPKNRTDVEYELDEKARGTRLDGLDLISIWKSFKPRHKHSHYVWNRTELLALDPSRVDYLLGLFEPGDMQYELNRNNLTDPSLSEMVEVALRILTKNPKGFFLLVEGGRIDHGHHEGKAKQALHEAVEMDEAIGKAGTMTSQKDTLTVVTADHSHVFTFGGYTPRGNSIFGLAPMVSDTDKKPFTAILYGNGPGYKVVDGERENVSMVDYAHNNYQAQSAVPLRHETHGGEDVAVFAKGPMAHLLHGVHEQNYIPHVMAYASCIGANLDHCAWASSASSPSPGALLLPLALFPLRTLF

>Chicken_Alpi_Ggal21:6,748,714_F1NN44_CHICKENSGALT00000039123

MKAFLLTLLAQLCSASLVPEREKDPEYWRQQAQETLRDALRLQHLNQNVAKNLILFLGDGMGVSTVTAARILKGQLQHRKGEESLLEMDKFPYVALAKTYNTNAQVPDSAGTATAYLCGVKANEGTVGVSAGVTRDRCNTTKGQEVTSILRWAKDEGKAVGIVTTTRVTHATPSAAYAHSANRDWYSDGEMPLDALEGGCKDIARQLVENIPDIEVILGGGRKYMFPKNTSDVEYPQEERHRGTRLDGKDLVQAWHDTKPAGKVAKYVWHRRELLALNVSRVDFLLGLFEPGDMVYELDRNNETDPSLSEMVAVAIRMLQKNPRGFFLLVEGG

>Chicken_Alpi_Ggal9:16,667,005_F1NZG4_CHICKENSGALT00000040049Chick

RWGGSSEAALGPCTFPALLAHPDAEKTPHYWNEGARRRLEAALALQPAAQRAKNIILFVGDGMGLPTVSAARIYKGQLAGGSGEESVLAMETFPHVALAKTYTIDRQVPDSAGTGTAYLCGVKANSKTVGLSGAAVYGKCRTAFGNEVDSVLHRARLAGKSVGIVTTTRVQHASPAAAYAHSASRSWYADANMPRETLRDGCKDIAHQLVHNTDINVILGGGRAYMSPRWTPDPEYPEDPAQNGTRRDGRDLVAEWLSTREGARYVWDKKGLDAVDDTSVSHLMGLFEPKDMRYELNRNATTDPSIVEMTEKAIRILRRNPNGFFLFVEGGRIDHGHHSGRAKQALMEAVMLDRAVARAGELTEPSDTLTVVTADHSHVFTFGGNTLRGASIFGLAPKKAKDKRAYTSILYGNGPGYSIRDGGRPAASLPAVEDKDYRQQAAVPLDLETHSGEDVVVLAQGPMAHLFHGVQEQHYVAHAIAYAACLKPYDARPRCGGHRRASGSTQHSPRPPLALLALCMAAFMVVG

>Turkey_Alpi1_Chr11:15,547,109_ENSMGAT00000009205_ENSMGAG00000008222Turkey

PRSLLGSMGWLLSPRICFLLLFLAPRTAIATSDPDAEKTPHYWNEGARRRLEAALALQPVAQRAKNIILFMGDGMGLPTVSAARIYKGQLAGGSGEESVLAMETFPHVALAKTYTIDRQVPDSAGTGTAYLCGVKANAKTLGLSGAAIYGKCRTAFGNEVDSVLHRARLAGKSVGIVTTTRVQHASPAAAYAHSASRSWYADANMPRETLRDGCKDIAYQLVHNTDINVILGGGRAYMSPRWTPDPEYPDDPAQNGTRRDGRDLVAEWLSTREGARYVWDKKGLDAVNDTSVSHLMGLFEPKDMKYELNRNTTTDPSIVEMTEKAIRILRRNPNGFFLFVEGGRIDHGHHSGRAKQALTEAIMLDRAVARAGELTEPSDTLTVVTADHSHVFTFGGNTLRGASIFGLAPKKAKDKRTYTSILYGNGPGYSIRDGGRPAASLPAVALSTEDKDYRQQAAVPLDLETHSGEDVMVLAQGPMAHLFHGVQEQHYIAHAIAYAACLKPYDARSRCGAPRRASGSTQHSPQLPLTLLALCMSAF

>Turkey_Alpi2_Chr11:15,550,445_ENSMGAT00000009277_ENSMGAG00000008233Turkey

MRLLVPLTLCLGLWAELGAAVIPVEEEDPTFWNRKAAAALNATLKIEPRMTEAKNLIIFLGDGFGVPSITATRILKGQLKGNLGPETPLALDSFPYVALSKTYTVDRVVPDSAGTATAYLCGVKGNYKTVGLSAAARYGQCNTTKGNEVISVLERARNAGKAVGIVTTSRVQHASPSGTYAHVVDRNWYADASMPAEAIAEGCKDIAWQLVHNVDINVILGGGRIYMTPAGTPDPEYPEYSSENGIRNDGQNLIDMWLSKQQGARYVWNRTEMLAAAADPSVTYLMGLFEPMDMKYEMVHNSTLDPSLTEMTEAAINVLSRNPNGFYLFVEGGRIDHGHHEGMAQRALTEAVEFDTAIERAGELLDEADTLTVVTADHSHVFSFGGYTLRGTSIFGLAPLQATDQKNYTSILYGNGPGYPGATRPDVNSSIAEEFSYMQQAAVPLSSETHGGEDVAILAKGPMAYLFHGQEQNYIAHAMAYAACLEPYENCRQRTNAAPSAHLTPLALLLPALLLSLF

>Mgal_Alpl_Chr23:6,879,039_ENSMGAT00000008255_ENSMGAG00000007370_NotWellAssembled

MKAFLLTLLAQLCSASLVPEREKDPEYWRQQAQETLRDALRLQHLNQNVAKNLILFLGDGMGVSTVTAARILKGQLQHRKGEESLLEMDKFPYVALAKTYNTNAQVPDSAGTATAYLCGVKANEGTVGVSAGVTRDRCNTTKGQEVTSILRWAKDEGKAVGIVTTTRVTHATPSAAYAHSANRDWYSDGEMPLDALEGGCKDIARQLVENIPDIEVILGGGRKYMFPKNTSDVEYPQEERHRGTRLDGKDLVQAWHDAKPAGKVAKYVWHRRELLALNLSRVDFLLGLFEPGDMVYELDRNNETDPSLSEMVAVAIRMLQKNPRGFFLLVEGG

>Turkey_Alpi1_P_003209207[MeleagrisgallopavoTurkey]

MGWLLSPRICFLLLFLAPRTAIATSDAEKTPHYWNEGARRRLEAALALQPVAQRAKNIILFMGDGMGLPTVSAARIYKGQLAGGSGEESVLAMETFPHVALAKTYTIDRQVPDSAGTGTAYLCGVKANAKTLGLSGAAIYGKCRTAFGNEVDSVLHRARLAGKSVGIVTTTRVQHASPAAAYAHSASRSWYADANMPRETLRDGCKDIAYQLVHNTDINVILGGGRAYMSPRWTPDPEYPDDPAQNGTRRDGRDLVAEWLSTREGARYVWDKKGLDAVNDTSVSHLMGLFEPKDMKYELNRNTTTDPSIVEMTEKAIRILRRNPNGFFLFVEGGRIDHGHHSGRAKQALTEAIMLDRAVARAGELTEPSDTLTVVTADHSHVFTFGGNTLRGASIFGLAPKKAKDKRTYTSILYGNGPGYSIRDGGRPAASLPAVEDKDYRQQAAVPLDLETHSGEDVMVLAQGPMAHLFHGVQEQHYIAHAIAYAACLKPYDARSRCGAPRRASGSTQHSPQLPLTLLALCMSAFMVVG

>Ciona savignyi_Baa92180.1|endoderm-alkalinephosphatase[Cionasavignyi]

MSMICFVTMMCVCSLGTAQVLTERSAHFWELRNQAELDEAITKQSLNIRKAKNVIIFVGDGMGITTITSGRILKGQVSGTSGEETKLAMDKFPFSGISKTYSVNKQVSDSASTATAFLTGVKTNDFILGLTGSAQRGICKGSIDENNIVTSVLIEAKNAGKSAGFVTTTRINHATPGATYAHTPERMWYGDADLTEEAKANGCKDVAQQFIDNSHLFTVALGGGRQYFRPNTTQDEEYPNKTNARLDGQDLIEQWKQIQLQQGNRAAYVWNATEFAGINPDNTDSLLGLFQPKDMHYEAHRSGDVAGEPSLSEMTAKAISLLKKNEEGYILLVEGGRIDHGHHEGNAYLALHDLVAFDDAIDTAVQMTSDDETMLIVTADHSHVFTIGGYSDRGNPIFGLAPNAIKPTLGDDNKTFTTLLYGNGPGYAFESCERENVTGVPTDVSTYLQQSAVPLSYETHGGEDVIIMSRGPMAHLFEGVHEQTYIAHVIRYATCIGKLSKDCNERFNPPKENGVILYFLGISMTSSKAVLALYVTLALLIVTSIVAIAANIHIYRMVSSKPKSEQMQKV

>Ciona intestinalis_Alp_NP_001027596endodermalkalinephosphataseCionaintestinalis]

MLDMMFHQGLLLLCASVAVIGQDLTEKSAAYWEQVSEAELLETLQYQKLNIKKAKNVIIFIGDGMGVTTVTAGRILKGQNSGASGEETKLAMDKLPYTGVSRTYSVNRQVSDSASTATAFLTGVKTNDYVLGLNGNSVKGICAGSINESNLLTSVLLEAKMAGMSAGIVTTTTINHATPAAAYANSPDRLWYSDAEMTAEAKENGCKDIAQQFIDKSDQFTVVLGGGRQYFKPNTTFDVEYTDRANLRLDGQDLIEVWKAKQSDRNSAYVWNKEQFDQVDVAKTDSLLGLFEPSHMNYEAHRAQDGAGEPSLKDMTSKAIRMLKKNDQGFILLVEGGKIDHGHHAGKAYLALHDLVALDDAIEAAVEMTSDDETMIIVTADHSHVFTIGGYSHRGNPIFGAAPNVNNPKLVDDGKPFTTLLYGNGPGHSTLNGVGSCERENITLIATDDPNYKQQSAVPLPSETHGGEDVVIMARGPMAHLFEGVHEQSYIAHIIRYATCIGKKSKNCAAQLEQSTDLIFVSFLGFRLSSGQAQLALYITFGLLMAACIIAIAANLQLCRMARQSARKHEDPKVLNEKV

>Opossum_Alpi_Mdom2:537,202,874_ENSMODT00000003218

MQGIFALFLVGLYFHTAKGEIPEEEENPSFWNRQAAEAIKTAQQLQPIQTAAKNLILFLGDGMGVPTVTATRILKGKLSGNLGPETPLAMDRFPYVALSKTYNVDRQVPDSAGTATAYLCGVKGNYKTIGLSAAARVDQCNSTFGNEVYSVLQRAKKAGKSVGVVTTTRVQHASPSGTYAHVVNRDWYSDANMPADALRSGCKDIASQLISNVDIDVILGGGRKYMFPVGTPDPEYPNLSSQNGIRRDDRNLVQEWLDARQGAKYVWDRQELLNAAENLEVTHIMGLFEPGDTKYDLQRNTSVDPSLMEMTEVAIKILRRNPKGYYLFVEGGRIDHGHHDGNAHLALTEAVMFDSTIDKAVQMTNEKDTLIAVTADHSHVFSFGGYTYRGSSIFGLAPSKAQDNKAYTSILYGNGPGYVNGTRPDVKESVSEDVSYKQQAAVPLSSETHGGEDVAIFARGPQAHLFHGVQEETYVAHVMAFAGCLEPYENCGLSWPNSSLQTSVS

>Opossum_Alpl_Mdom4:356,739,559_ENSMODT00000020333

GSTMFLLLLCLMAGTCLSIVPEKEKNPWYWREQAQRTLQHSLKLQNLNTNVARNVILFLGDGMGVSTVTAARILKGQLHHKLGEDFQLEMDKFPFVALSKTYNTNAQVPDSAGTATAYLCGVKSNEGTVGVSAAVTRSQCNTTKGNEVTSILRWAKDAGKSVGIVTTTRVNHATPSAAYAHSADRDWYSDNEMPPEALNQGCKDIAYQLMHNIPDIEVIMGGGRKYMFPKNTTDVEYGDDPKARGTRLDGQVLIDVWKGKKPRNKNAHYVWNRMQLMNLDLNNVDYLLGLFEPSDMVYELNRNNKTDPSLSEMVEVAIKILRKNPKGFFLLVEGGRIDHGHHEGKAKQALHEAVEMDRAIGLAGTLTSLEDTLTVVTADHSHVFTFGGYTPRGNSIFGLAPVVSDTDNKPYTSILYGNGPGYKVVAGQRENVSTVDYAHDNYQAQSAVPLSYETHGGEDVAVFAKGPMAHLLHGVQEQNYIPHVMAYASCIGSIMDHCQSHPSAGLAPQPALFSGLLTLLLLLSLLF
