# Supplementary material for: Dynamic Evolution of the LPS-Detoxifying Enzyme Intestinal Alkaline Phosphatase in Zebrafish and Other Vertebrates
Source: Front Immunol. 2012 Oct 12;3:314. doi: 10.3389/fimmu.2012.00314 (PMC3469785; doi:10.3389/fimmu.2012.00314)
Supplement: Supplementary Data sheet S4 — Alp sequences used to generate Figure 6. [file 35467_Guillemin_DataSheet4.DOCX]

>Human_ALPL_Hsa1:21,835,858_NP_000469.3_[Homosapiens]ENSG00000162551

MISPFLVLAIGTCLTNSLVPEKEKDPKYWRDQAQETLKYALELQKLNTNVAKNVIMFLGDGMGVSTVTAARILKGQLHHNPGEETRLEMDKFPFVALSKTYNTNAQVPDSAGTATAYLCGVKANEGTVGVSAATERSRCNTTQGNEVTSILRWAKDAGKSVGIVTTTRVNHATPSAAYAHSADRDWYSDNEMPPEALSQGCKDIAYQLMHNIRDIDVIMGGGRKYMYPKNKTDVEYESDEKARGTRLDGLDLVDTWKSFKPRYKHSHFIWNRTELLTLDPHNVDYLLGLFEPGDMQYELNRNNVTDPSLSEMVVVAIQILRKNPKGFFLLVEGGRIDHGHHEGKAKQALHEAVEMDRAIGQAGSLTSSEDTLTVVTADHSHVFTFGGYTPRGNSIFGLAPMLSDTDKKPFTAILYGNGPGYKVVGGERENVSMVDYAHNNYQAQSAVPLRHETHGGEDVAVFSKGPMAHLLHGVHEQNYVPHVMAYAACIGANLGHCAPASSAGSLAAGPLLLALALYPLSVLF

>Human_ALPP_Hsa2:233,243,244_NP_001623.3_[Homosapiens]_ENST00000392027

MLGPCMLLLLLLLGLRLQLSLGIIPVEEENPDFWNREAAEALGAAKKLQPAQTAAKNLIIFLGDGMGVSTVTAARILKGQKKDKLGPEIPLAMDRFPYVALSKTYNVDKHVPDSGATATAYLCGVKGNFQTIGLSAAARFNQCNTTRGNEVISVMNRAKKAGKSVGVVTTTRVQHASPAGTYAHTVNRNWYSDADVPASARQEGCQDIATQLISNMDIDVILGGGRKYMFRMGTPDPEYPDDYSQGGTRLDGKNLVQEWLAKRQGARYVWNRTELMQASLDPSVTHLMGLFEPGDMKYEIHRDSTLDPSLMEMTEAALRLLSRNPRGFFLFVEGGRIDHGHHESRAYRALTETIMFDDAIERAGQLTSEEDTLSLVTADHSHVFSFGGYPLRGSSIFGLAPGKARDRKAYTVLLYGNGPGYVLKDGARPDVTESESGSPEYRQQSAVPLDEETHAGEDVAVFARGPQAHLVHGVQEQTFIAHVMAFAACLEPYTACDLAPPAGTTDAAHPGRSVVPALLPLLAGTLLLLETATAP

>Human_ALPPL2_Hsa2:233,271,553_NP_112603.2_[Homosapiens]_ENST00000295453

MQGPWVLLLLGLRLQLSLGIIPVEEENPDFWNRQAAEALGAAKKLQPAQTAAKNLIIFLGDGMGVSTVTAARILKGQKKDKLGPETFLAMDRFPYVALSKTYSVDKHVPDSGATATAYLCGVKGNFQTIGLSAAARFNQCNTTRGNEVISVMNRAKKAGKSVGVVTTTRVQHASPAGAYAHTVNRNWYSDADVPASARQEGCQDIATQLISNMDIDVILGGGRKYMFPMGTPDPEYPDDYSQGGTRLDGKNLVQEWLAKHQGARYVWNRTELLQASLDPSVTHLMGLFEPGDMKYEIHRDSTLDPSLMEMTEAALLLLSRNPRGFFLFVEGGRIDHGHHESRAYRALTETIMFDDAIERAGQLTSEEDTLSLVTADHSHVFSFGGYPLRGSSIFGLAPGKARDRKAYTVLLYGNGPGYVLKDGARPDVTESESGSPEYRQQSAVPLDGETHAGEDVAVFARGPQAHLVHGVQEQTFIAHVMAFAACLEPYTACDLAPRAGTTDAAHPGPSVVPALLPLLAGTLLLLGTATAP

>Human_ALPI_Hsa2:233,320,833_NP_001622.2_[Homosapiens]_ENSG00000163295

MQGPWVLLLLGLRLQLSLGVIPAEEENPAFWNRQAAEALDAAKKLQPIQKVAKNLILFLGDGLGVPTVTATRILKGQKNGKLGPETPLAMDRFPYLALSKTYNVDRQVPDSAATATAYLCGVKANFQTIGLSAAARFNQCNTTRGNEVISVMNRAKQAGKSVGVVTTTRVQHASPAGTYAHTVNRNWYSDADMPASARQEGCQDIATQLISNMDIDVILGGGRKYMFPMGTPDPEYPADASQNGIRLDGKNLVQEWLAKHQGAWYVWNRTELMQASLDQSVTHLMGLFEPGDTKYEIHRDPTLDPSLMEMTEAALRLLSRNPRGFYLFVEGGRIDHGHHEGVAYQALTEAVMFDDAIERAGQLTSEEDTLTLVTADHSHVFSFGGYTLRGSSIFGLAPSKAQDSKAYTSILYGNGPGYVFNSGVRPDVNESESGSPDYQQQAAVPLSSETHGGEDVAVFARGPQAHLVHGVQEQSFVAHVMAFAACLEPYTACDLAPPACTTDAAHPVAASLPLLAGTLLLLGASAAP

>Mouse_Alppl2_Mmu1:88,983,265_ENSMUST00000027455[Mouse

MWGACLLLLGLSLQVCPSVIPVEEENPAFWNRKAAEALDAAKKLKPIQTSAKNLVILMGDGMGVSTVTATRILKGQQQGHLGPETQLAMDRFPHMALSKTYNTDKQIPDSAGTGTAFLCGVKTNMKVIGLSAAARFNQCNTTWGNEVVSVMHRAKKAGKSVGVVTTTSVQHASPAGTYAHTVNRGWYSDAQMPASALQDGCKDISTQLISNMDIDVILGGGRKFMFPKGTPDQEYPTDTKQAGTRLDGRNLVQEWLAKHQGARYVWNRSELIQASLNRSVTHLMGLFEPNDMKYEIHRDPAQDPSLAEMTEVAVRMLSRNPKGFYLFVEGGRIDHGHHETVAYRALTEAVMFDSAVDKADKLTSEQDTMILVTADHSHVFSFGGYTQRGASIFGLAPFKAEDGKSFTSILYGNGPGYKLHNGARADVTEEESSNPTYQQQAAVPLSSETHSGEDVAIFARGPQAHLVHGVQEQNYIAHVMAFAACLEPYTDCGLASPAGQSSAVSPGYMSTLLCLLAGKMLMLMAAAEP

>Mouse_Alpi_Mmu1:88,994,579_ENSMUST00000113270[mouse

MQGDWVLLLFLGLRIHLSFGIIPAEEENPAFWNKKAAEALDAAKKLQPIQTSAKNLIIFLGDGMGVPTVTATRILKGQLEGHLGPETPLAMDLFPYMALSKTYNVDRQVPDSAGTATAYLCGVKANYKTIGLSAAARLDQCNTTFGNEVFSVMYRAKKAGKSVGVVTTTRVQHASPAGTYAHTVNRNWYSDAEMPASALQDGCKDIATQLISNMDIDVILGGGRKFMFPKGTPDPEYPSDSNQSGTRLDDQNLVQTWLSKHQGARYVWNRSELIQASQDPAVTHLMGLFEPTEMKYDANRNPSVDPSLAEMTEVAVRMLSRNPQGFYLFVEGGRIDQGHHAGTAYLALTEAVMFDSAIEKASQLTNEKDTLILITADHSHVFAFGGYTLRGTSIFGLAPLKALDDKSYTSILYGNGPGYELKSGNRPNVTEAQSVDPNYKQQAAVPLSSETHGGEDVAIFARGPQAHLVHGVQEQNYIAHVMAFAGCLEPYTDCGLAPPAGQSPVITPGQATTTNNAAGQATTTNNAAGQATVLLSLQLLVSMLLLVGTAMVVS

>Mouse_Akp3_Mmu1:89,021,583_ENSMUST00000044878[mouse

MQGTWVLLLLGLRLQLSLSVIPVEEENPAFWNKKAAEALDAAKKLQPIQTSAKNLIIFLGDGMGVPTVTATRILKGQLEGHLGPETPLAMDRFPYMALSKTYSVDRQVPDSASTATAYLCGVKTNYKTIGVSAAARFDQCNTTFGNEVFSVMYRAKKAGKSVGVVTTTRVQHASPSGTYVHTVNRNWYGDADMPASALREGCKDIATQLISNMDINVILGGGRKYMFPAGTPDPEYPNDANETGTRLDGRNLVQEWLSKHQGSQYVWNREQLIQKAQDPSVTYLMGLFEPVDTKFDIQRDPLMDPSLKDMTEAAVKVLSRNPKGFYLFVEGGRIDRGHHLGTAYLALTEAVMFDLAIERASQLTSERDTLTIVTADHSHVFSFGGYTLRGTSIFGLAPLNALDGKPYTSILYGNGPGYVGTGERPNVTAAESSGSSYRQQAAVPVKSETHGGEDVAIFARGPQAHLLHGVQEQNYIAHVMAFAGCLEPYTDCGLAPPADESQTTTTTRQTTITTTTTTTTTTTTPVHNSARSLGPATAPLALALLAGMLMLLLGAPAES

>Mouse_Alpl_Mmu4:137,297,648_ENSMUST00000030551

MISPFLVLAIGTCLTNSFVPEKERDPSYWRQQAQETLKNALKLQKLNTNVAKNVIMFLGDGMGVSTVTAARILKGQLHHNTGEETRLEMDKFPFVALSKTYNTNAQVPDSAGTATAYLCGVKANEGTVGVSAATERTRCNTTQGNEVTSILRWAKDAGKSVGIVTTTRVNHATPSAAYAHSADRDWYSDNEMPPEALSQGCKDIAYQLMHNIKDIDVIMGGGRKYMYPKNRTDVEYELDEKARGTRLDGLDLISIWKSFKPRHKHSHYVWNRTELLALDPSRVDYLLGLFEPGDMQYELNRNNLTDPSLSEMVEVALRILTKNLKGFFLLVEGGRIDHGHHEGKAKQALHEAVEMDQAIGKAGAMTSQKDTLTVVTADHSHVFTFGGYTPRGNSIFGLAPMVSDTDKKPFTAILYGNGPGYKVVDGERENVSMVDYAHNNYQAQSAVPLRHETHGGEDVAVFAKGPMAHLLHGVHEQNYIPHVMAYASCIGANLDHCAWAGSGSAPSPGALLLPLAVLSLRTLF

>Chimpanzee_Alpi2_Ptr2B:2B:237,629,972_XP_003309566_[Pantroglodytes]

MQGPWVLLLLGLRLQLSLGVIPAEEENPAFWNRQAAEALDAAKKLQPIQKVAKNLILFLGDGLGVPTVTATRILKGQKNGKLGPETPLAMDRFPYLALSKTYNVDRQVPDSAATATAYLCGVKANFQTIGLSAAARFNQCNTTRGNEVISVMNRAKQAGKSVGVVTTTRVQHASPAGTYAHTVNRNWYSDADMPASARQDGCQDIATQLISNMDIDVILGGGRKYMFPMGTPDPEYPGDASQNGIRLDGKNLVQEWLAKHQGAWYVWNRTELVQASLDPSVTHLMGLFEPGDTKYEIHRDPTLDPSLMEMTEAALRLLSRNPRGFFLFVEGGRIDHGHHEGVAYQALTEAVMFDDAIERAGQLTSEEDTLTLVTADHSHVFSFGGYTLRGSSIFGLAPSKAQDSKAYTSILYGNGPGYVFNSGVRPDVNESESGSPDYQQQAAVPLSSETHGGEDVAVFARGPQAHLVHGVQEQSFVAHVMAFAACLEPYTACDLAPPACPTDAAHPVAASLPLLAGTLLLLGASAAP

>Chimpanzee_ALPI_Ptr2B:237,674,869_ENSPTRT00000024165

EEENPAFWNRQAAEALDAAKKLQPIQKVAKNLILFLGDGLGVPTVTATRILKGQKNGKLGPETPLAMDRFPYLALSKTYNVDRQVPDSAATATAYLCGVKANFQTIGLSAAARFNQCNTTRGNEVISVMNRAKQAGKSVGVVTTTRVQHASPAGTYAHTVNRNWYSDADMPPQPQDGCQDIATQLISNMDIDVILGGGRKYMFPMGTPDPEYPGDPARGIRLDGKNLVRNGGKLFEPGDTKYEIHRDPTLDPSLMEMTEAALRLLSRNPRGFFLFVEGGRIDHGHHEGVAYQALTEAVMFDDAIERAGQLTSEEDTLTLVTADHSHVFSFGGYTLRGSSIFGLAPQGSDSKAYTSILYGNGPGYVLACGEEDYQQQAAVPLSSETHGGEDVAVFARGPQAHLVHGVQEQSFVAHVMAFAACGALHGCDLAPPACPTGRVAATAGPLLLLVRCSLSAPLR

>Chimpanzee_Alpi2_Ptr2B:237,628,025_XP_003309565_[Pantroglodytes]

MQGPWVLLLLGLRLQLSLGIIPVEEENPDFWNRQAAEALGAAKKLQPAQTAAKNLIIFLGDGMGVSTVTAARILKGQKKDKLGPETFLAMDRFPYVALSKTYSVDKHVPDSGATATAYLCGVKGNFQTIGLSAAARFNQCNTTRGNEVISVMNRAKKAGKSVGVVTTTRVQHASPAGAYAHTVNRNWYSDADVPASARQEGCQDIATQLISNMDIDVILGGGRKYMFPMGTPDPEYPDDYSQGGTRLDGKNLVQEWLAKHQGARYVWNRTELMQASLDPSVTHLMGLFEPGDMKYEIHRDSTLDPSLMEMTEAALRLLSRNPRGFFLFVEGGRIDHGHHESRAYRALTETIMFDDAIERAGQLTSEEDTLSLVTADHSHVFSFGGYPLRGSSIFGLAPGKARDRKAYTVLLYGNGPGYVLKDGARPDVTESESGSPEYRQQSAVPLDGETHAGEDVAVFARGPQAHLVHGVQEQTFIAHVMAFAACLEPYTACDLAPPAGTTDAAHPGPSVVPALLPLLAGTLLLLGTATAP

>Chimpanzee_ALPPL2_Ptr2B:237,601,898_ENSPTRT00000024162

MLGPCMLLLLLLGLRLQLSLGIIPVEEENPDFWNRQAAEALGAAKKLQPAQTAAKNLIIFLGDGMGVSTVTAARILKGQKKDRLGPEIPLAMDRFPYVALSKTYNVDKHVPDSGATATAYLCGVKGNFQTIGLSAAARFNQCNTTRGNEVISVMNRAKKAGKSVGVVTTTRVQHASPAGTYAHTVNRNWYSDANMPCSARREGCKDIATQLISNMDIDVILGGGRKYMFRMGTPDPEYPDDYSQGGTRLDGKNLVQEWLAKRQGARYVWNRTELMQASLDPSVTHLMGLFEPGDMKYEIHRDSTLDPSLMEMTEAALRLLSRNPRGFFLFVEGGRIDHGHHESRAYRALTETIMFDDAIERAGQLTSEEDTLSLVTADHSHVFSFGGYPLRGSSIFGLAPGKAQDRKAYTVLLYGNGPGYVLKDGARPDVTESESGSPEYRQQSAVPLDEETHAGEDVAVFARGPQAHLVHGVQEQTFIAHVMAFAACLEPYTACDLAPPAGTTDAAHPGPSVVPALLPLLAGTLLLLGTATAP

>Chimpanzee_Alpp1_Ptr2B:237,629,972_XP_001146156.2_[Pan_troglodytes]

MLGPCMLLLLLLGLRLQLSLGIIPVEEENPDFWNRQAAEALGAAKKLQPAQTAAKNLIIFLGDGMGVSTVTAARILKGQKKDRLGPEIPLAMDRFPYVALSKTYNVDKHVPDSGATATAYLCGVKGNFQTIGLSAAARFNQCNTTRGNEVISVMNRAKKAGKSVGVVTTTRVQHASPAGTYAHTVNRNWYSDANMPCSARREGCKDIATQLISNMDIDVILGGGRKYMFRMGTPDPEYPDDYSQGGTRLDGKNLVQEWLAKRQGARYVWNRTELMQASLDPSVTHLMGLFEPGDMKYEIHRDSTLDPSLMEMTEAALRLLSRNPRGFFLFVEGGRIDHGHHESRAYRALTETIMFDDAIERAGQLTSEEDTLSLVTADHSHVFSFGGYPLRGSSIFGLAPGKAQDRKAYTVLLYGNGPGYVLKDGARPDVTESESGSPEYRQQSAVPLDEETHAGEDVAVFARGPQAHLVHGVQEQTFIAHVMAFAACLEPYTACDLAPPAGTTDAAHPGPSVVPALLPLLAGTLLLLGTATAP

>Chimpanzee_ALPL_Ptr1:21,545,230_[Pantroglodytes]_ENSPTRT00000000592

MISPFLVLAIGTCLTNSLVPEKEKDPKYWRDQAQETLKYALKLQKLNTNVAKNVIMFLGDGMGVSTVTAARILKGQLHHNPGEETRLEMDKFPFVALSKTYNTNAQVPDSAGTATAYLCGVKANEGTVGVSAATERSRCNTTQGNEVTSILHWAKDAGKSVGIVTTTRVNHATPSAAYAHSADRDWYSDNEMPPEALSQGCKDIAYQLMHNIRDIDVIMGGGRKYMYPKNKTDVEYESDEKARGTRLDGLDLVDTWKSFKPRHKHSHFIWNRTELLTLDPHNVDYLLGLFEPGDMQYELNRNNVTDPSLSEMVVVAIQILRKNPKGFFLLVEGGRIDHGHHEGKAKQALHEAVEMDRAIGQAGSLTSSEDTLTVVTADHSHVFTFGGYTPRGNSIFGLAPMLSDTDKKPFTAILYGNGPGYKVVGGERENVSMVDY

>Gorilla_Alpi_Ggo2b:121,629,034_ENSGGOT00000006932[Gorilla]

MLGPCMLLLLLLGLRLQLSLGIIPVEEENPDFWNRQAAEALGAAKKLQPAQTAAKNLIIFLGDTYNVDKVPDSAATATAYLCGVKGNFQTIGLSAPARFNQCNTTRGNEVISVMNRAKKAGKSVGVVTTTWVQHASPAGTYAHTVNRNWYSDTNMPCSARQEGCKDIATQLISNMDIDVILGGGRKYMFPMGTPDPEYPDDYSQGGTRLDGKNLVQEWLAKHQGARYVWNHTELMQASLDPSVTHLMGLFEPGDMKYEIHRDSTLDPSLMEMTEAALRLLSRNPRGFFLFVEGGRIDHGHHESRAYRALTETIMFDDAIERAGQLTSEEDTLSLVTADHSHVFSFGGYPLRGSSIFGLAPGKARDRKAYTVLLYGNGPGYVLKDGARPDVTESESGSPEYRQQSAVPLDGETHAGEDVAVFARGPQAHLVHGVQEQTFIAHVMAFAACLEPYTACDLAPPAGTTDAAHPGPSVVPGLLPLLAGTLLLLGTATAP

>Gorilla_Alpi2_Ggo2b:121,675,563_ENSGGOT00000029718[Gorilla]

MQGPWVLLLLGLRLQLSLGVIPAEEENPAFWNRQAAEALDAAKKLQPIQKVAKNLILFLGDGLGVPTVTATRILKGQKNGKLGPETPLAMDRFPYLALSKTYNVDRQVPDSAATATAYLCGVKANFQTIGLSAAARFNQCNTTRGNEVISVMNRAKQAGKSVGVVTTTRVQHASPAGTYAHTVNRNWYSDADMPASARQDGCQDIATQLISNMDIDVILGGGRKYMFPMGTPDPEYPSDASQNGIRLDGKNLVQEWLAKHQGAWYVWNRTELMQASLDQSVTHLMGLFEPGDTKYEINRDPTLDPSLMEMTEAALRLLSRNPRGFYLFVEGGRIDHGHHEGVAYQALTEAVMFDDTIERAGQLTSEEDTLTLVTADHSHVFSFGGYTLRGSSIFGLAPSKAQDSKAYTSILYGNGPGYVFNSGVRPDVNDSESGSPDYQQQAAVPLSSETHGGEDVAVFARGPQAHLVHGVQEQSFVAHVMAFAACLEPYTACDLALPACTTDAAHPVAASLPLLAGTLLLLGASAAP

>Gorilla_Ggo1:22,219,201_Alpl_ENSGGOT00000010755[Gorilla]

MISPFLVLAIGTCLSNSLVPEKEKDPKYWRDQAQETLKYALKLQRLNTNVAKNVIMFLGDGMGVSTVTAARILKGQLHHNPGEETRLEMDKFPFVALSKTYNTNAQVPDSAGTATAYLCGVKANEGTVGVSAATERSRCNTTQGNEVTSILRWAKDAGKSVGIVTTTRVNHATPSAAYAHSADRDWYSDNEMPPEALSQGCKDIAYQLMHNIRDIDVIMGGGRKYMYPKNKTDVEYESDEKARGTRLDGLDLVDTWKSFKPRHKHSHFIWNRTELLTLDPHNVDYLLGLFEPGDMQYELNRNNMTDPSLSEMVVVAIQILRKNPKGFFLLVEGGRIDHGHHEGKAKQALHEAVEMDRAIGQAGSLTSSEDTLTVVTADHSHVFTFGGYTPRGNSIFGLAPMLSDTDKKPFTAILYGNGPGYKVVGGERENVSMVDYAHNNYQAQSAVPLRHETHGGEDVAVFSKGPMAHLLHGVHEQNYIPHVMAYAACIGANLDHCAPASSAGSLAAGPLLLALALFPLSVLF

>Orangutan_Alpl_Pabe1:208,882,705_ENSPPYT00000002103[Pongo_abelii_oran]

MISPFLVLAIGTCLTNSLVPEKEKDPKYWRDQAQETLKYALELQKLNTNVAKNVIMFLGDGMGVSTVTAARILKGQLHHNPGEETRLEMDKFPFVALSKTYNTNAQVPDSAGTATAYLCGVKANEGTVGVSAATERSRCNTTQGNEVTSILRWAKDAGKSVGIVTTTRVNHATPSAAYAHSADRDWYSDNEMPPEALSQGCKDIAYQLMHNIRDIDVIMGGGRKYMYPKNKTDVEYEIDEKARGTRLDGLDLVDTWKSFKPRHKHSHFIWNRTELLTLDPHNVDYLLGLFEPGDMQYELNRNNVTDPSLSEMVVVAIQILRKNPKGFFLLVEGGRIDHGHHEGKAKQALHEAVEMDRAIGQAGSLTSSEDTLTVVTADHSHVFTFGGYTPRGNSIFGLAPMLSDTDKKPFTAILYGNGPGYKVVGGERENVSMVDYAHNNYQAQSAVPLRHETHGGEDVAVFSKGPMAHLLHGVHEQNYIPHVMAYAACIGANLDHCASASSAGSLAAGPLLLALALFPLSVLF

>Orangutan_Alpi_Pabe2b:124,766,615_ENSPPYT00000015437[Pongo_abelii_oran]

IPRIPASPLSCCPPDMRGPCVLLLLLLLCLKLQLSLCIIPVEEENPDFWNRQAAEALGAAKKLQPVQTAAKNLIIFLGDGMGVSTVTAARILKGHKKDKLGPEIPLAMDRFPYVALSKTYSVDKHVPDSAATATAYLCGVKGNFQTIGLSAAARFNQCNTTRGNEVISVMNRAKKAGKSVGVVTTTRVQHASPAGAYAHTVNRSWYSDANMPCSARREGCKDIATQLISNMDIDVILGGGRKYMFPMGTPDPEYPDDYSQGGTRLDGKNLVQEWLAKHQGARYVWNRTELMQASLDPSVTHLMGLFEPGDMKYEIHRDSTLDPSLMEMTEAALRLLSRNPRGFFLFVEGGRIDHGHHESRAYRALTETIMFDDAIERAGQLTSEEDTLSLVTADHSHVFSFGGYPLRGSSIFGLAPGKARDRKAYTVLLYGNGPGYVLKDGARPDVTESESESPEYRQQSAVPLDEETHAGEDVAVFARGPQAHLVHGVQEQTFVAHVMAFAACLEPYTACDLAPTAGTTDAAHPGSSSVPASLPLLAGTLLLPETATAL

>Orangutan_Alpi2_Pabe2b:124,799,448_ENSPPYT00000015439[Pongo_abelii_oran]

MQGPWVLLLLGLRLQLSLGVIPVEEENPDFWNRQAAEALGAAKKLQPAQTAAKNLILFLGDGMGVSTVTAARILKGQKKGKLGPETFLAMDRFPYVALSKTYSVDKHVPDSAATATAYLCGVKGNFETIGLSAAARFNQCNTTRGNEVISVMNRAKKAGKSVGVVTTTRVQHASPAGAYAHTVNRNWYSDADVPASARQEGCQDIATQLISNMDIDVILGGGRKYMFPMGTPDPEYPDDSRQKGTRLDGKNLVQEWLAKHQGARYVWNRTELMQASLDPSVTHLMGLFEPGDMKYEIHRDSTLDPSLMEMTEAALHLLSRNPRGFFLFVEGGRIDHGHHESRAYRALTETIMFDDAIERAGQLTSEEDTLSLVTADHSHVFSFGGYPLRGSSIFGLAPGKARDRKAYTVLLYGNGPGYVLKDGARPDVTESESESPEYRQQSAVPLDGETHAGEDVAVFARGPQAHLVHGVQEQTFVAHVMAFAACLEPYTACDLAPHAGITDAAHPGPSVVPALLSLLAGTLLLLGTATAP

>Orangutan_Alpi3_Pabe2b:124,858,716_unannotated[Pongo_abelii_oran]

MQGPWVllllglrlqfslGVIPPVEEENPAFWNRQAAEALDAAKKLQPIQKVAKNLILFLGDGMGVPTVTATRILKGQKNGKLGPETPLAMDRFPYLALSKTYNVDRQVPDSAGTATAYLCGVKANFQTIGLSAAARSNQCNTTRGNEVISVMNRAKQSGKSVGVVTTTRVQHASPAGTYAHTVNRNWYSDADMPASARQEGCQDIATQLISNMDIDVILGGGRKYMFPMGTPDPEYPSDASQNGIRLDGKNLVQEWLAKHQGARYVWNRTELMQASLDQSVTHLMGLFEPGDMKYEIHRDPTLDPSLMEMTEAALRLLSRNPRGFYLFVEGGRIDHGHHEGVAYYALTEAVMFDDAIERAGQLTSEEDTLTLVTADHSHVFSFGGYTLRGSSIFGLAPSKAQDSKDYTSILYGNGPGYVLNSGVRPDVNESESGSPDYQQQAAVPLSSETHGGEDVAVFARGPQAHLVHGVQEQSFVAHVMAFAACLEPYTACDL

>Bushbaby_GL873532.1:1,226,778_ENSOGAT00000009144_[Otolemur_garnettii]

MLASWVLLLLGLRLQLSLGVIPAEEENPAFWNLKAEEALAAARKLRPIQTAAKNLILFLGDGMGVPTVTATRILKGQMNGNLGPETPLAMDHFPYLALSKTYNVDRQVPDSAGTATAYLCGVKANYQTIGVSAAARYNECKTKHGNEVMSVMYRAKKAGKSVGVVTTTRVQHASPAGTYAHTVNRNWYSDANMPTSALQEGCQDIATQLISNMDIDVILGGGRKYMFPKGTPDPEYPSDASQDGVRLDEKNLVLEWLLKHQGAQYVWNSSDLIQASRDPSVTHLMGLFEPGDTKYETQRDPTVDPSLMEMTEAALRLLSRNPLGFYLFVEGGRIDHGHHDGKAYRALTEAVMFDSAIEKAVQLTSEQDTLILVTADHSHVFSFGGYTLRGSSIFGVAPSKAQDSKAYTSILYGNGPGFTVNASGRPDVNESESGNPNYRQQAAVPLSSETHGGEDVAVFARGPQAHLVHGVQEQSFVAHVMAFAACLEPYTACDLAPPGGHTNAAHPGPAAGPLSLPLLAGTLLLLGAAFAP

>Bushbaby_Alpl_GL873528.1:3,789,502_ENSOGAT00000011257_[Otolemur_garnettii]

MISAFLILAIGTCLTNSFVPEKEKDPEYWRRQAQQTLKHALGLQKLNTNVAKNVIMFLGDGMGVSTVTAARILKGQLHHNPGEETMLEMDKFPFVALSKTYNTNAQVPDSAGTATAYLCGVKANEGTVGVSAATERTQCNTTQGNEVTSILRWAKDAGKSVGIVTTTRVNHATPSAAYAHSADRDWYSDNEMPPEALSQGCKDIAYQLMHNIRNIDVIMGGGRKYMYPKNRTDVEYEMDEKARGTRLDGLNLIDVWKSFKPKRKHSHFVWNRTELMNLNPSTVDYLLGLFEPGDLQYELNRNNLTDPSLSEMVKVAIQVLKKNPKGFFLLVEGGRIDHGHHEGKAKQALHEAVEMDRAIGLAGDMTSLEDTLTVVTADHSHVFTFGGYTPRGNSIFGLAPMVSDTDKKPFTAILYGNGPGYKVVDGERENVSTVDYAHNNYQAQSAVPLRYETHGGEDVAVFAKGPMAHLLHGVHEQNYIPHVMAYASCVGANLDHCTRASSAGSPALVPLLLLLALLPLSILF

>Gibbon_Alppl2_GL397366.1:5,730,772_ENSNLET00000018155[Nomascus_leucogenys_gibbon]

MWGPCVLRLLGLRLQLSLGIIPVEEENPDFWNRQAAEALGAAKKLQPAQTAAKNLILFLGDGMGVSTVTAARILKGQKKGKLGPETFLAMDRFPYVALSKTYSVDKHVPDSAATATAYLCGVKSNFQTIGLSAAARFNQCNTTRGNEVISVMNRAKKAGKSVGVVTTTRVQHASPAGAYAHTVNRNWYSDADMPASARREGCRDIATQLISNMDIDVILGGGRKYMFPMGTPDPEYPDDYSQGGTRLDGKNLVQEWLAKHQGARYVWNRTELMQASLDPSVTHLMGLFEPGDMKYEIHRDSTLDPSLVEMTEAALHLLSRNPCGFFLFVEGGRIDHGHHESRAYRALTETVMFDDAIERAGQLTSEEDTLTLVTADHSHVFSFGGYPLRGSSIFGLAPGKARDRKSYTVLLYGNGPGYVLKDGARPDVTESESGSPEYRQQSAVPLDGETHAGEDVAVFARGPQAHLVHGVQEQSFVAHVMAFAACLEPYKACDLEAPTCSTNAAHPGTSVIPALLPLLAGTLRTATAP

>Gibbon_Alppl2b_GL397366:5,749,332_ENSNLET00000018171[Nomascus_leucogenys]

MQGPWVLLLLGLRLQLSLGVIPVEEENPAFWNRQAAEALDAAKKLQPIQKVAKNLILFLGDGMGVPTVTATRILKGQKNGKLGPETPLAMDRFPYLALSKTYNVDRQVPDSAGTATAYLCGVKGNFQTIGLSAAARFNQCNTTRGNEVISVMNRAKQAGKSVGVVTTTRVQHASPAGTYAHTVNRNWYSDADMPASARQDGCQDIATQLISNMDIDVILGGGRKYMFPMGTPDPEYPSDASQNGIRLDGKNLVQEWLAKHQGARYVWNRTELTQASLDQSVTHLMGLFEPGDTKYEINRDPTLDPSLMEMTEAALRLLSRTPGFYLFVEGGRIDHGHHEGVAYRALTEAVMFDDAIERAGQLTSEEDTLTLVTADHSHVFSFGGYTLRGSSIFGLAPSKAQDSKVYTSILYGNGPGYVLNSGVRPDVNESESGSPDYQQQAAVPLSSETHGGEDVAVFARGPQAHLVHGVQEQSFVAHVMAFAACLEPYTACDLAPPGTTDAAHPVPASLPLLAGTLLLLGASAAP

>Gibbon_Alpl_GL397339:2,508,317_ENSNLET00000010290[Nomascus_leucogenys]

MISPFLVLAIGTCLTSSLVPEKEKDPKYWRDQAQETLKYALELQKLNTNVAKNVIMFLGDGMGVSTVTAARILKGQLHHNPGEETRLEMDKFPFVALSKTYNTNAQVPDSAGTATAYLCGVKANEGTVGVSAATERSRCNTTQGNEVTSILRWAKDAGKSVGIVTTTRVNHATPSAAYAHSADRDWYSDNEMPPEALSQGCKDIAYQLMHNIRDIDVIMGGGRKYMYPKNKTDVEYEIDEKARGTRLDGLDLVDTWKSFKPRHKHSHFIWNRTELLTLDPHNVDYLLGLFEPGDMQYELNRNNVTDPSLSEMVVVAIQILRKNPKGFFLLVEGGRIDHGHHEGKAKQALHEAVEMDRAIGQAGSMTSSEDTLTVVTADHSHVFTFGGYTPRGNSIFGLAPMLSDTDKKPFTAILYGNGPGYKVVGGERENVSMVDYAHNNYQAQSAVPLRHETHGGEDVAVFSKGPMAHLLHGVHEQNYIPHVMAYAACIGANLDHCAPASSAGSLAAGPLLLALALFPLSILF

>Lemur_Alpi_Scaf_2374:327,736_ENSMICT00000012117[Microcebus_murinus]

MQGSWVLLLLLLLGLRLQLSLCVIPGVEENPAFWNLKAAEALDAAKKLQPIQTMAKNLILFLGDGMGVPTVTAARILKGQLNGNLGPETPLAMDQFPHLALSKTYNVDRQVPDSAGTATAYLCGVKANYQTIGLSAAARLNQCNTTRGNEVLSVMNRAKKAGKSVGVVTTTRVQHASPAGTYAHIVNRNWYSDADMPASALQEGCQDIATQLISNMDIDVILGGGRKYMFPRGTPDPEYPSDASQNGTRLDGKNLVQEWLSTRPGARYVWNRSELIQASLDTSVTHLMGLFEPGDTKYETQRDPELDPSLMEMTEVALRVLSRNPQGFYLFVEGGRIDHGHHNGRAFRALTEAIMFDSTINMTGQLTSEQDTLTLVTADHSHVFSFGGYTFRGSSIFGLAPSKAQDGKAYTSILYGNGPGFALDSGTRPDVSESESEVPTYRQQAAVPLSSETHGGEDVAVFARGPVPAPAAGSLLLATALPPPQASAW

>Lemur_Alpl_Scaf_4864:21,954_ENSMICT00000013922_[Microcebus_murinus]

MISAFLVLAIGTCLANSLVPEKEKDPKYWRDQAQQTLKHALGLQKLNTNVAKNVIMFLGD GMGVSTVTAARILKGQLHHNTGEETKLEMDKFPFVALSKTYNTNAQVPDSAGTATAYLCG VKANEGTVGVSAATERTRCNTTQGNEVTSILRWAKDAGKSVGIVTTTRVNHATPSAAYAH SADRDWYSDNEMPPEALSQGCKDIAYQLMHNIRDIDVIMGGGRKYMYPKNRTDVEYEMDE KARGTRLDGLNLIDIWKSFKPKHKXXXXXXXXXXXXXXXXXXXXXXXXXXXXXXXXXXXX XXXXXXXXXXXXXXXXXQVLRKNPKFFLLVEGGRIDHGHHEGKAKQALHEAVEMDQAIGL ADNMTSLQDTLTVVTADHSHVFTFGGYTPRGNSIGLAPMLSDTDKKPFTAILYGNGPGYK VVGGERENVSMVDYAHNNYQAQSAVPLRHETHGGEDVAVFAKGPMAHLLHGVHEQNYIPH VMAYAACIGANLNHCPGSSAGSPAPGALLLLTCPLAL

>Lemur_Alpi1_Mamul12:96,240,252_ENSMMUT00000009760[Macaca_mulatta]

MRGPWVLLLLLGLRLQLSLGIIPVEEENPDFWNRQAAEALGAAKKLQPIQTAAKNLIIFLGDGMGVSTVTAARILKGQKEDKLGPETPLAMDHFPYVALSKTYSVDKHVPDSAATATAYLCGVKGNFQTIGLSAAARYNQCNTTRGNEVVSVMNRAKKAGKSVGVVTTTRVQHASPAGAYAHTVNRNWYSDANMPGSARREGCKDIATQLISNMDIDVILGGGRKYMFRMGAPDPEYPHDYSQDGTRMDGKNLVQEWLAKQQGARYVWNRTELMQASLDPSVTHLMGLFEPGDMKYEIHRDPTLDPSLMEMTEAALRLLSRNPRGFFLFVEGGRIDHGHHESRAYRALTEAVMHSTAVFSSTTGTPLSAGLAPGKAQDRKAYTALLYGNGPGYVLKDGARPDVTESESGDPEYRQQAAVPLDEETHAGEDVAVFARGPQAHLVHGVQEQSFIAHVMAFAACLEPYTACDLAPPAGTTDTAHPGRSAVPESLPLLAAALLLLGMVTAP

>Macaque_Alpi2_Mamul12:96,279,925_ENSMMUT00000044400[Macaca_mulatta]

SPLPVEEENPDFWNYQAAKALGAAKKLQPAQTAAKNLIIFLGDGMGVSTVTAARILKRQKKGKLGPETPLAMDHFPYVALSKTYSVDKHVPDSAATATAYLCGVKDNFQTIGLSAAARYNQCNTTRSNEVVSVMNRAKKAGKSVGVVTTTRVQHASPAGAYAHTVNRNWYSDTNVPASARREGCKDIATQLISNMDIDVILGGGQKYMFPMGTPDPEYPHDYSQDGTRMDGKNLVQEWLAKQQGARYVWNRTELMQASLDHQGTPCPTGLFEPGDMKYEIHRDPTLDPSLMEMTEAALRLLSRNRRGFFLFVEGGRIHHGHHESRAYRALTEAVMFDDAIERAGQLTSEEDTLTLVTADHSHVFSFGAYPLRGSSIFRLAPGKAQDRKAYTALLYGNGPGYVLKDGARPDVTESQSGSPEYRQQSGVPLDEETHADEDVAVFARGPQAHLVHGVQEQSFVAHVMAFAACLEPYTA

>Macaque_Alpi3_Scaf1099548049583:18,498_ENSMMUT00000023700[Macaca_mulatta]

MQGPWVLLLLGLRLQLSLGIIPAEEENPAFWNRQAAEALDAAKKLQPIQKVAKNLILFLGDGMGVPTVSLVRGRPVCGTAWGASLPGMMPRESWCLYSQTYNVDRQVPDSAGTATAYLCGVKANYQTIGLSAAARYNQCNTTRGNEVISVMNRAKKAGKSVGVVTTTRVQHASPAGTYAHTVNRNWYSDADMPASARQDGCQDIATQLISNVDIDVILGGGRKYMFPRGTPDPEYPSDASQNGIRLDGKYLVQEWLTKHQGARYVWNRTELMQASLDQSVTHLMGLFEPGDMKYEINRDPTLDPSLLEMTEAALRLLSRNPRGFYLFVEGGRIDHGHHEGLAYHALTEAVMFDDAIERAGQLTSEEDTLTLITADHSHVFSFGGYTFRGSSIFGLAPSKAQDSKAYTSILYGNGPGYVLNSGVRPDVNESESGDPDYRQQAAVPLSSETHGGEDVAVFARGPQAHLVHGVQEQSFVAHVMAFAACLEPYTACDLAPPAGTT

>Macaque_Alpl_Mamul1:24,158,856_ENSMMUT00000003136[Macaca_mulatta]

MISPFLVLAIGTCLTNSLVPEKEKDPKYWRDQAQETLKYALELQKLNTNVAKNVIMFLGDGMGVSTVTATRILKGQLHHNPGEETRLEMDKFPFVALSKTYNTNAQVPDSAGTATAYLCGVKANEGTVGVSAATERSRCNTTQGNEVTSILRWAKDAGKSVGIVTTTRVNHATPSAAYAHSADRDWYSDNEMPPEALSQGCKDIAYQLVHNIRDIDVIMGGGRKYMYPKNKTDVEYEIDEKARGTRLDGLDLVNIWKSFKPRHKHSHFIWNRTELLTLDPHNVDYLLGLFEPGDMEYELNRNNVTDPSLSEMVVVAIQILRKNPKGFFLLVEGGRIDHGHHEGKAKQALHEAVEMDRAIGQAGSMTSLEDTLTVVTADHSHVFTFGGYTPRGNSIFGLAPMLSDTDKKPFTAILYGNGPGYKVVGGERENVSMVDYAHNNYQAQSAVPLRHETHGGEDVAVFSKGPMAHLLHGVHEQNYIPHVMAYAACIGANLDHCAPASSAGSLAAGPLLLPLALFPLSILF

>Marmoset_Alpi1_Cajac6:148,870,627ENSCJAT00000026638[Callithrix_jacchus]

MQGLWVLLLLGLRLQLSLGVIPVEEENPGFWNRQAAEALDAAKKLQPIQRVAKNLILFLGDGMGVPTVTATRILKGQSNGKLGPETPLAMDRFPYVALSKTYNVDRQVPDSAATATAYLCGVKGNFQTIGLSAAARFNQCNTTRGNEVISVMNRAKKAGKSVGVVTTTRVQHASPAGTYAHTVNRNWYSDADMPASARQEGCQDIATQLISNMDIDVILGGGRKYMFPTGTPDPEYPSDASQNGIRLDGKNLVQEWLAKHQGAQYVWNCTELMQASLDSSVTHLMGLFEPGDTKYEINRDPTRDPSLMEMTEAALRLLSRNPLGFYLFVEGGRIDHGHHEGIAYHALTEAVMFDDAIERAGQLTSEEDTLTLVTADHSHVFSFGGYTLRGSSVFGLAPSKAQDSKAYTSILYGNGPGYAFSSSVRPDVNESESGDPDYKQQAAVPLSSETHGGEDVAVFARGPQAHLVHGVQEQSFLAHIMAFAACLDPYAACDLAPAAGTTATPPTWLPSRCLCWLGPCCCCWGRPL

>Marmoset_Alpi2_Cjac6:148,802.081_johnbuilt[Callithrix_jacchus_Marmoset]

LGVLQVEEENPAFWNHQAAEALDAAKKLQPAQTAAKNLILFLGDSEGARLSSPQPSRPQHPDCRWFQDSPGAQASHISAPSGMGVSTVTAARILKGQKKGKPGPETPLAMDRFPYVALSKTYSVDKHVPDSAATATAYLCGVKGNFQTIGLSAAARFNQCNTTRGNEVVSVMNRAKKAGELGPLRGQGRVTDLCWVSPLSPSGKSVGVVTTTRVQHASPAGTYAHTVNRNWYSDSNMPFSVRREGCRDIATQLVSNMDIDVILGGGRKYMFRMGTPDPEYPHNYSHDGIRLDGKNLVQEWLAKHQGARYVWNRTELMQASLDLSVTHLMGLFEPGDMKYDIHQDSTRDPSLMEMTEAALCLLSRNPGFFLFVEGGHIEYGHHESRAYRALTE

>Marmoset_Alpi2_Cjac6:148,798,191_ENSCJAT00000026621[Callithrix_jacchus]

LLGPSIISTLLFYGLRLQLSLGIIPVEEENPAFWNHQAAEALDAAKKLQPAQTAAKNLILFLGDRMGVSTVTAARILKGQKKGKPGPETPLAMDRFPYVALSKTYSVDKHVPDSAATATAYLCGVKGNFQTIGLSAAARFNQCNTTRGNEVVSVMNRAKKAGKSVGVVTTTRVQHASPAGTYAHTVNRNWYSDSNMPFSVRREGCRDIATQLVSNMDIDVILGGGRKYMFRMGTPDPEYPHNYSHDGIRLDGKNLVQEWLAKHQGARYVWNCTELMQASLDPSVTHLMDLFEPGDMKYDIHQDSTRDPSLMEMTEAALCLLSRNPXGFFLFVEGGRIDHGHHESRAYRALTETIMFDDTIERAGQLTSEEDTLTLVTADHSHVFSFGGYPLRGSSVYGLAPGEARDGKAYTALLYGNGPGYVLKDGARPDVTESQSGSPEYRQQSAVLLDEETHAGEDVAVLARGPQAHLVHGVQEQSFIAHVLAFAACLEPYEDCDLAPPAGTTDAAYPGPAAVPGSLPLLAGILLLLGAAAAP

>Marmoset_Alpl_Cjac7:55,489,046_ENSCJAT00000011946[Callithrix_jacchus]

MISPFLVLAIGTCLTNSLVPEKEKDPKYWRNQAQETLKYALELQKLNTNVAKNVIMFLGDGMGVSTVTAARILKGQLHHNPGEETRLEMDKFPFVALSKTYNTNAQVPDSAGTATAYLCGVKANEGTVGVSAATERSRCNTTQGNEVTSILRWAKDAGKSVGIVTTTRVNHATPSAAYAHSADRDWYSDNEMPPEALSQGCKDIAYQLMHNIRDIEVIMGGGRKYMYPKNKTDVEYEIDEKARGTRLDGLDLVDIWKNFKPRHKHSHFIWNRTELLALDPYNVDYLLGLFEPGDMQYELNRNNVTDPSLSEMVTVAIQILQKNPRGFFLLVEGGRIDHGHHEGKAKQALHEAVEMDRAIGRAGSMTSLEDTLTVVTADHSHVFTFGGYTPRGNSIFGLAPMLSDTDKKPFTAILYGNGPGYKVVGGERENVSMVDYAHNNYQAQSAVPLRHETHGGEDVAVFSKGPMAHLLHGVHEQNYIPHVMAYAACIGANLDHCAPASSARNLAAGPLLLPLALF
